# Effectiveness of community-based delivery of mass dog vaccination to prevent rabies: A cluster randomized controlled trial

**DOI:** 10.1371/journal.pntd.0014704

**Published:** 2026-09-25

**Authors:** Felix Lankester, Ahmed Lugelo, Joel Changalucha, Danni Anderson, Christian Tetteh Duamor, Anna Czupryna, Kennedy Lushasi, Elaine Ferguson, Emmanuel S. Swai, Maganga Sambo, Jonathan Yoder, Sarah Cleaveland, Sally Wyke, Benezeth Lutege Malinda, Paul C. D. Johnson, Katie Hampson

**Affiliations:** 1 Paul G. Allen School for Global Health, Washington State University, Pullman, Washington, United States of America; 2 Rabies Free Africa, Global Health Tanzania, Arusha, Tanzania; 3 Environmental Health and Ecological Sciences Department, Ifakara Health Institute, Ifakara, Tanzania; 4 Department of Veterinary Medicine and Public Health, College of Veterinary Medicine and Biomedical Sciences, Sokoine University of Agriculture, Morogoro, Tanzania; 5 School of Biodiversity, One Health and Veterinary Medicine, University of Glasgow, Glasgow, United Kingdom; 6 Department of Global Health and Biomedical Sciences, Nelson Mandela African Institute of Science and Technology, Arusha, Tanzania; 7 Directorate of Veterinary Services, Ministry of Livestock Development and Fisheries, Dodoma, Tanzania; 8 School of Social and Political Sciences, University of Glasgow, Glasgow, United Kingdom; 9 School of Economic Studies, Washington State University, Pullman, Washington, United States of America; University of Abuja Faculty of Veterinary Medicine, NIGERIA

## Abstract

**Background:**

Approximately 60,000 people die from rabies annually, mostly in Africa and Asia. Mass dog vaccination is critical for elimination. Herd immunity has been calculated to be attained when >40% (critical threshold) of dogs are vaccinated. A common delivery method implemented in Tanzania and other countries typically involves non-local teams travelling to villages implementing dog vaccination annually through static-point clinics using cold chain stored vaccines (Team-based delivery). Determination that a canine rabies vaccine is thermotolerant enables novel Community-based dog vaccination strategies whereby community vaccinators use vaccines stored locally throughout the year (Community-based delivery).

**Methods:**

Two dog vaccination strategies were compared using a three-year cluster randomized controlled trial in Tanzania (Clinical Trials Registration Number ISRCTN14813279). The strategies were designed to represent implementation using resources typical of government-led initiatives. Wards (*n* = 112) were randomly assigned to either Team-based delivery or Community-based delivery. Coverage was estimated using household surveys implemented biannually.

**Findings:**

Mean coverage achieved by Community-based delivery (55%; 95% Confidence Interval (CI): 45–65%) was higher than Team-based delivery (37%; 95% CI: 28–46%). Moreover, because coverage was lower and declined over each year only in Team-based delivery, the probability of coverage being below the critical threshold was higher for Team-based delivery (60%) compared to Community-based delivery (18%). To ensure coverage does not dip below the critical threshold by the year end, coverage at the start of the year in Team-based delivery needed to be higher (61%) than in Community-based (42%).

**Interpretation:**

Community-based delivery achieved higher and more consistent vaccination coverage across a range of settings typical of many sub-Saharan African countries. Generalisation to different sociocultural/ agroecological settings requires further evidence. Although direct evidence on impact on rabies elimination was not measured, this approach could play a role in national elimination strategies, developed for the global ‘Zero by 30’ strategy to end dog-mediated rabies deaths by 2030, and in response to investment by Gavi, the vaccine alliance.

## Author summary

Dog-mediated human rabies is a lethal infectious disease and kills approximately 60,000 people annually, primarily in Africa and Asia. Mass dog vaccination is a proven approach to prevent human rabies by targeting the reservoir host, the domestic dog, but logistical challenges and high costs hinder implementation. Recent demonstration that a commonly used canine rabies vaccine could be stored outside of refrigeration units at 30°C for three months without loss of potency provided the foundation for the roll out of a novel approach to mass dog vaccination, called Community-based delivery, which uses vaccines stored in and managed by local communities. This study describes a randomized controlled trial to compare the effectiveness of a mass dog vaccination delivery approach (implemented using resources typical of government-led initiatives) that is commonly implemented in Tanzania and other rabies endemic countries by centralized vaccination teams (Team-based) with the novel decentralized (Community-based) delivery. Our findings demonstrate that Community-based delivery achieves higher and more consistent vaccination coverage than the Team-based approach in the rural and urban settings in which the trial took place. Although data on rabies elimination was not collected, mass dog vaccination has been shown to be an effective method to control rabies. As such, by overcoming key logistical barriers, for example needing to store vaccines under refrigeration conditions, this strategy could provide a scalable solution to eliminate dog-mediated rabies in resource-limited settings.

## Introduction

Dog-mediated human rabies has the highest case fatality rate of any known infectious disease and kills approximately 59,000 people annually [1,2]. Over 99% of these fatalities occur in Africa and Asia, where access to post-exposure prophylaxis (PEP) is poor and where limited investment in effective preventive measures means that the burden of the disease in endemic countries remains high [1,3–6]. Recognizing this, the World Health Organization (WHO), the Food & Agricultural Organization (FAO) and the World Organization for Animal Health (WOAH) have classified human rabies as a global health priority and have united in a commitment to its global elimination by 2030 (Zero by 30) [7].

Human rabies can be controlled through expedient PEP use. However, it is costly and not readily accessed by those at risk. A more equitable approach to control is through mass dog vaccination, which, by targeting the primary reservoir responsible for >99% of human cases, provides protection to everybody irrespective of their socio-economic status. However, implementing dog vaccination is logistically challenging [8,9].

In Africa, Asia, and Latin America dog vaccination often relies on annual team-based vaccination campaigns. These campaigns are typically conducted by teams of vaccinators who are often based where electricity and refrigerators enable cold-chain vaccine storage [8,10]. These teams of vaccinators typically travel to target communities with sufficient vaccines for the days activities where they implement mass vaccination of the local dog population. The aim of these annual campaigns is to vaccinate at least 70% of the dog population as this will prevent vaccination coverage dropping, before the team returns the following year, below the critical threshold (40%) below which herd immunity is predicted (through a quantitative modelling analysis of the transmission dynamics of rabies in domestic dogs exploiting the basic reproductive number (R_0_)), to be lost [11]. Logistical constraints mean that achieving this coverage consistently is difficult [12–15]. Indeed, even small gaps in coverage across a landscape significantly hinder elimination [16,17], underscoring the need for innovative cost-effective and scalable dog vaccination strategies that provide consistent levels of coverage above the critical threshold.

Community-based delivery strategies have been used for controlling other neglected tropical diseases like onchocerciasis. However, due to numerous factors such as regulatory, clinical, biosafety as well as the barrier of cold-chain vaccine storage, community-based approaches remain untested for rabies [18]. Recent determination of rabies vaccine thermotolerance overcomes one of these barriers allowing long-term storage at relatively high temperatures [19,20] and, like other mass programs that have benefited from thermotolerant vaccines (meningitis A, hepatitis B, Newcastle disease, smallpox and rinderpest) [21–27], provides hope that cost-effective rabies control programs targeting communities where rabies remains endemic might be possible through community-based strategies.

The aim of the reported study was to compare a novel (Community-based) delivery strategy that exploits local storage of vaccines and is implemented by locally based personnel against an approach that uses vaccines stored under cold-chain conditions in a central location where power is available and is implemented by teams of vaccinators who are not based in the local community (Team-based). The Team-based approach was designed to reflect typical team-based vaccination strategies employed by governments, such as the Government of the Republic of Tanzania, to implement mass dog vaccination. The Team-based approach was not designed to reflect the approach commonly used by non-governmental organisations that often have the resources needed to implement post-vaccination coverage assessments and targeted follow-up vaccination efforts.

Care was taken to ensure as far as possible that both arms of the trial were subject to the same resource constraints. To achieve this, a pilot study was implemented in 2019–2020 in which both the Team- and Community-based strategies were trialled, and the feasibility of storing vaccine locally under non-cold chain conditions within locally made passive cooling devices was tested [28,29]. The pilot allowed the resources required to implement the strategies to be estimated so that programmatic costs, such as for personnel time, travel and communication, could be quantified. The aim was that sufficient and comparable resources would be provided for each strategy, including stipends following government guidelines, to accomplish the respective activities and that, following the establishment of these resource input levels, the research team would not specify resource constraints differentially in favor of one arm or the other.

The comparison of the two approaches was performed using the rigor provided by a large-scale randomized controlled trial (RCT). The objective of the RCT was to compare the effectiveness of these two complex interventions and to evaluate them as they would be delivered in the real world. In this framing, resource allocation differences between arms that are subject to the same overall resource constraints are not a confounder but are part of the intervention package. The primary and secondary outcome measures were coverage and the probability of coverage falling below the critical threshold. In parallel to the primary and secondary measures, cost measures were collected for economic analyses and cost differences between the interventions were quantified, while Integrated Bite Case Management was implemented to evaluate the public health impacts of mass dog vaccination. The cost-effectiveness analysis has been published [30] and the public health analyses will be published in due course.

The results have the potential to influence rabies control strategies across Tanzania and similar regions in Africa where limited access to power and cold-chain storage hinder rabies elimination efforts.

## Methodology

### Ethics

The trial was approved for human subjects research by the National Health Research Ethics Committee (Ref: NIMR/HQ/R.8a/Vol. IX/2788). Ethical approval was obtained from the Tanzania Commission for Science and Technology, the Ministry of Regional Administration and Local Government and the Institutional Review Board of Ifakara Health Institute (IHI/IRB/No:24-2018). Written formal consent was obtained from all participants.

### Study area

An RCT was conducted in six districts within the Mara region, northern Tanzania (Fig 1). Each district consists of between eight and 30 administrative wards, each comprising approximately three to four villages. Only wards that had not been previously targeted by a donor-funded annual mass dog vaccination (n = 178) were eligible for inclusion (Fig 2). Following randomized selection and allocation of wards to one of two arms, the RCT was implemented in 112 wards (56 in each arm), which was the number required from the sample size calculations. Using geographical characteristics, population densities and the proportion of the inhabitants that were engaged in non-farming activities, these 112 wards were classified by the National Bureau of Statistics as either rural (*n* = 90 (80%)) or urban (*n* = 22 (20%)) [31]. However, the selection of wards in this trial was not stratified according to this classification.

**Fig 1:**
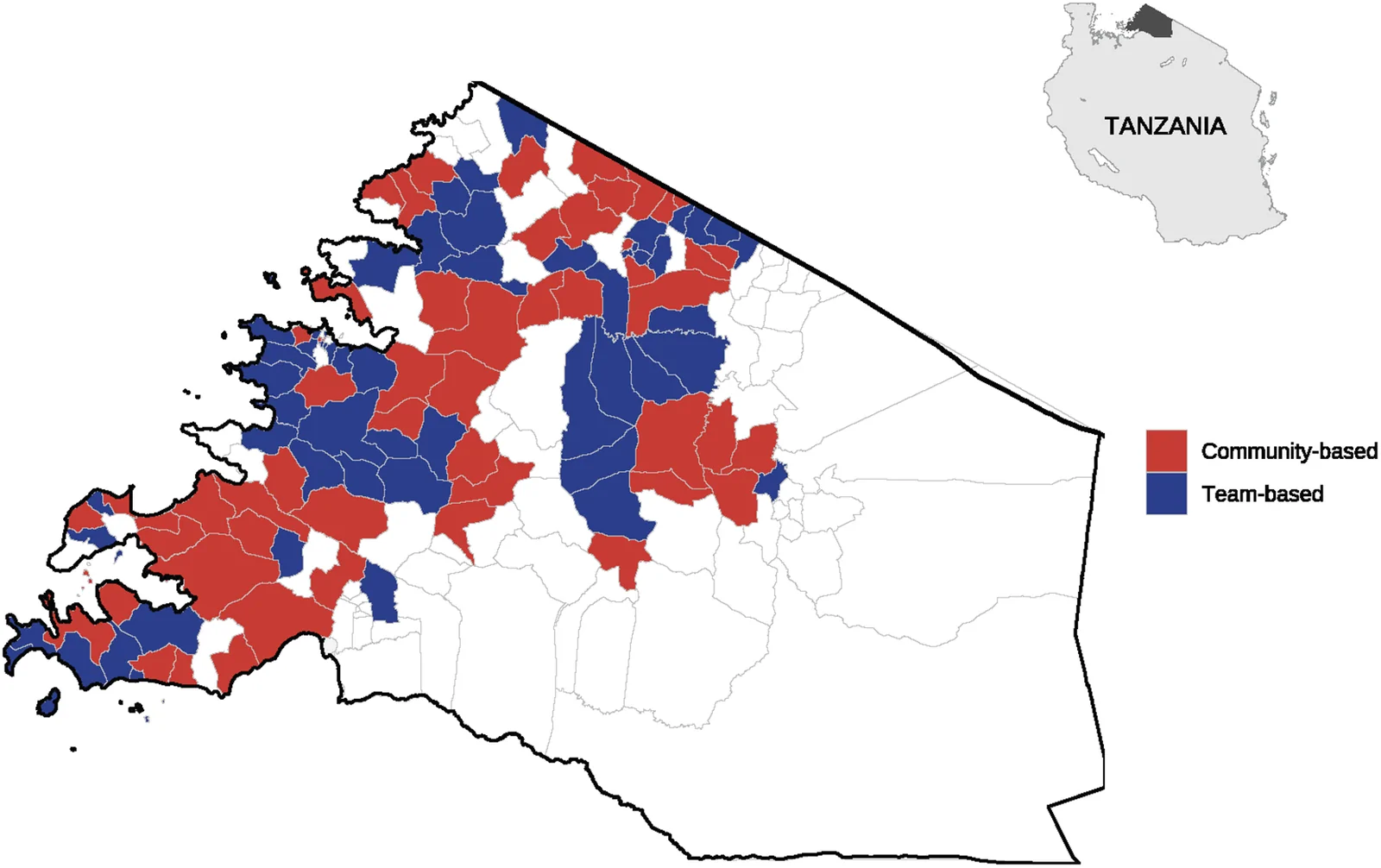
Study area showing the wards of the Mara region, northern Tanzania, where the randomized controlled trial (RCT) of mass dog vaccination was conducted (red = community-based and blue = team-based). The base layer of the map was provided by GADM (https://www.gadm.org).

**Fig 2:**
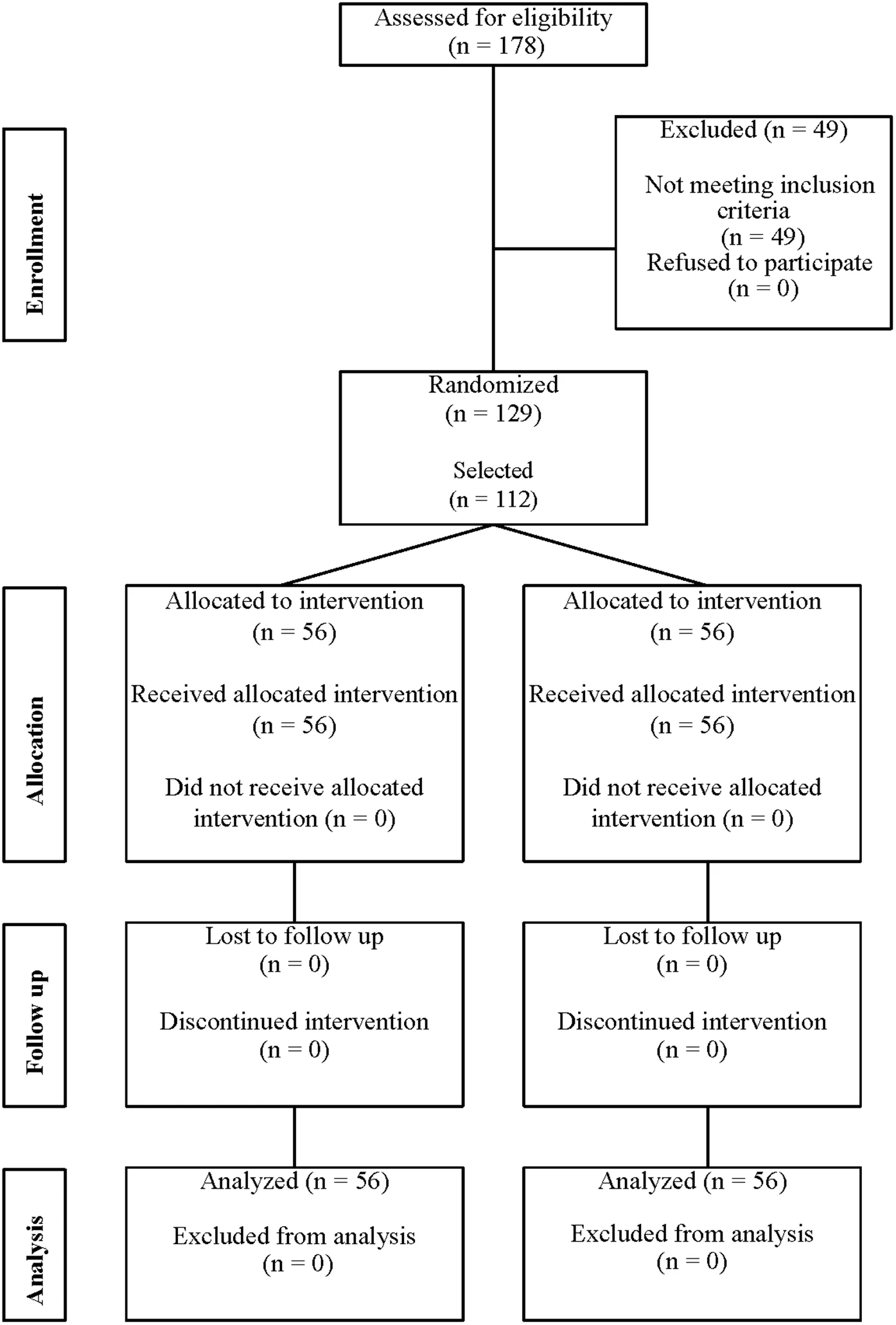
CONSORT flow diagram showing the flow of participating wards through each stage of the randomized trial.

### Study design

The study was carried out using a stratified parallel cluster RCT repeated over three annual cycles (November – October, 2020–2023). The trial was stratified at the administrative district level and clustered at the administrative ward level. We employed a ‘fried-egg’ approach to prevent contamination (spill-over) of intervention effects between trial arms [32]. All villages in each ward received the allocated treatment but coverage was measured only in the most central village (‘egg-yolk’). The central village was selected by the research team as the village that had the weakest connection (least common borders and furthest distance) with neighbouring ward(s). This assessment was done by eye. Randomisation was implemented using a custom R script [33]. To enable management of the trial, the random allocation of wards to trial arms was revealed to personnel, however the allocation was not revealed to the study statistician.

### Interventions being tested

The study tested the effectiveness of two dog vaccination strategies, **centralized pulsed team-based delivery** (Team-based delivery) and **decentralized continuous community-based delivery** (Community-based delivery).

### Trial protocol

The trial protocol (ISRCTN14813279) can be found in S1 Protocol.

### Intervention teams

In both arms, vaccination campaigns were managed by the District Veterinary/ Livestock Field Officer responsible for animal-related matters. (In Tanzania, Livestock Field Officers are certified government employed veterinary paraprofessionals who are legally permitted to treat animals and administer vaccinations.) In both arms equipment, communication and transport allowances, and comparable stipends (following government guidelines), were provided to ensure activities could be accomplished effectively. These resources were managed by the arm-specific personnel themselves.

In Team-based delivery, the vaccination team was designed to reflect the team structure that is typically used by the Government of Tanzania to implement mass dog vaccination. As such, the team operated through a three-person team comprised of the Senior Livestock Field Officer for the district who, for each target ward, teamed up with the ward-based Livestock Field Officer and, for each village, a local assistant who lived in the village and could assist the team by encouraging dog owners to bring their dogs and with other practical matters during the campaign. At the start of the RCT, the Senior Livestock Field Officer received training on data collection using a smartphone app and on coordination/ implementation of dog vaccination using Team-based delivery [34].

The Community-based delivery model was designed to reflect an alternative vaccination delivery structure that utilised community-based personnel. As such, this model operated through a three-person team composed of a Ward Livestock Field Officer (referred to as the Rabies Coordinator), a community leader (the One Health Champion), and a local assistant. Vaccination activities were implemented within each ward under the coordination and supervision of the Rabies Coordinator, who oversaw all vaccination operations across their villages, managed local vaccine storage, and organized the scheduling and execution of vaccination campaigns. All Rabies Coordinators received formal training on the coordination and implementation of dog vaccination using the community-based approach. Within each village, the One Health Champion worked closely with the Rabies Coordinator, leveraging their understanding of local households to support targeted vaccination. The One Health Champion also facilitated community engagement by granting permission for vaccination activities and sharing information about local events that could influence the campaign’s scheduling or attendance. The local assistant supported the team primarily during the early phase of the annual vaccination calendar when dog turnout was high and additional help was required for registration. Their main task involved recording vaccinated dogs in the official register. Once the initial phase ended and turnout declined, the assistant’s involvement was concluded, leaving the Rabies Coordinator and the One Health Champion to continue the follow-up vaccination campaigns.

### Implementation of the intervention - A comparison of Team- and Community-based delivery strategies

The comparative operational characteristics of the two delivery strategies is shown in Table 1. In both delivery methods, the respective teams implemented a single day static-point clinic positioned at a central and easily accessible location within the target village. Thereafter, in Team-based delivery the village would not be targeted again until the next annual cycle typical of the government-run delivery approach the strategy was designed to emulate. By contrast, because vaccines were stored throughout the year at the ward level, Community-based delivery vaccination activities could be carried out throughout the year by each village’s implementing team. These activities took the form of static-point clinics hosted at the sub-village level, house-to-house vaccinations targeting households that the implementing team knew to have unvaccinated dogs, and on-demand vaccination whereby dog owners contacted the implementing team and requested that their dog be vaccinated.

**Table 1:** Operational comparison table summarising the practical differences between the two delivery strategies.

Characteristic	Trial Arm	
	Team-based	Community-based
Management authority	District Veterinary/ Livestock Field Officer	District Veterinary/ Livestock Field Officer
Equipment	Provided by research team	Provided by research team
Communication and transport allowances	Provided by research team	Provided by research team
Stipends	Provided by research team following government guidelines	Provided by research team following government guidelines
Team structure	Three-person team: District Senior Livestock Field Officer, Livestock Field Officer, local assistant	Three-person team: Ward Livestock Field Officer, community leader, local assistant
Vaccine storage	District-level storage	Ward-level storage
Vaccination strategy	Month 1: Village-level static point clinic	Month 1: Village-level static point clinic Months 2 – 12: sub-village-level static point clinics, house-to-house, on-demand vaccinations
Advertising	Formal communication from district- to village-level leadership; posters and word of mouth at village-level	Posters, social networks, community meetings and word of mouth at village-level
Monitoring and evaluation	District Veterinary/ Livestock Field Officers	District Veterinary/ Livestock Field Officer, Rabies Coordinators and community leadership committees

### Implementation of the intervention - Specifics of Team-based delivery

A week prior to each village-based vaccination day community sensitization efforts were undertaken to raise awareness and encourage attendance. The process began with a formal communication from the Ministry of Livestock and Fisheries Section, which was disseminated hierarchically, from the Head of Livestock Section to the District Executive Director, followed by the Ward Executive Officer, Village Executive Officer, Village Chairperson, Sub-village Chairpersons (Balozi), and ultimately reaching individual community members. At the grassroots level, community leaders disseminated information primarily through word of mouth and by posting official notices at village offices. This flow of communication mobilized residents and ensured that community members were well informed about the timing and location of the vaccination exercise. The vaccination equipment, including vaccine stored in refrigerators, was taken from, and returned to, the district headquarters each day. At the clinic, the Senior Livestock Field Officer registered each dog in the smartphone application and administered a subcutaneous 1-mL dose of Nobivac Rabies vaccine. The ward-based Livestock Field Officer verified and certified the vaccinated dogs, while the local assistant recorded the details in the official vaccination register. Each clinic operated for a full day. The following day another village was targeted until all villages in a ward were vaccinated. After completing a ward, the Senior Livestock Field Officer targeted another ward that was selected to receive vaccination through Team-based delivery. Through this method all Team-based delivery wards received dog vaccination. This method was repeated annually for three years across these wards. Detailed information of this delivery can be found in S1 Table.

### Implementation of the intervention - Specifics of Community-based delivery

Prior to the onset of dog vaccination activities, the Rabies Coordinators for each Community-based delivery ward travelled by motorbike to the District Veterinary Office to collect vaccination equipment and a batch of cold-chain stored vaccines estimated to be sufficient for three months of vaccination activity within their respective wards. Upon arrival at their ward, the Rabies Coordinator stored their ward’s vaccines in a locally made low-tech cooling device (Zeepot) [35]. One week before the vaccination campaign, the One Health Champions in each village of the ward displayed posters in strategic locations within their respective villages, providing details on the date and venue of the upcoming vaccination. They also disseminated information through their social networks, community meetings, and word of mouth. Vaccination activities were implemented by the Rabies Coordinator across all villages within their respective wards by targeting one village at a time. On vaccination day for a specific village within their ward, the Rabies Coordinator travelled by motorbike to the target village carrying equipment and sufficient doses for the day’s activities. The Rabies Coordinator registered dogs in the smartphone application and administered a subcutaneous 1-mL dose of Nobivac Rabies vaccine. The One Health Champion for that specific village assisted the Rabies Coordinator by recording details of vaccinated dogs on the vaccination certificates, which were subsequently reviewed and signed by the Rabies Coordinator. The local assistant supported the team by entering dog registration information into the official vaccination register. Each clinic operated for a full day. The following day, the Rabies Coordinator proceeded to the next village within their ward, employing the same approach until all villages in the ward had been targeted.

Because vaccines were stored locally throughout the year, follow-up vaccination efforts could be conducted by the Rabies Coordinators. These were typically hosted at three, six, and nine months in each yearly cycle. These activities employed a combination of sub-village clinics, house-to-house visits, or on-demand vaccination. Through this method all Community-based delivery wards received dog vaccination. This method was repeated annually for three years across these wards. S2 Table contains detailed information on this delivery approach.

In both Team- and Community-based approaches monitoring and evaluation of vaccination outcomes was implemented. In Team-based delivery this was done by District Veterinary/ Livestock Field Officers, and in the Community-based delivery by District Veterinary Officers, Rabies Coordinators and community leadership committees. This monitoring enabled vaccination to be reconfigured where necessary to suit needs [29].

### Vaccination coverage assessment

Household surveys, gathering data on the number of dogs, their vaccination status, reasons for non-vaccination, and opinions on the vaccination services, were conducted in the central study village of all 112 wards. The aim was to survey 30 dog owning households per study village (ten households in each of three randomly selected sub-villages per study village). In each yearly cycle (Y_1_, Y_2_, Y_3_), each study village was surveyed at two time points (Fig 3): Visit One (V_1_) implemented in (approximately) months two to three following the static-point vaccination and Visit Two (V_2_) in months ten to 11. V_1_ surveys assessed coverage soon after static-point clinic completion whilst V_2_ evaluated coverage towards the end of the yearly cycle. This resulted in six survey visits (Y_1_V_1_ to Y_3_V_2_). Survey teams comprised an interviewer and a local guide to assist with locating households. The survey team began with a household on the periphery and then moved towards the centre of the sub-village selecting every fifth household. If a household had no adult or dogs present it was skipped and the next chosen until ten were surveyed per sub-village. If fewer than ten dog-owning households were found, additional sub-villages were included. Interviews were conducted in Swahili.

**Fig 3:**
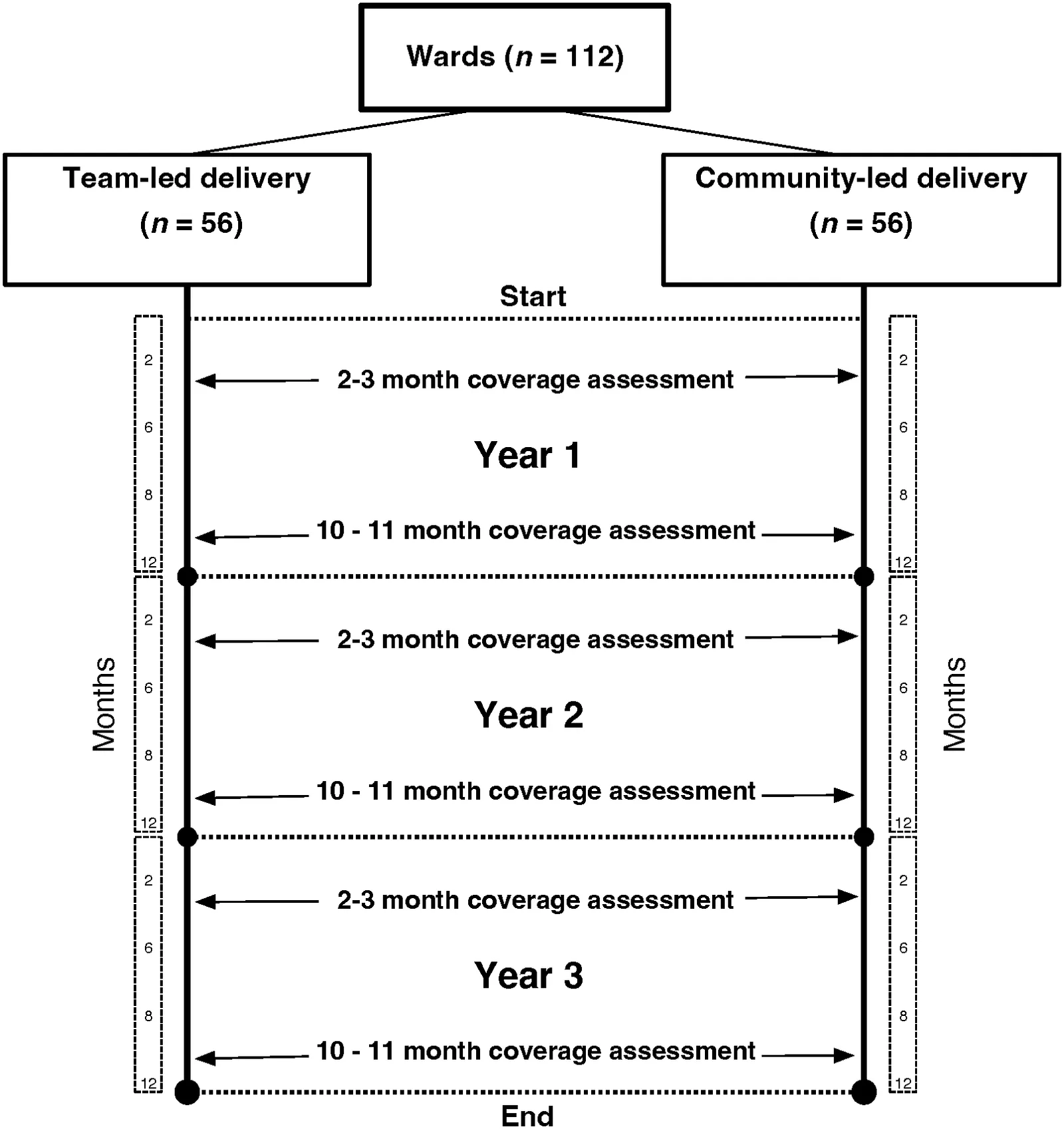
Flow chart showing the two mass dog vaccination delivery approaches compared within the three-year RCT, and when the annualised coverage assessments occurred within each annual cycle. Detailed descriptions of each delivery approach have been given in the main text.

### Data analysis

#### Outcome measure.

The outcome measure was vaccination coverage recorded at each of the six household survey visits. Vaccination coverage was defined as the count of dogs with a fully or partially completed vaccination certificate divided by the sum of dogs with vaccination certificates and dogs without vaccination certificates. Dogs claimed by their owner to be vaccinated but where a vaccination certificate could not be produced were excluded from both numerator and denominator. To assess the sensitivity of the trial results to this assumption, the primary analysis was repeated with a stricter definition of vaccination coverage. In this analysis, dogs reported as vaccinated but lacking a certificate were considered unvaccinated and were included in the denominator but not the numerator.

#### Primary analysis.

The primary analysis tested the null hypothesis of equal coverage (Team-based delivery:Community-based delivery odds ratio = 1) against the two-sided alternative hypothesis of unequal coverage. The full model, including the main effect of trial arm and the *arm × year*, *arm × visit*, and *arm × year × visit* interactions, was compared with a null model not including trial arm or its interactions with visit (V_1-2_) or year (Y_1-3_). Mean coverage across the duration of the trial and 95% confidence limits were estimated per arm from 10,000 parametric bootstrap samples (see section *The probability of coverage falling below 40%* and S3 Table for details).

#### Secondary analyses.

**Vaccination coverage at V**_**2**_: To test the hypothesis that vaccination coverage at the end of the annual cycle was higher under Community-based delivery, coverage at V_2_ was compared between arms.

**The probability of coverage falling below 40%:** The probability of coverage in a ward falling below the critical threshold, along with 95% confidence intervals, was estimated for both study arms, by year and by month. To analyze temporal trends, the primary model was refitted, substituting survey visit categories with visit dates, and monthly coverage was predicted assuming a logit-linear trend per arm per year. For each month, the probability of falling below 40% coverage was estimated as the proportion of ward-level coverage values below this threshold across a distribution approximated by 10,000 parametric bootstrap draws. These draws incorporated both sampling uncertainty in the model parameters and random variation between wards and over time, which was incorporated by sampling from estimated location-specific and time-point-specific ward and district random effect variances.

**Temporal variation in coverage:** To determine consistency of coverage above 40%, three aspects of temporal variation were analyzed for each arm:

- ***Consistency over time:*** Mean coverage consistency across three years was compared using odds ratios of inter-survey variation.
- ***Coverage change between visits:*** Coverage changes from V_1_ to V_2_ were compared. Interactions across visits, arms, and years were tested to justify pooled analyses.
- ***Minimum initial coverage:*** Using the rate of coverage decay recorded in Team-based delivery (due to dog mortality and births), the start-of-year coverage required to maintain coverage above the 40% critical threshold by year-end was estimated.

**Spatial variation in coverage:** Inter-ward consistency was analyzed by comparing coverage variability between arms. The posterior distributions of this modified model with separate inter-ward variance components per arm were estimated.

### General statistical methods

Analyses were performed using a generalized linear mixed-effects regression model (GLMM), fitted using maximum likelihood, except for the analysis assessing spatial variation in coverage, where the GLMM was fitted using Markov Chain Monte Carlo (MCMC). Logit-normal random effects were fitted at four levels: districts, wards, sub-villages, and households. To account for urban areas in which dog ownership and demographic patterns are different from rural areas^31^ when fitting the district-level random effect, three of the six districts were split into two, giving nine levels: Bunda (Bunda Town Council, Bunda District Council); Musoma (Musoma, Musoma Municipal); and Tarime (Tarime Town Council, Tarime District Council). Two types of random effect were fitted at each level (except household level): i) location-specific random effects that allowed variation among locations that was consistent over time and ii) time-point-specific random effects to allow variation in coverage that was not consistent over time. Because approximately a third of households were resampled between survey visits, only a time-point-specific random effect was fitted for the household level. The household random effect was not included in the sensitivity analyses using a stricter definition of vaccination coverage due to showing symptoms of non-identifiability (a very large variance estimate and slow convergence). Intervention arm, survey visit (V_1_ and V_2_) and year (Y_1_-Y_3_) were modelled as categorical fixed effects. All three two-way interactions and a three-way interaction between year, survey visit, and trial arm were fitted. For the three secondary analyses that concentrate on change in coverage from V_1_ to V_2_, models were simplified by backwards elimination of non-significant terms (see S3 Table for details of all analyses and models).

We tested for spatial autocorrelation among the mean ward-level residuals from the primary analysis model by calculating Moran’s *I* across a range of spatial scales from 1 to 10 nearest neighbours. Nearness was defined using the great circle distance between ward centroids, which were calculated as the means of the coordinates of their constituent subvillages. There was no significant spatial autocorrelation at any scale (*p* > 0.1; S4 Table).

Null hypotheses were tested using likelihood ratio tests (LRT) and rejected at the 5% significance level if *p* < 0.05. Intervention effects were estimated from the primary analysis GLMM as Team-based delivery:Community-based delivery odds ratios. Coverage was also estimated from the GLMM. Estimates ± 95% confidence limits were calculated as logit^-1^[(μ ± zs_μ_)c] for coverage and exp[(β ± zs_β_)c] for odds ratios, where c = √{1 + [16√3/(15π)]^2^V}^-1^, μ and β are the maximum likelihood estimates of the log odds of coverage and Community:Team log odds ratio respectively, s_μ_ and s_β_ are their standard errors, z is the 97.5% quantile of the standard normal distribution, V is the sum of the GLMM random effect variance estimates, and c is a bias-correction factor [36,37].

### Randomisation and sample size calculation

We randomized 112 wards 1:1 to Team-based or Community-based vaccination using sequences generated by a custom R script written by the trial statistician. Randomisation was stratified by district (six strata), employing permuted blocks of size six within each stratum. The sequence was generated by an independent statistician (Theo Pepler, University of Glasgow), using a random seed selected by him. The trial statistician remained blinded throughout the trial, until after the completion of the analysis program. The team implementing the interventions was not blinded due to the nature of the interventions.

We estimated that randomizing 56 wards to each arm of the trial would give 87% power at the 5% significance level to detect a difference in mean coverage between Team- and Community-based delivery, assuming mean coverage (the mean of coverage at V_1_ and V_2_) of 50% with Team-based and 58% with Community-based delivery (equivalent to an odds ratio of 1.34). Mean coverage at V_1_ in both arms was assumed to be 60% following static-point vaccination clinics. Mean coverage was assumed to decline in the Team-based arm to 41% at V_2_ due to deaths and births (assuming an exponentially distributed lifespan with a mean of 26 months), and was assumed to decline less sharply to 55% in the Community-based arm due to ongoing vaccination [38].

### Trial data, code and registry

All trial data and code are publicly available in a Zenodo repository (https://doi.org/10.5281/zenodo.22179133). The trial was registered on the 14th April, 2020, at the United Kingdom’s clinical study registry, ISRCTN (https://www.isrctn.com/ISRCTN14813279).

## Results

### Programmatic results

The characteristics of the districts and the randomised wards, overall and by trial arm, are given in Table 2. In total 186,931 dogs were vaccinated during the three-year RCT (between 1^st^ November 2020 and 31^st^ October 2023) and 25,677 dogs had their vaccination status confirmed through household surveys (Fig 4), with 99% of planned surveys being implemented in both trial arms, 95% of surveys reaching at least 20 dog-owning households within targeted villages, and a mean of 27 dogs per village included in each survey. On average 1,669 dogs were vaccinated in each of the 112 study villages, with a mean of 1,050 and 2,289 dogs vaccinated in the Team-based and Community-based arms, respectively. Vaccination coverage by ward and trial arm over the six survey time points is shown in S1 and S2 Figs.

**Table 2:** Characteristics of randomised wards, overall and by trial arm.

		Total (n = 112)	Trial Arm	
			Team-based (n = 56)	Community-based (n = 56)
District	Bunda	15 (13%)	7 (12%)	8 (14%)
	Butiama	14 (12%)	7 (12%)	7 (12%)
	Musoma	28 (25%)	15 (27%)	13 (23%)
	Rorya	17 (15%)	8 (14%)	9 (16%)
	Serengeti	13 (12%)	7 (12%)	6 (11%)
	Tarime	25 (22%)	12 (21%)	13 (23%)
Land use	Rural	83 (74%)	37 (66%)	46 (82%)
	Peri-Urban	7 (6%)	5 (9%)	2 (4%)
	Urban	22 (20%)	14 (25%)	8 (14%)
N dogs surveyed in central study village of each ward	Mean (SD)	164 (55)	165 (49)	163 (61)
	Median (IQR)	162 (122, 192)	171 (122, 190)	159 (122, 192)
	[Range]	[49, 444]	[76, 301]	[49, 444]
Survey completeness (%)	Mean (SD)	98.7 (5.1)	98.2 (6.1)	99.1 (3.8)
	Median (IQR)	100.0 (100.0, 100.0)	100.0 (100.0, 100.0)	100.0 (100.0, 100.0)
	[Range]	[66.7, 100.0]	[66.7, 100.0]	[83.3, 100.0]
N dogs per ward (estimated)*	Mean (SD)	2466 (1082)	2398 (1142)	2534 (1024)
	Median (IQR)	2420 (1804, 2954)	2280 (1723, 2954)	2493 (2057, 2933)
	[Range]	[143, 6634]	[339, 6634]	[143, 5522]
N vaccinations per ward over 3 years	Mean (SD)	1669 (961)	1050 (484)	2289 (920)
	Median (IQR)	1584 (957, 2198)	1002 (676, 1305)	2217 (1756, 2684)
	[Range]	[139, 5160]	[167, 2096]	[139, 5160]

*The number of dogs per ward was estimated as the 2022 census human population size per ward ÷ human:dog ratio (HDR), where HDR = 18.34 for wards in Musoma Municipal district and HDR = 7.09 elsewhere. HDRs were estimated from a survey of 27,400 households conducted in the Mara region in 2022 [39].

**Fig 4:**
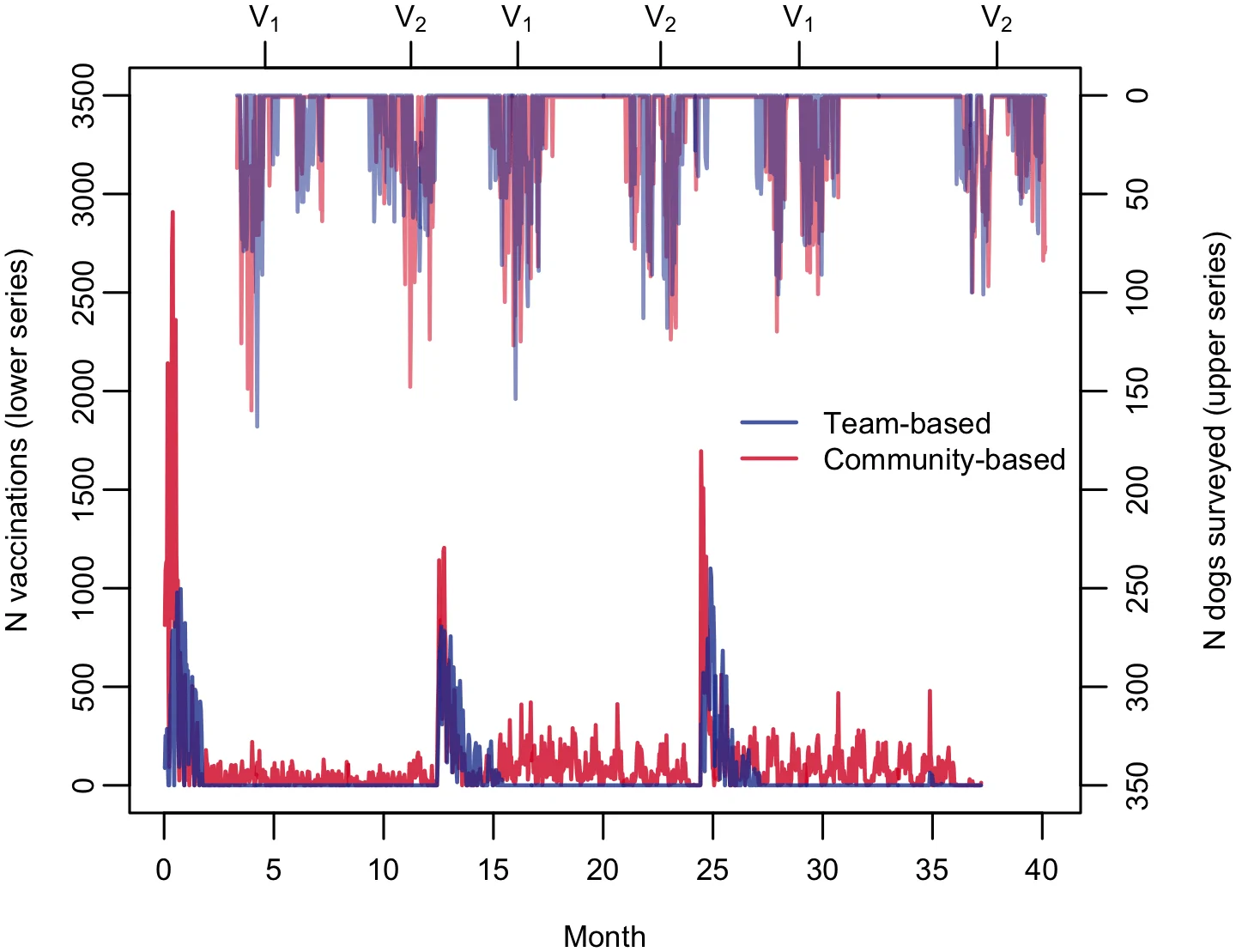
Number of vaccinations performed (lower series; total = 186,931) and number of dogs surveyed (upper series; total = 25,677) per day by trial arm.

### Primary analysis

*Mean coverage.* Mean coverage for each arm at each time point and averaged across the duration of the trial is given in Table 3. Mean coverage differed between arms across the six survey time-points (*p* < 0.001), and was higher with Community-based delivery (55%; 95% CI: 45–65%) than with Team-based delivery (37%; 95% CI: 28–46%). Across the three years the mean coverages ranged in V_1_ from 32% to 46% and 50% to 62% and in V_2_ from 22% to 31% and 49% to 57% in the Team-based and Community-based arms, respectively (Fig 5).

**Table 3:** Coverage and intervention odds ratio estimates (95% CI) at each survey timepoint estimated from the primary analysis GLMM (N dogs surveyed = 18,358).

Year-visit	Coverage % (95% CI)		Odds ratio (95% CI)	*p*-value
	Team-based	Community-based		
Y_1_-V_1_	32 (25, 41)	53 (44, 62)	2.36 (1.74, 3.20)	<0.001
Y_1_-V_2_	22 (16, 29)	49 (40, 58)	3.49 (2.53, 4.81)	<0.001
Y_2_-V_1_	40 (32, 49)	50 (41, 58)	1.48 (1.10, 2.01)	0.011
Y_2_-V_2_	31 (24, 40)	57 (48, 65)	2.90 (2.13, 3.93)	<0.001
Y_3_-V_1_	46 (37, 54)	62 (53, 70)	1.96 (1.45, 2.66)	<0.001
Y_3_-V_2_	31 (24, 39)	54 (45, 63)	2.62 (1.93, 3.56)	<0.001
All years	37 (28, 46)	55 (45, 65)	2.09 (1.58, 2.75)	<0.001

**Fig 5:**
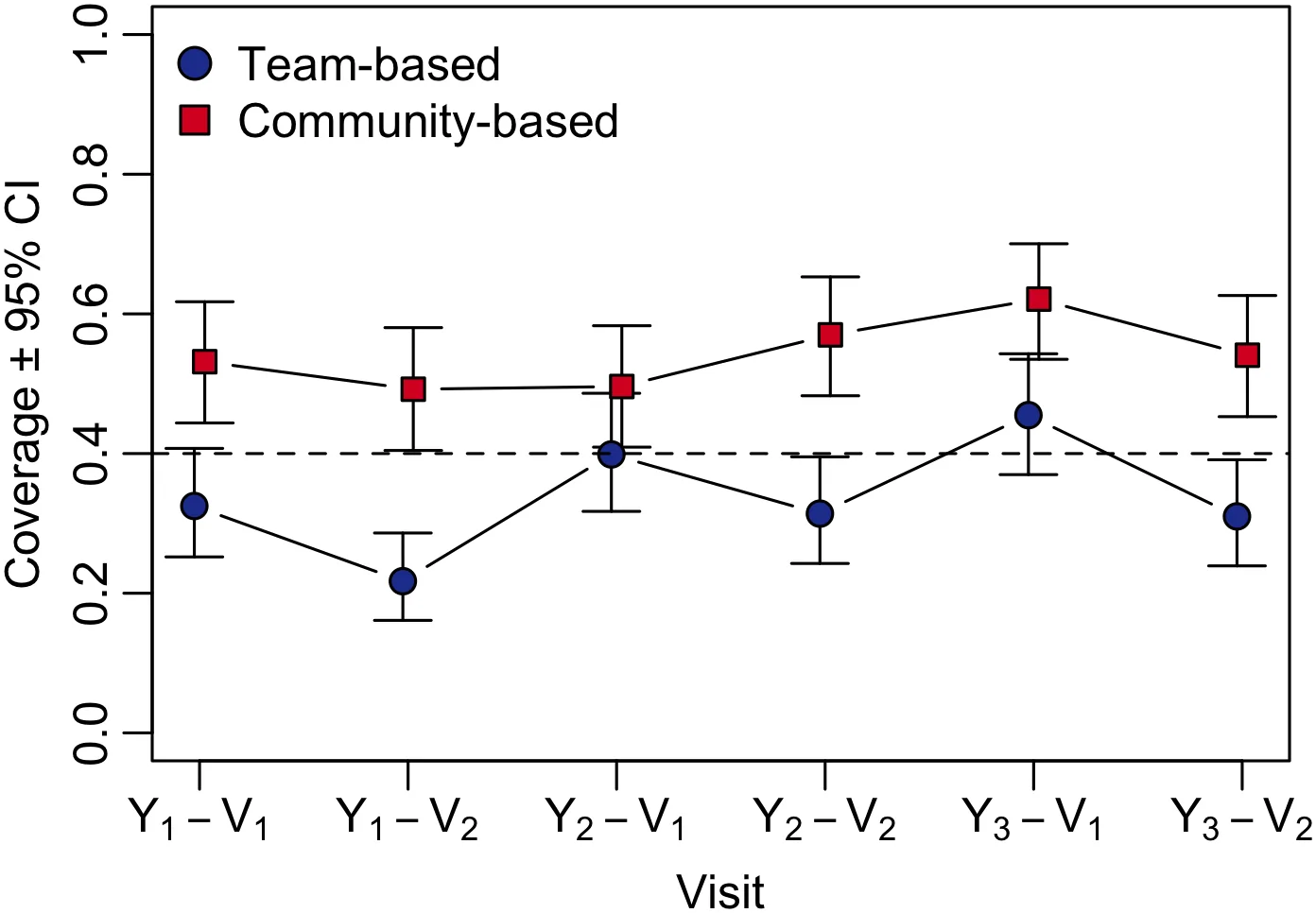
Estimated mean coverage ± 95% confidence limits at each survey time point, by trial arm. The critical threshold target of 40% is shown by a dashed line.

The odds ratio estimates for the intervention effect of the delivery strategies on coverage ranged from 1.48 to 3.49 and reflected significantly higher coverage in the Community-based arm at V_1_ and V_2_ across all three years of the trial (Fig 6). Detailed estimates from the primary analysis model are presented in S5 Table. When the primary analysis was repeated using the more stringent definition of coverage, the between-arm comparison gave similar results, with coverage across the six survey visits being higher in the Community-based arm (*p* < 0.001), although coverage estimates were lower (mean coverage 38% in the Community-based arm; mean 29% coverage in the Team-based arm) (S6 and S7 Tables; S3 and S4 Figs).

**Fig 6:**
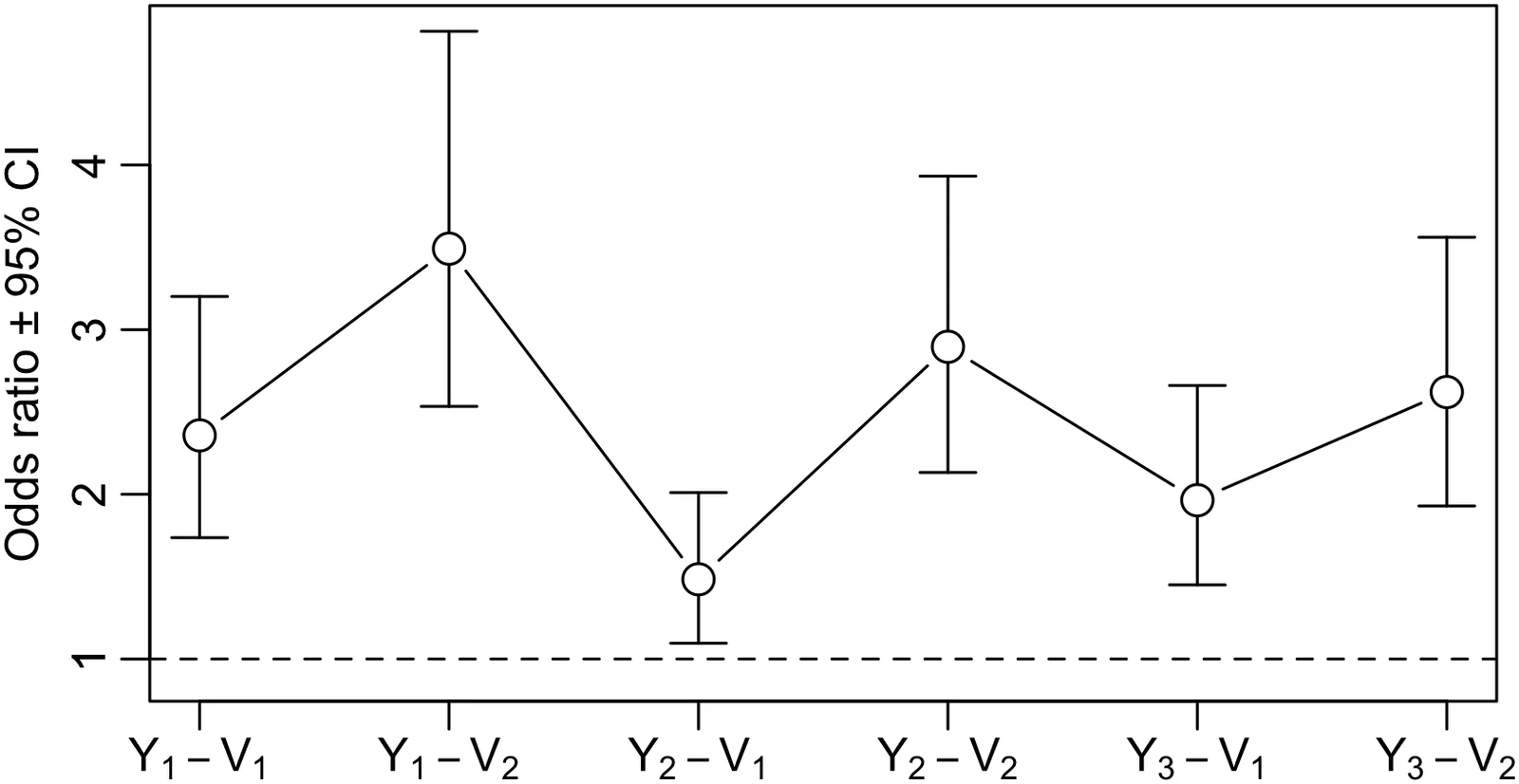
Estimated intervention effect (Community:Team) odds ratios ± 95% confidence limits at each survey time point. The null hypothesis of no intervention effect (odds ratio = 1) is shown by a dashed line.

### Secondary analyses

**Vaccination coverage at the second survey visit** (V_**2**_): To provide an indication of how coverage changed during the annual cycle, coverage was also measured at the end of each year in the V_2_ survey. Mean coverage differed between arms at V_2_ (*p* < 0.001; Table 2).

**The probability of coverage falling below 40%:** Fig 7 shows the probability distribution of coverage in each arm across months 1–12 of the vaccination cycle. At the start of the year, coverage is higher in the Community-based arm (month 1 mean: 57%) than the Team-based arm (month 1 mean: 46%). As the year progresses, the coverage distribution remains stable in the Community-based arm (month 12 mean: 53%) while it drops steadily in the Team-based arm (month 12 mean: 29%). As a consequence, the probability of a village falling below the 40% threshold in the Community-based arm is low and stable over time, from 20% in month 1–21% in month 12 (Table 4). By contrast, the probability of a village falling below the 40% threshold in the Team-based arm is substantially higher at 40% in month 1, increasing across the year to 80% of villages expected to fall below the critical threshold by month 12. Averaged across the trial year, the probability of coverage within a village dipping below the critical threshold was 18% (95% CI: 4, 40) in the Community-based arm and 60% (95% CI: 38, 81) in the Team-based arm.

**Table 4:** Estimated monthly probability (95% CI) of village-level coverage being below 40% and averaged over the three-year trial (Trial month), for each trial arm. The distribution of coverage among villages was estimated from 10,000 parametric bootstrap samples.

Trial month	Probability of coverage <40% (95% CI)	
	Team-based	Community-based
1	0.40 (0.19, 0.62)	0.20 (0.05, 0.41)
2	0.43 (0.21, 0.66)	0.19 (0.05, 0.40)
3	0.46 (0.23, 0.70)	0.19 (0.04, 0.40)
4	0.50 (0.26, 0.74)	0.18 (0.04, 0.40)
5	0.54 (0.30, 0.78)	0.17 (0.03, 0.40)
6	0.59 (0.33, 0.82)	0.17 (0.02, 0.40)
7	0.63 (0.38, 0.86)	0.17 (0.02, 0.40)
8	0.67 (0.42, 0.89)	0.17 (0.02, 0.40)
9	0.71 (0.46, 0.91)	0.18 (0.02, 0.41)
10	0.75 (0.51, 0.93)	0.18 (0.03, 0.42)
11	0.77 (0.54, 0.94)	0.20 (0.04, 0.43)
12	0.80 (0.57, 0.95)	0.21 (0.05, 0.45)
All months	0.60 (0.38, 0.81)	0.18 (0.04, 0.40)

**Fig 7:**
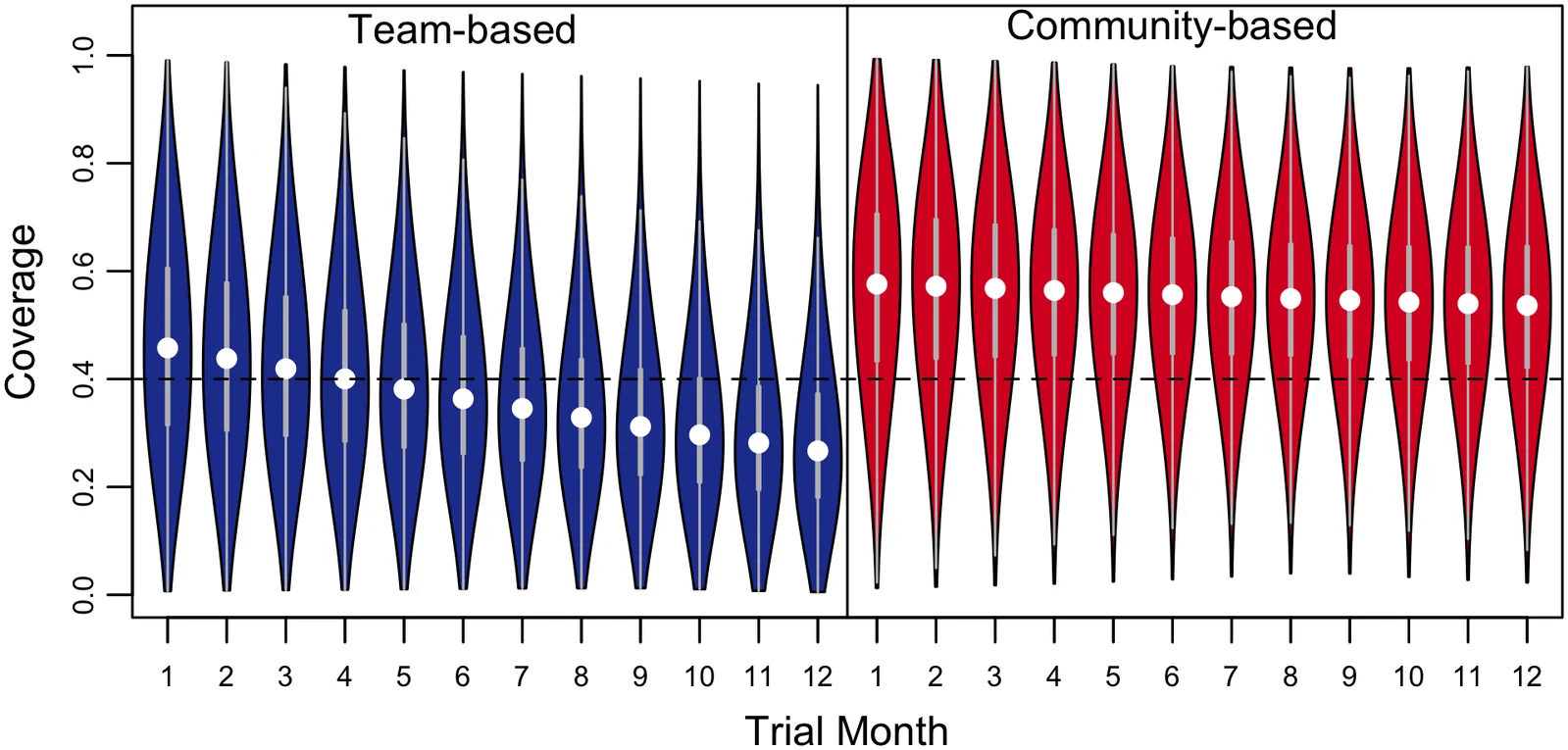
Estimated probability distribution of coverage by trial month for each arm. The critical threshold target of 40% is shown by a dashed line. White circles indicate medians and quartiles and range are shown by box-and-whisker plots.

Temporal variation in coverage:

(1) ***Consistency over time:*** Variation in coverage over the six survey visits, as quantified by the inter-survey odds ratio, was lower in the Community-based arm than the Team-based arm (Community:Team ratio: 0.67 [95% CI: 0.56, 0.83]). Mean coverage over the six timepoints was therefore significantly more consistent in the Community-based arm.
(2) ***Coverage change between visits:*** Coverage fell from V_1_ to V_2_ in the Team-based arm (odds ratio: 0.60 [95% CI: 0.50, 0.72]; *p* < 0.001), but not in the Community-based arm (odds ratio: 0.94 [95% CI: 0.79, 1.13]; *p* = 0.533). The V_2_:V_1_ odds ratio, which gauges change in coverage from V_2_ to V_1_, was 1.58-fold (95% CI: 1.30, 1.92) higher in the Community-based arm than the Team-based arm (arm × visit interaction *p* < 0.001). This interaction odds ratio can also be interpreted as the Community:Team odds ratio being 1.58 times higher at V_2_ than at V_1_. The arm × visit interaction effect did not differ between the three years (arm × visit × year interaction *p* = 0.256), justifying the estimation and testing of a single interaction effect rather than three year-specific effects.
(3) ***Minimum initial coverage:*** The start-of-year coverage target required to maintain coverage above the critical threshold (40%) by year-end was estimated using the rate of coverage decay in the Team-based delivery arm due to population turnover (dog mortality and unvaccinated births recorded). The target coverage required at the beginning of the year was 61% (95% CI: 53, 68) in Team-based and 42% (95% CI: 35, 50) in Community-based delivery, reflecting the relative stability of coverage over time in the Community-based arm.

**Spatial variation in coverage:** The Community:Team ratio of inter-ward variances was 2.21 (95% credible interval [CrI]: 0.09, 17.74); therefore there was no evidence for a difference between the trial arms in the amount of spatial variation in coverage. However, the wide 95% CrI indicates that there was insufficient power to detect even substantial differences (Table 5).

**Table 5:** Estimate (95% CI) of V_2_:V_1_ odds ratios and of minimum start-of-year coverage required for end-of-year coverage to >40%, by trial arm. The V_2_:V_1_ odds ratios differed significantly between trial arms (interaction *p* < 0.001).

Trial Arm	Visit effect OR (95% CI)	*p-*value	Year beginning coverage for year-end coverage ≥ 40%
Team-based	0.60 (0.50, 0.72)	<0.001	0.61 (0.53, 0.68)
Community-based	0.94 (0.79, 1.13)	0.533	0.42 (0.35, 0.50)

**Harms and unintended events in each group:** There were no harms, unintended events or adverse vaccination events reported to have occurred within each group.

## Discussion

Key findings from the RCT were that: i. vaccination coverage in the Community-based arm was consistently higher than that in the Team-based arm across all six timepoints and the effect was not lost when a more stringent definition of coverage was used; ii. Coverage distribution remained stable in the Community-based arm and exceeded 50% even at the end of the annual cycle, whilst coverage dropped steadily through the year in the Team-based arm and consequently there was a 60% probability of vaccination coverage in this arm being below the critical vaccination threshold (below which herd immunity is lost) at any point in an annual cycle; iii. Due to the tendency of coverage to decline through the year in Team-based delivery, whilst remaining relatively constant in Community-based delivery, initial coverage needs to be almost 50% higher in Team-based (61%) compared to Community-based (42%) delivery to ensure coverage does not dip below the critical threshold by the year end.

### Critical vaccination threshold

The importance of keeping vaccination coverage above the critical vaccination threshold is well understood. Impacted by the relatively low R_0_ of rabies, which in most settings is approximately 1.1 - 1.2, the critical vaccination coverage threshold has been estimated to be between 20% and 40% [11]. To keep coverage above the upper bound of this range (40%), the target coverage in an annual delivery approach has been estimated to be 70% [11]. Achieving this coverage at scale across rural landscapes where rabies remains endemic is logistically challenging. In this study, we used the vaccination decay rate, measured in the Team-based arm between the first and second survey point of three annual cycles, to estimate what initial vaccination coverage would need to be to ensure it did not drop below the critical threshold (40%). The resulting figure of 61% is lower than the 70% estimated previously but is 19% higher than the target needed to be reached at the start of each annual cycle through the Community-based approach [11].

### Benefits of increasing coverage

Although we have not reported impacts on rabies cases, achieving coverage greater than the critical vaccination coverage threshold is likely to be important in controlling transmission. Additionally, increasing coverage as far above the critical threshold as possible is likely advantageous for several reasons. First, as the number of immune individuals increases, so does the probable speed with which transmission events die out and rabies control can be achieved. Second, with more immune dogs and reduced transmission, the probability of a person being bitten will decline, resulting in a reduced probability of people being bitten, needing to urgently seek expensive PEP, and dying of rabies. In conclusion, for every additional percentage point of coverage achieved, numerous benefits of vaccination will probably accrue. Econometric analyses published separately showed that Community-based delivery had a lower cost per dog vaccinated than Team-based delivery across rural settings, but a higher cost per-dog vaccination in urban settings [30]. Given the demonstration that Community-based delivery achieved a coverage considerably higher than Team-based delivery, it seems likely that substantial and meaningful benefits can be realized through this method, in rural settings. However, analyses investigating the public health impact of Community-based delivery are required to fully understand the health benefits of the delivery approach. These analyses will be published separately.

### Community based delivery

The meaningful participation of communities in the co-design and delivery of interventions has normative benefits, and often leads to better outcomes, reduced costs, improved maintenance, efficiency, and enhanced social capital [40]. Indeed, in recent years implementing organizations have increasingly favored bottom-up approaches [41] involving local communities in the design and implementation of sustainable interventions, for example control programs targeting neglected tropical diseases such as onchocerciasis [42]. In the Community-based intervention tested in this RCT, communities were meaningfully involved at various levels: the unit of vaccination implementation was at the ward level, with coordination led by a ward-based Livestock Field Officer (the Rabies Coordinator) who was known to the community through existing government-sanctioned responsibility for oversight of all domestic animal health issues in the ward, including permission to administer vaccinations; a community leader (One Health Champion) coordinated vaccination activities within their respective village, with the expectation that they would harness their knowledge of dog-owning households and notify the Rabies Coordinators about owners and dogs that might have missed the initial clinic, or of litters recently born, allowing ad hoc vaccination of these dogs; vaccines were stored locally throughout the year which allowed local management and year-round use; monitoring and evaluation of vaccination outcomes by District Veterinary Officers, Rabies Coordinators and community leadership committees enabled vaccination to be reconfigured where necessary to suit needs [29]. Although implemented with a varying degree of fidelity across the target wards [29], these components aimed to embed the intervention within the community and, through the activities of the One Health Champions and their interaction with the Rabies Coordinators, to provide selected community members the opportunity to meaningfully manage rabies control in their own area through location-specific interventions. Indeed, that coverage achieved in the Community-based arm was more consistent than that achieved in the Team-based arm is logical, given that vaccines were available throughout the year. However, the finding that coverage was also significantly higher at the first time point of each year (Y_1-3_ V_1_) suggests that factors beyond the continuous availability of vaccines impacted the number of dogs that were vaccinated. For example, devolving responsibility to the One Health Champion to assist in the design, advertisement, scheduling and monitoring of the intervention likely had beneficial impacts that resulted in increased reach and effectiveness. The interplay between these factors will be investigated in subsequent analyses.

Finally, vaccination was provided free of charge in both arms of the trial and, whilst the Community-based approach was popular [29], we have no information regarding the level to which communities are willing and able to share the cost burden of these interventions and, consequently, the level to which external support will be required to maintain disease control activities at an effective level. The costs involved in the Community-based approach were mostly associated with personnel costs and costs associated with transport and communication. In the set up that was tested, Livestock Field Officers, a civil service paraprofessional cadre paid for by the state, took the role of the Rabies Coordinators, whilst the One Health Champions were ‘volunteers’ from the village leadership. Whether, going forward to sustain Community-based initiatives like this, such costs can be paid for by the Local Government Authority budgets or whether a proportion will need to be borne by the community itself remains to be seen. These questions are not limited to rabies control as the problem of program sustainability has broader relevance regarding how donor funded public health initiatives can be maintained and how local community involvement impacts sustainability and equity in outcomes. These questions are the focus of the next phase of this research.

### Thermotolerance

As previously demonstrated in other mass drug administrations, an essential feature of the Community-based dog vaccination was the availability of a thermotolerant vaccine that could be stored within local communities year-round. Although the official storage instructions given by the vaccine manufacturer continue to stipulate cold chain storage is required, the vaccine has been shown in repeated experimental and field-based trials to remain immunogenic following non-cold chain storage and this will likely be an important feature as rabies control efforts begin to target rural areas where vaccination has been challenging.

Thermotolerance and local storage in Zeepots provided multiple vaccination opportunities. This is important as it has been shown that, because of scheduling conflicts with activities such as market days and planting seasons, or because dogs sometimes cannot be caught on a specific day, dog owners frequently cannot attend clinics hosted on a single day each year [8]. Having vaccine continuously available will provide Rabies Coordinators and One Health Champions with improved agency to vaccinate at times that suits their needs.

### Impact of geography

The characteristics of a landscape will likely influence the effectiveness of mass vaccination delivery strategies. We demonstrated that Community-based dog vaccination achieves higher and more consistent coverage than the Team-based approach in northern Tanzania, a predominantly rural region dominated by small holder agro-pastoral communities with relatively small interspersed urban areas. The effectiveness of this approach may vary, however, in larger urban areas or pastoral settings, where differences in dog ownership patterns and human population densities could influence community vaccinators’ ability to reach dogs. Indeed, a cost comparison of these strategies revealed that the Community-based strategy had a lower cost per dog vaccinated than the Team-led strategy in rural settings (where over twice as many dogs are vaccinated), but the Team-led delivery had a lower cost per dog vaccinated in urban settings [30]. Achieving widespread rabies control across endemic countries such as Tanzania, composed of mosaics of agro-ecological areas and landscapes, comprising varied human and domestic animal densities, will require locally tailored context-dependent mass vaccination approaches that exploit different delivery strategies. Further, we caution against drawing conclusions regarding the suitability of the Community-based intervention in rabies endemic countries that do not share these agro-ecological similarities, for example in countries in Asia where landscapes and domestic dog demographies can be very different.

A related potential limitation of the trial arises from the imbalance in land use category proportions between arms, with 82% of Community-based wards being rural relative to 66% of Team-based wards. However, adjusting the primary analysis model for land use category did not improve fit significantly and had negligible impact on the intervention effect estimates (S8 Table), suggesting that variation in coverage between urban, semi-urban and rural wards did not bias the estimated intervention effect.

Furthermore, to examine the effectiveness of the implementation of the Community-based approach within the Tanzanian context and to evaluate the potential for successful normalization into routine vaccination practice of Tanzania, a process evaluation was implemented in parallel to this study [29]. The findings of this process evaluation were that implementers and community members understood the values and benefits of the Community-based approach, considered that it fitted well into routine schedules and context (infrastructure, skill sets and policy) and positively appraised it in terms of its perceived potential impact on rabies and recommended its use in Tanzania.

### Team-based arm

The coverage achieved in the Team-based arm was relatively modest (mean 37%). This contrasts with higher levels of coverage achieved through many team-based mass dog vaccination campaigns implemented in Africa and Asia where coverage in excess of a target of 70% has been achieved. Such programs are often managed by non-governmental organizations with relatively large budgets and experienced teams of implementers with the time and resources required to implement follow up transects, often guided by digital technologies that enable identification of geographic areas where coverage might be low triggering follow up vaccination efforts to improve coverage [43,44]. When employed in this manner, team-based approaches can be highly effective at reaching the target coverage of 70% required if coverage is not to decline to below the critical threshold of 40% before the team returns in a year’s time. However, to implement this approach effectively requires experience, resources and time. The objective of the reported study was to investigate how a team-based approach fared when implemented in a manner typical of national government teams that typically are instructed to target a new community each day using minimal resources. Under these circumstances the team-based approach fares less well.

### Limitations of certificates

Coverage was determined using vaccination certificates. There are several limitations to this method and these include: certificates can be lost, and dogs can be mistakenly assigned to a certificate; household might present certificates for dogs that have since died or been replaced; dogs that are stray or unowned are missed; certificates can be mistakenly issued without vaccination taking place (quality control failure). Alternative methods to identify vaccinated dogs are available, however, like certificates, all have limitations: microchipping dogs (expensive and painful and as such not always possible to administer), collars (often removed), mark-resight (time consuming as post-vaccination transects are required, these transects need to cover long distances to include the periphery of each village which tend to be large in the trial setting, and transects tend to overestimate coverage as puppies are frequently missed and are therefore not included in the denominator [8], and, as trialled by this group in another study [34], facial recognition (while sensitive and specific, internet connection is required). Given these factors, certificates were considered a reasonable method. Indeed, findings from an earlier study indicated that certificates were comparable to the gold standard microchip in the study setting [28].

In the sensitivity analysis which tested a more stringent measurement of vaccination (dogs reported as vaccinated but lacking a certificate were considered unvaccinated and were included in the denominator but not the numerator) the coverage was predictably lower in both arms (31.3% in the Community-based arm; 19.5% in the Team-based arm), however the overall difference in coverage between the two arms was similar.

## Conclusion

In summary, the Community-based approach investigated in this study has shown considerable potential to deliver high and consistent coverage in settings typical of many sub-Saharan African countries. Econometric analyses show that this approach is more cost-effective than the Team-led delivery in rural settings [30] and the consistently high coverage achieved suggests that it could be considered an important approach for national rabies elimination strategies (targeting similar agro-ecological settings) currently being designed in line with the global “Zero by 30” initiative and Gavi’s investments that encourage sustainable dog vaccination programs [45].

## Supporting information

S1 Checklist CONSORT 2010 Checklist. This checklist outlines the reporting items within each section for our randomized controlled trial as recommended by the CONSORT guidelines. For each item, a description is given and the specific location within the manuscript text, figures, or tables is provided. **No.:** Refers to the standard CONSORT checklist item number. **NA (Not Applicable):** Indicates items that do not apply to this trial design.
(DOCX)

S1 Protocol ISRCTN trial protocol registration record. The complete, validated trial registration protocol for the randomized controlled trial as submitted to the **ISRCTN registry** (Registration ID: **ISRCTN** 14813279). The record details the core protocol items required by the **World Health Organization (WHO)** and the **International Committee of Medical Journal Editors (ICMJE)** standards for clinical trial transparency, including the study design, participant eligibility criteria, interventions, and defined primary and secondary outcomes. The interactive and updated version of this record is publicly accessible at https://doi.org/10.1186/ISRCTN14813279.
(PDF)

S1 Table Description of Team-based dog mass vaccination using the TIDieR checklist. (DOCX)

S2 Table Description of Community-based mass dog vaccination using the TIDieR checklist. (DOCX)

S3 Table Summary of methods used for primary and secondary analyses. Generalized linear mixed-effects models (GLMMs) were fitted by maximum likelihood (ML) using the *glmmTMB* package and by Monte Carlo Markov-Chain (MCMC) using the *brms* package.
(DOCX)

S4 Table Moran’s test for spatial autocorrelation in the residuals of the primary analysis full model, applied at a range of spatial scales defined by the number of nearest neighbours (kNN). (DOCX)

S5 Table Estimates of fixed effects (log odds and log odds ratios) and random effects (variances) from the GLMMs fitted for the primary analysis. Numbers of observations, number of each random effect level, and marginal and conditional R^2^ are also presented. The null hypothesis of no intervention effect was rejected (χ^2^(6), *p* < 0.001).
(DOCX)

S6 Table Primary analysis (sensitivity analysis): Coverage and intervention odds ratio estimates (95% CI) at each survey estimated from the primary analysis GLMM re-fitted using a more stringent definition of coverage, where dogs claimed to be vaccinated but where no vaccination certificate could be produced were assumed to be unvaccinated (N dogs surveyed = 25,677). (DOCX)

S7 Table Primary analysis (sensitivity analysis): Estimates of fixed effects (log odds and log odds ratios) and random effects (variances) from the GLMMs fitted for the primary analysis. The primary analysis GLMMs were re-fitted using a more stringent definition of coverage, where dogs claimed to be vaccinated but where no vaccination certificate could be produced were assumed to be unvaccinated. Numbers of observations, number of each random effect level, and marginal and conditional R^2^ are also presented. The null hypothesis of no intervention effect was rejected (χ^2^(6), *p* < 0.001).
(DOCX)

S8 Table Primary analysis (sensitivity analysis): Estimates of fixed effects (log odds and log odds ratios) and random effects (variances) from the primary analysis GLMM and the same model adjusted for land use category. Number of observations, number of each random effect level, and marginal and conditional R^2^ are also presented. Adding land use category to the primary analysis model did not significantly improve fit (χ^2^(2), *p* = 0.175).
(DOCX)

S1 Fig Coverage by ward and survey time point in the Team-based arm. Point size is proportional to the number of dogs surveyed. District and ward names are given above each plot.
(TIF)

S2 Fig Coverage by ward and survey time point in the Community-based arm. Point size is proportional to the number of dogs surveyed. District and ward names are given above each plot.
(TIF)

S3 Fig Primary analysis (sensitivity analysis): Estimated coverage ± 95% confidence limits at each survey time point, by trial arm. A more stringent definition of coverage was used, where dogs claimed to be vaccinated but where no vaccination certificate could be produced were assumed to be unvaccinated. The critical threshold target of 40% is shown by a dashed line.
(TIF)

S4 Fig Primary analysis (sensitivity analysis): Estimated intervention effect (Community:Team) odds ratios ± 95% confidence limits at each survey time point. A more stringent definition of coverage was used, where dogs claimed to be vaccinated but where no vaccination certificate could be produced were assumed to be unvaccinated. The null hypothesis of no intervention effect (odds ratio = 1) is shown by a dashed line.
(TIF)
