# Supplementary material for: Effectiveness of community-based delivery of mass dog vaccination to prevent rabies: A cluster randomized controlled trial
Source: PLoS Negl Trop Dis. 2026 Sep 25;20(9):e0014704. doi: 10.1371/journal.pntd.0014704 (PMC13641700; doi:10.1371/journal.pntd.0014704)
Supplement: Supplementary file 1 [file pntd.0014704.s002.pdf]

ISRCTN14813279 https://doi.org/10.1186/ISRCTN14813279

Comparing two strategies for the mass vaccination of dogs to prevent rabies in humans

Submission date	Recruitment status	[X] Prospectively registered
01/04/2020	No longer recruiting	[X] Protocol
Registration date	Overall study status	[X] Statistical analysis plan
14/04/2020	Completed	[X] Results
Last Edited	Condition category	[_] Individual participant data
17/02/2025	Infections and Infestations	

Plain English summary of protocol

Background and study aims
Rabies is caused by an infection of nerve cells with the rabies virus. The virus travels through the nerves into the brain, where it causes brain inflammation and death in most cases if the person is not treated before symptoms appear. About 59,000 people are killed by rabies globally in each year. The majority of these deaths occur in Asia and Africa, where people are infected following bites from rabid (rabies-infected) dogs. Treating every person who has been bitten by a dog in case they have rabies is expensive and might not be available in time in rural communities. Mass dog vaccination (MDV) is a promising strategy, but it needs to be shown to reduce rabies infection in humans and to be practical to implement in remote areas. Previous rabies vaccines for dogs needed to be stored in a refrigerator, but newer versions can be stored at room temperature.
The standard method of MDV in Africa is to have a specialised team travel around with refrigerated vaccines and visiting each community to vaccinate dogs once a year. An alternative made possible by heat-stable vaccines is for people in each community to be trained to vaccinate dogs throughout the year. This study will compare the team-led and community-led methods in Tanzania, with both groups using a vaccine that can be stored at room temperature.

Who can participate?
All people living in the study areas in the Mara region of northern Tanzania will be potentially affected by the dog vaccination. Anybody living in the region who owns or looks after domestic dogs can volunteer to participate in the community-led delivery.

What does the study involve?
Administrative wards within the study area will be randomly allocated to receive the team-led or community-led vaccination. Dogs will have the rabies vaccination and will have a microchip inserted. 1 month and 11 months after the start of the vaccination campaign, 10 households per village at the centre of each study area will have their dogs checked for microchips, which will confirm whether they have been vaccinated.

What are the possible benefits and risks of participating?
The benefits of participating are that the village's dogs are vaccinated against rabies without charge. This will not only protect the dogs from rabies, but will also reduce the likelihood that

rabies will circulate in the community and that people will be bitten by rabid dogs. There are no obvious risk involved in taking part in the study.

Where is the study run from?
Washington State University (USA)

When is the study starting and how long is it expected to run for?
November 2018 to January 2024

Who is funding the study?
The National Institutes of Health (NIH) (USA)

Who is the main contact?
Professor Felix Lankester, felix.lankester@wsu.edu

Contact information

Type(s)
Public

Contact name
Prof Felix Lankester

ORCID ID
https://orcid.org/0000-0002-0802-0693

Contact details
Washington State University
240 SE Ott Road
Pullman
United States of America
99164-7090
+1 509.335.5861
felix.lankester@wsu.edu

Type(s)
Scientific

Contact name
Prof Felix Lankester

ORCID ID
https://orcid.org/0000-0002-0802-0693

Contact details
Washington State University
240 SE Ott Road
Pullman
United States of America

99164-7090
+1 509.335.5861
felix.lankester@wsu.edu

Additional identifiers

Clinical Trials Information System (CTIS)
Nil known

ClinicalTrials.gov (NCT)
Nil known

Protocol serial number
1R01AI141712-01

Study information

Scientific Title
Eliminating human rabies: impact of enhanced vaccination coverage

Acronym
T3

Study objectives
Rabies has the highest case fatality rate of any known human infectious disease and kills around 59,000 people annually. The vast majority (99%) of these fatalities occur in Africa and Asia due to canine rabies. While human rabies can be prevented with post-exposure prophylaxis (PEP), the intervention is expensive and often not available in the remote communities where it is most needed. Targeting control efforts at the reservoir host through mass dog vaccination (MDV) is a socially equitable and effective approach to eliminating human rabies. However, implementing MDV across the rural landscapes where rabies remains endemic is logistically challenging and, consequently, expensive. Moreover, there has only been limited empirical evidence to demonstrate the cost-effectiveness of MDV in achieving public health outcomes. As a result many countries spend substantial resources on provision of PEP with only limited investment in MDV and, without eliminating the transmission source, human rabies deaths continue. The standard method of delivering MDV across Africa is a centralized team-led delivery strategy. Based in central locations where power supplies allow vaccine storage in cold-chain conditions (4°C), teams drive to rural villages and set up temporary MDV clinics. To eliminate rabies on a regional scale, these once-per-year campaigns must vaccinate at least 70% of each community's dog population in order to maintain the minimum coverage above 20-45% (critical threshold - Pcrit) throughout the year. Otherwise natural turnover in the dog population leads to drops in coverage that allow sustained rabies transmission. Achieving this coverage level consistently across remote landscapes with team-led delivery, which is expensive and often results in a heterogeneous coverage, is challenging. Novel, cost-effective MDV delivery strategies that enable consistently high vaccination coverage to be achieved at the scale required for regional elimination are urgently needed. Decentralized community-led delivery strategies are a promising way of improving access to health interventions and have been used in Africa for the control of neglected tropical diseases such as onchocerciasis. In the case of rabies control, it has been hypothesized that moving towards a community-led model will improve consistency of coverage and reduce delivery costs. A key barrier to implementation of community-led

interventions has been the inability to store rabies vaccines under cold-chain conditions in resource-limited rural communities. However, the availability of a thermotolerant rabies vaccine, storable without loss of potency for extended periods at temperatures exceeding cold-chain conditions, would allow community-led delivery options to be explored. Our recent study investigating immunogenicity of a widely used canine vaccine (Nobivac™ Rabies) shows that immunogenicity to a protective level is not diminished following storage at 30°C for 3 months. This important outcome now enables novel decentralized delivery strategies to be implemented and tested.
Aim 1: Test the effectiveness of a decentralized community-led delivery strategy against the standard centralized team-led delivery via a randomized controlled trial (RCT). We will carry out a RCT to compare metrics of vaccination coverage under these two intervention strategies. We hypothesize that community-led delivery will result in more consistent coverage levels being achieved at lower cost per dose.
Aim 2: Compare the cost-effectiveness of the two delivery strategies.
In summary, we hypothesize that a community-led continuous mass dog rabies vaccination strategy will enable equivalent levels of vaccination coverage to be achieved when compared to a team-led pulsed mass dog rabies vaccination strategy at lower cost. The data generated will allow estimation and comparison of vaccination cost-effectiveness and the net benefits of public health outcomes under the two MDV delivery strategies. With a date of 2030 set by WHO/OIE/FAO for the global elimination of dog-mediated human rabies, the study's outputs will provide a critical contribution to guide elimination in canine-rabies endemic countries.

Ethics approval required
Old ethics approval format

Ethics approval(s)
1. Approved 04/12/2018, Ifakara Health Institute Review Board (IHI IRB, PO Box 78373, Dar Es Salaam, Tanzania; +255 (0) 22 2774714; irb@ihi.or.tz), ref: 24-2018
2. Approval extended 10/01/2020, Ifakara Health Institute Review Board (IHI IRB, PO Box 78373, Dar Es Salaam, Tanzania; +255 (0) 22 2774714; irb@ihi.or.tz), ref: IHI/IRB/EXT/01 - 2020

Study design
The study design will be a cluster randomised controlled trial (unblinded) in which the villages within 112 administrative wards will be assigned to receive mass dog vaccination through either of the two delivery strategies. The ward is the cluster.

Primary study design
Interventional

Study type(s)
Prevention

Health condition(s) or problem(s) studied
Canine-mediated rabies in humans

Interventions
We will test the efficacy of a novel mass dog rabies vaccination (MDRV) delivery strategy called community-led continuous delivery versus the existing team-led pulsed delivery strategy. To test this we will randomly assign (using a random allocation function in the programming language R) each of 112 administrative wards to receive either Arm 1 or Arm 2 delivery. The delivery of rabies vaccination will last 3 years.

Arm 1 - team-led delivery: Centralized delivery implemented by teams of vaccinators travelling from a central location (where vaccines are stored within refrigeration units) to each target community once a year
Arm 2 - community-led delivery: Decentralized delivery implemented by a locally based animal health para-professionals throughout the year using vaccines stored in low-tech cooling devices.

A cluster randomized trial design has been chosen to prevent contamination (spill-over of intervention effects) between the different arms of the trial. The trial will be clustered at the administrative ward level with each cluster being randomly selected into one of the two delivery strategies (Arm 1 or 2). Ward selection to each arm will be stratified at the district level, with an equal proportion of wards from each district being selected to the two arms of the trial. The chosen strategy will be delivered to all villages within the cluster. To avoid contamination between neighbouring clusters we will employ a 'fried-egg' approach, widely adopted in cluster randomized trials, in which the whole cluster receives the allocated treatment but only the inner area of the cluster ('egg-yolk') is used for surveillance since the treatment effect in this inner area will be less affected by spill-over from neighbouring clusters that may be in the opposite treatment arm. Using this approach we will only measure coverage in the ward’s central-most village (study village). Therefore the trial will be powered at the study village level.

To assess dog rabies vaccination coverage in both arms of the trial a random selection of households (10 per sub-village) within each study village will be visited and the dogs within the household restrained and scanned for the presence of a microchip. This will allow the proportion of vaccinated dogs in the village to be estimated. Given the proportion of stray dogs in these rural settings is typically low with most dogs having some form of ownership, we are confident that this method will provide an accurate estimate of coverage. This coverage assessment will be carried out twice: 1 month after the beginning of each annual mass dog vaccination campaign to determine immediate post-vaccination coverage, and 11 months after the mass dog vaccination campaign to determine population-immunity given demographic turnover. Because the interval between each team-led delivery pulse is 12 months, the 11-month time point has been selected to represent the period when coverage will be at its lowest and most likely to fall below the critical coverage threshold (Pcrit).

To assess cost-effectiveness, the fixed and variable costs associated with each delivery strategy will be recorded and a cost per dog vaccinated will be calculated.

Proposed outcome measures:
The primary outcome measure will be vaccination coverage. A dog will be considered vaccinated if, on post-vaccination coverage monitoring, it is found to have a microchip when scanned using a handheld digital scanner. Only dogs vaccinated in this study will have a microchip, as this technology is not otherwise used in Tanzania.
Aim 1.1 - The outcome measure will be mean vaccination coverage. That is the mean proportion of dogs in each village vaccinated against rabies (the mean of coverage at 1 and 11 months after annual campaigns. The hypothesis is that mean inter-campaign vaccination coverage achieved through community-led delivery will be higher than achieved through team-led delivery.
Aim 1.2 - The outcome measure will be vaccination coverage at the eleven-month time point within each annual vaccination cycle. The hypothesis is that the 11-month vaccination coverage in the team-led delivery arm will be lower than the coverage at the same time-point in the community-led delivery arm.
Aim 1.3 - The outcome measure will be variation in vaccination coverage over the 3-year time frame of the study. The hypothesis is that variation in coverage will be less in villages that receive community-led delivery.

Aim 1.4 - The outcome measure will be inter-village variation in vaccination coverage. The hypothesis is that inter-village variation in vaccination coverage will be lower following community-led delivery.

Aim 2: Compare costs of vaccine delivery by team- and community-led delivery
There are two hypotheses under this Aim: H1: The cost per dog vaccinated is higher under team-led than community-led delivery; H2: The marginal cost of coverage will be higher under team-led than community-led delivery.
The core cost-effectiveness (CE) analysis will compare the costs per vaccination C(i,s)/E(i,s), where E(i,s) in this case is the number of vaccinations delivered. ICERs, ΔC(I,s)/ΔE(i,s), will be calculated and tested for statistical significance, (Δ represents the difference in metrics across arms for each study site).

Intervention Type
Other

Primary outcome(s)
1. The proportion of dogs in each community (village) that have been vaccinated. A dog will be considered vaccinated if, on post-vaccination coverage monitoring, it is found to have a microchip when scanned using a handheld digital scanner. Only dogs vaccinated in this study will have a microchip, as this technology is not otherwise used in Tanzania.
2. Number of human dog-bite injuries assessed using hospital record data collected at district hospitals in the Mara region of Tanzania from October 2019 to August 2023
3. Number of human rabies cases assessed using hospital record data collected at district hospitals in the Mara region of Tanzania from October 2019 to August 2023

Key secondary outcome(s))
Cost of the delivery strategy per dog vaccinated

Completion date
31/01/2024

Eligibility

Key inclusion criteria
People who live in the target areas

Participant type(s)
All

Healthy volunteers allowed
No

Age group
All

Sex
All

Total final enrolment

198839

Key exclusion criteria
People who do not live or travel through the target areas

Date of first enrolment
01/05/2020

Date of final enrolment
30/08/2023

Locations

Countries of recruitment
Tanzania

Study participating centre
Bunda District Veterinary Office
Ministry of Livestock and Fisheries
Bunda
Tanzania
-

Study participating centre
Serengeti District Veterinary Office
-
Serengeti
Tanzania
-

Study participating centre
Tarime District Veterinary Office
-
Tarime
Tanzania
-

Study participating centre
Rorya District Veterinary Office
-
Rorya Town
Tanzania
-

Study participating centre
Butiama District Veterinary Office
-
Butiama
Tanzania
-

Sponsor information

Organisation
Washington State University

ROR
https://ror.org/01cwqze88

Funder(s)

Funder type
Government

Funder Name
National Institutes of Health

Alternative Name(s)
US National Institutes of Health, Institutos Nacionales de la Salud, NIH, USNIH

Funding Body Type
Government organisation

Funding Body Subtype
National government

Location
United States of America

Results and Publications

Individual participant data (IPD) sharing plan

The datasets generated and/or analysed during this study will be included in the subsequent results publication.

IPD sharing plan summary

Data sharing statement to be made available at a later date

Study outputs

Output type	Details	Date created	Date added	Peer reviewed?	Patient-facing?
Participant information sheet	Participant information sheet	11/11/2025	11/11/2025	No	Yes
Preprint results		31/10/2024	17/02/2025	No	No
Protocol file			10/04/2024	No	No
Statistical Analysis Plan	version 1.1	24/04/2024	25/04/2024	No	No
