# Supplementary material for: Effectiveness of community-based delivery of mass dog vaccination to prevent rabies: A cluster randomized controlled trial
Source: PLoS Negl Trop Dis. 2026 Sep 25;20(9):e0014704. doi: 10.1371/journal.pntd.0014704 (PMC13641700; doi:10.1371/journal.pntd.0014704)
Supplement: Supplementary file 3 [file pntd.0014704.s003.docx]

**S1 Table :** Description of a Team-based dog vaccination using the TIDieR checklist

| Item | Intervention description |
| --- | --- |
| Brief name of intervention | Team-based mass dog vaccination (TB-MDV) approach |
| Why the intervention | The TB-MDV was employed to serve as a standard benchmark to compare the effectiveness , coverage, and cost-effectiveness of the novel CB-MDV strategy. The use of the standard dog vaccination procedure in the control arm allows for an evaluation of the relative benefits and potential improvements offered by the new approach. |
| What materials were used in the delivery of the intervention | Nobivac Rabies vaccine and vaccination consumables (syringes, needles, gloves, etc.) and cold boxes for carrying vaccines and trunks for storing vaccination materials;  Smartphones with data-capturing applications installed;  Vaccination cards/certificates, vaccination registers;  Megaphones to support advertising of campaigns;  Gumboots and raincoats;  Refrigerator for storing the vaccines at the district headquarters;  An implementation manual to guide delivery;  All vaccination equipment kept at the district level. |
| Procedures, activities or processes used | The team-based dog vaccination strategy involved forming a vaccination team composed of a Senior Livestock Field Officer from the district headquarters and a ward-based Livestock Field Officer. Each village also designated a local assistant to assist with registering vaccinated dogs in the official vaccination register and managing the orderly flow of owners and animals at the vaccination site. The team implemented all vaccination activities within a ward, after which the Senior Livestock Field Officer moved to the next ward and formed a new team with the respective ward-based Livestock Field Officer. Each new village again provided a local assistant to support the process.  Dog vaccination was conducted through static-point clinics set up at central and easily accessible village locations. At the clinic, the Senior Livestock Field Officer registered each dog in the smartphone application and administered a subcutaneous 1-mL dose of Nobivac Rabies vaccine. The ward-based Livestock Field Officer verified and certified each vaccinated dog, while the local assistant recorded details in the vaccination register.  Prior to each clinic, community leaders conducted sensitization by disseminating information through word of mouth and posting notices in prominent public areas. Vaccination campaigns were executed according to predetermined schedules, with the Senior Livestock Field Officer traveling between villages, carrying vaccination equipment, and overseeing the logistics and implementation of each campaign. |
| Who provided the intervention | District Veterinary Officers managed vaccine stocks, vaccination materials, and overall supervision of campaigns within their districts. The Senior Livestock Field Officer was responsible for registering dogs in the smartphone application and administering a subcutaneous 1-mL dose of Nobivac Rabies vaccine.  The ward-based Livestock Field Officer verified and certified each vaccinated dog by completing the owner’s vaccination certificate, while the local assistant recorded vaccination details in the register, including the dog’s name, sex, age, color, and the owner’s name.  All vaccination data were entered into a digital data collection tool by the Senior Livestock Field Officer. |
| What was the mode of delivery of the intervention | The intervention was delivered by teams of vaccinators led by the Senior Livestock Field Officer who would travel to the ward and the target village from the district headquarter bringing the required equipment including cold-chain stored vaccines.  Once at the village, a static-point clinic was set up in a central location within each village. Dog owners were instructed to bring their dogs to this clinic. On arrival, the vaccination team registered dogs and owners, administered the rabies vaccine, and recorded vaccination data. The clinics operated for the full day at each location.  Monitoring of vaccination activities was done by District Veterinary Officers through electronically uploaded data. |
| Where the intervention was delivered | TB-MDV was delivered in randomly selected wards (n=56) in all six administrative districts of the Mara region of the Lake Zone of north-west Tanzania. |
| When and how often was the intervention was delivered | The intervention was delivered annually in three cycles, starting in November each year. Each village hosted a single static-point clinic within the first month of the cycle. The Senior Livestock Field Officers moved from village to village, vaccinating all assigned villages within a ward before proceeding to the next ward. This process was repeated annually for three years, ensuring that each ward received dog vaccination once per year. |
| Aspects of the intervention that were subject to tailoring | The Senior Livestock Field Officers tailored advertisement campaigns to align with existing administrative structures. For example, community leaders received letters informing them of upcoming vaccination campaigns a week in advance. Additionally, advertisements were placed in prominent areas. |
| How the intervention was modified in delivery | N/A |
| Planned: How intervention adherence or fidelity was assessed and by whom | TB-MDV is the standard intervention and as such fidelity was not assessed. |
| Actual: extent to which intervention was delivered as planned | N/A |
