# Supplementary material for: Effectiveness of community-based delivery of mass dog vaccination to prevent rabies: A cluster randomized controlled trial
Source: PLoS Negl Trop Dis. 2026 Sep 25;20(9):e0014704. doi: 10.1371/journal.pntd.0014704 (PMC13641700; doi:10.1371/journal.pntd.0014704)
Supplement: Supplementary file 4 [file pntd.0014704.s004.docx]

**S2 Table :** Description of Community-based mass dog vaccination using the TIDieR checklist

| Item | Intervention description |
| --- | --- |
| Brief name of intervention | Community-based mass dog vaccination (CB-MDV) approach |
| Why the intervention | CB-MDV was designed to ensure continuous access to mass dog vaccination by decentralizing vaccine storage to the ward level. This approach facilitated year-round vaccination availability. The strategy aims to ensure that herd immunity is maintained among the dog population all year around. |
| What materials were used in the delivery of the intervention | Nobivac Rabies vaccine and vaccination consumables (syringes, needles, gloves, etc.) and cold boxes for carrying vaccines and trunks for storing vaccination materials;  Smartphones with data-capturing applications installed;  Vaccination cards/certificates, vaccination registers;  Megaphones to support advertising of campaigns;  Gumboots and raincoats;  Zeepot for storing the vaccines in the wards, and temperature data loggers to monitor the internal temperature of the device;  Branded caps and T-shirts;  An implementation manual to guide the delivery  All vaccination equipment is kept at the ward level. |
| Procedures, activities or processes used | Key intervention procedures included assigning District Veterinary Officers or District Livestock Field Officers to coordinate vaccination activities at the district level. At the ward level, Livestock Field Officers served as Rabies Coordinators. A community leader (One Health Champion) was chosen from each village to assist the Rabies Coordinators during vaccination activities, including estimating dog populations, identifying unvaccinated dogs, and assisting with advertising and vaccinations.  Community leadership committees were consulted regarding planning of rabies vaccination activities. Approximately 7–10 days before clinics, Rabies Coordinators and One Health Champions attended village meetings to explain the mass dog vaccination approach. Vaccines were stored in Zeepots by Rabies Coordinators in the ward.  Vaccination campaigns were free of charge.  Vaccination pulses were organized at the discretion of Rabies Coordinators. Additionally, dog owners were able to request vaccinations on demand. Unused vaccines were returned by the Rabies Coordinators to the District Veterinary Officer’s office every six months for disposal.  Rabies Coordinators were trained on how to manage vaccine collection and storage. This included instruction regarding the collection of vaccine batches from the central storage at the respective District Veterinary Office, storage of vaccines within their respective wards using the low-tech cooling system (Zeepots), and organization and management of vaccination campaigns in each of the villages of the ward.  Additionally, the One Health Champions were instructed on how to estimate the dog population in their respective villages and to sensitize the community regarding the requirement for rabies vaccination and upcoming clinics. |
| Who provided the intervention | District Veterinary Officers managed vaccine stocks, provided supervision, and oversaw vaccination campaigns within their respective districts.  Rabies Coordinators, based at the ward level, implemented vaccination activities across all villages in their wards, including registration, vaccination, and coordination of logistics.  One Health Champions supported the Rabies Coordinators by engaging communities, advertising vaccination activities, and assisting with the organization of clinics within their respective villages.  At the clinics, the Rabies Coordinators registered dogs in the smartphone application and administered a subcutaneous 1-mL dose of Nobivac Rabies vaccine. The One Health Champions assisted in filling out vaccination certificates for dog owners and recording vaccination details in the official register, including each dog’s name, sex, age, color, and the owner’s name.  All registration data were entered into a digital data collection survey tool by the Rabies Coordinators. |
| What was the mode of delivery of the intervention | Community engagement and sensitization were conducted through face-to-face interactions during village meetings. Vaccination campaigns were advertised using megaphone announcements, public gatherings, and notices posted at strategic locations such as village government offices.  The dog vaccinations were delivered by Rabies Coordinators and One Health Championsthrough either i) static point clinics in which dog owners attended a vaccination clinic, ii) house-to-house vaccination in which dogs were vaccinated within the household, iii) or dog owners were able to request vaccinations on demand.  Monitoring of vaccination activities was done through data electronically uploaded via a smartphone dog registration app. |
| Where the intervention was delivered | CB-MDV was delivered in randomly selected wards (n=56) in all six administrative districts of the Mara region of the Lake Zone of north-west Tanzania. |
| When and how often was the intervention was delivered | A village level static point clinic was hosted at the start of the annual cycle. Thereafter, further vaccination throughout the year was implemented either through additional static point clinics at the village or sub-village level or through house-to-house visits to locate dogs. Additionally, throughout the year, dog owners could specifically ask for their dogs to be vaccinated which would be implemented upon request. |
| Aspects of the intervention that were subject to tailoring | The Rabies Coordinators tailored vaccination schedules and delivery methods to align with their other work commitments, the number of dogs needing vaccination, and village sizes. For instance, they could choose house-to-house vaccination if it proved more efficient based on the estimate of the number of unvaccinated dogs. |
| How the intervention was modified in delivery | The protocol was modified as follows:  Due to periods of no vaccination occuring, the District Veterinary Officers set monthly targets for the number of dogs that they expected to be vaccinated which were based upon village dog population estimates. This encouraged Rabies Coordinators to continue to vaccinate through each annual cycle. |
| Planned: How intervention adherence or fidelity was assessed and by whom | The implementation process was guided by a manual, and fidelity was measured by an implementation scientist as part of a process evaluation through which data, collected using non-participant observations, meeting reports, implementation audits and in-depth interviews, was analyzed thematically and descriptively. |
| Actual: extent to which intervention was delivered as planned | The fidelity of the CB-MDV was determined to be greater than 80%. |
