# Supplementary material for: Effectiveness of community-based delivery of mass dog vaccination to prevent rabies: A cluster randomized controlled trial
Source: PLoS Negl Trop Dis. 2026 Sep 25;20(9):e0014704. doi: 10.1371/journal.pntd.0014704 (PMC13641700; doi:10.1371/journal.pntd.0014704)
Supplement: Supplementary file 5 [file pntd.0014704.s005.docx]

**S3 Table .** Summary of methods used for primary and secondary analyses. Generalized linear mixed-effects models (GLMMs) were fitted by maximum likelihood (ML) using the *glmmTMB* package and by Monte Carlo Markov-Chain (MCMC) using the *brms* package.

| Analysis level | Question | GLMM fixed effects | GLMM random effects (number of categories) | GLMM fitting method | Estimates |
| --- | --- | --- | --- | --- | --- |
| Primary | Does mean coverage differ between arms at any of the six time-points? | arm + visit + year +  arm × visit +  arm × year +  visit × year +  arm × visit × year | District (9),  ward (112),  sub-village (659);  visit-specific: district (54), ward (663); sub-village (2376); household (12999) | ML | Odds ratio estimate & Wald 95% CI at each survey visit in each year; LRT *p*-value for null hypothesis that each OR = 1. |
| Secondary | Does coverage differ between arms at any of the three survey visit 2 time-points? | Same as Primary | Same as Primary | ML | Odds ratio estimate & Wald 95% CI at survey visit 2 in each year (already calculated for Primary); LRT *p*-value for null hypothesis that each OR = 1. |
| Secondary | What is the probability of coverage in a ward being below 40% in each arm, per trial month and averaged over the year. | arm + month + year +  arm × month +  arm × year | Same as Primary | ML | Distribution of predicted coverage by arm and month estimated by simulating new wards, incorporating model uncertainty via 10,000 parametric bootstrap samples; P(coverage < 40%) by arm and month & 95% CI |
| Secondary | Does the consistency of coverage over the duration of the trial differ between arms? | Same as Primary | Same as Primary | ML | Mean absolute difference between 6 consecutive time-points in log odds of predicted coverage exponentiated to an odds ratio, for each arm; the Community:Team ratio of odds ratios; 95% CI for each ratio, estimated using 10,000 parametric bootstrap samples |
| Secondary | Does the rate of change in coverage from survey visit 1 to survey visit 2 differ between arms? | arm + visit + year +  arm × visit +  arm × year | Same as Primary | ML | arm × visit interaction odds ratio, Wald 95% CI and Wald *p*-value; Survey Visit effect odds ratio, Wald 95% CI and Wald *p*-value, for each arm. |
| Secondary | Given the observed rate of change of coverage within a year, what is the minimum coverage required at survey visit 1 to ensure that coverage at survey visit 2 does not drop below the critical vaccination threshold? | Same as the model above | Same as Primary | ML | Coverage at survey visit 1 predicted to give 40% coverage at survey visit 2 & Wald 95% CI, calculated using the odds ratio estimates from the model above. |
| Secondary | Does the consistency of coverage over space (i.e. between wards) differ between arms? | Same as Primary | Same as Primary, except that the inter-ward random effect variance varies between arms | MCMC | Median and 95% CI from the posterior distribution of the Community:Team ratio of inter-ward variances |
