# Supplementary material for: Effectiveness of community-based delivery of mass dog vaccination to prevent rabies: A cluster randomized controlled trial
Source: PLoS Negl Trop Dis. 2026 Sep 25;20(9):e0014704. doi: 10.1371/journal.pntd.0014704 (PMC13641700; doi:10.1371/journal.pntd.0014704)
Supplement: Supplementary file 6 [file pntd.0014704.s006.docx]

| **S4 Table .**  Moran's test for spatial autocorrelation in the residuals of the primary analysis full model, applied at a range of spatial scales defined by the number of nearest neighbours (kNN). | | |
| --- | --- | --- |
| kNN | Moran's *I* | *p*-value |
| 1  2  3  4  5  6  7  8  9  10 | 1.24  1.26  0.54  0.33  -0.14  -0.19  -0.11  0.14  0.59  0.72 | 0.108  0.104  0.295  0.372  0.556  0.576  0.546  0.445  0.277  0.237 |
