# Supplementary material for: Effectiveness of community-based delivery of mass dog vaccination to prevent rabies: A cluster randomized controlled trial
Source: PLoS Negl Trop Dis. 2026 Sep 25;20(9):e0014704. doi: 10.1371/journal.pntd.0014704 (PMC13641700; doi:10.1371/journal.pntd.0014704)
Supplement: Supplementary file 8 [file pntd.0014704.s008.docx]

| **S6 Table .**  Primary analysis (sensitivity analysis): Coverage and intervention odds ratio estimates (95% CI) at each survey estimated from the primary analysis GLMM re-fitted using a more stringent definition of coverage, where dogs claimed to be vaccinated but where no vaccination certificate could be produced were assumed to be unvaccinated (N dogs surveyed = 25,677). | | | | |
| --- | --- | --- | --- | --- |
| Year-visit | Coverage % (95% CI) | | Odds ratio (95% CI) | *p*-value |
|  | Team-based | Community-based |  |  |
| Y_1_-V_1_ Y_1_-V_2_ Y_2_-V_1_ Y_2_-V_2_ Y_3_-V_1_ Y_3_-V_2_ | 26 (20, 33) 17 (13, 23) 30 (23, 38) 24 (18, 31) 34 (27, 42) 23 (17, 29) | 37 (30, 46) 34 (26, 42) 34 (27, 42) 41 (33, 49) 42 (34, 50) 36 (28, 44) | 1.72 (1.32, 2.23) 2.40 (1.83, 3.16) 1.20 (0.92, 1.55) 2.14 (1.65, 2.77) 1.38 (1.07, 1.79) 1.89 (1.45, 2.46) | <0.001 <0.001 0.174 <0.001 0.013 <0.001 |
| All years | 29 (22, 37) | 38 (30, 47) | 1.53 (1.22, 1.93) | 0.001 |
