# Supplementary material for: Effectiveness of community-based delivery of mass dog vaccination to prevent rabies: A cluster randomized controlled trial
Source: PLoS Negl Trop Dis. 2026 Sep 25;20(9):e0014704. doi: 10.1371/journal.pntd.0014704 (PMC13641700; doi:10.1371/journal.pntd.0014704)
Supplement: Supplementary file 9 [file pntd.0014704.s009.docx]

| **S7 Table** . Primary analysis (sensitivity analysis): Estimates of fixed effects (log odds and log odds ratios) and random effects (variances) from the GLMMs fitted for the primary analysis. The primary analysis GLMMs were re-fitted using a more stringent definition of coverage, where dogs claimed to be vaccinated but where no vaccination certificate could be produced were assumed to be unvaccinated. Numbers of observations, number of each random effect level, and marginal and conditional R^2^ are also presented. The null hypothesis of no intervention effect was rejected (χ^2^(6), *p* < 0.001). | | | | | | | | |
| --- | --- | --- | --- | --- | --- | --- | --- | --- |
|  | *Primary analysis null hypothesis model* | | | | *Primary analysis alternative hypothesis model* | | | |
| *Predictors* | *Log-Odds* | *std. Error* | *CI* | *p* | *Log-Odds* | *std. Error* | *CI* | *p* |
| (Intercept) | -0.98 | 0.21 | -1.39, -0.58 | <0.001 | -1.32 | 0.22 | -1.76, -0.89 | <0.001 |
| Visit: V_2_ | -0.39 | 0.20 | -0.77, -0.00 | 0.048 | -0.61 | 0.22 | -1.05, -0.17 | 0.007 |
| Year: Y_2_ | 0.03 | 0.19 | -0.35, 0.41 | 0.872 | 0.27 | 0.22 | -0.16, 0.70 | 0.220 |
| Year: Y_3_ | 0.36 | 0.19 | -0.02, 0.74 | 0.061 | 0.50 | 0.22 | 0.08, 0.93 | 0.021 |
| VisitV_2_:YearY_2_ | 0.40 | 0.28 | -0.14, 0.94 | 0.147 | 0.24 | 0.31 | -0.38, 0.85 | 0.450 |
| VisitV_2_:YearY_3_ | -0.12 | 0.28 | -0.66, 0.42 | 0.673 | -0.10 | 0.31 | -0.71, 0.52 | 0.756 |
| Trial Arm: Community-based |  |  |  |  | 0.67 | 0.17 | 0.35, 0.99 | <0.001 |
| VisitV_2_:Trial_ArmCommunity-based |  |  |  |  | 0.42 | 0.20 | 0.02, 0.81 | 0.038 |
| YearY_2_:Trial_ArmCommunity-based |  |  |  |  | -0.45 | 0.19 | -0.83, -0.07 | 0.021 |
| YearY_3_:Trial_ArmCommunity-based |  |  |  |  | -0.27 | 0.19 | -0.64, 0.11 | 0.165 |
| VisitV_2_:YearY_2_:Trial_ArmCommunity-based |  |  |  |  | 0.30 | 0.28 | -0.24, 0.85 | 0.277 |
| VisitV_2_:YearY_3_:Trial_ArmCommunity-based |  |  |  |  | -0.03 | 0.28 | -0.57, 0.52 | 0.917 |
| **Random Effects** | | | | | | | | |
| σ^2^ | 3.29 | | | | 3.29 | | | |
| τ_00_ | 0.55 _sub_village.rnd_ | | | | 0.55 _sub_village.rnd_ | | | |
|  | 0.46 _sub_village_ | | | | 0.48 _sub_village_ | | | |
|  | 0.15 _ward.rnd_ | | | | 0.11 _ward.rnd_ | | | |
|  | 0.24 _ward_ | | | | 0.13 _ward_ | | | |
|  | 0.12 _district.rnd_ | | | | 0.13 _district.rnd_ | | | |
|  | 0.18 _district_ | | | | 0.18 _district_ | | | |
| N | 2473 _sub_village.rnd_ | | | | 2473 _sub_village.rnd_ | | | |
|  | 687 _sub_village_ | | | | 687 _sub_village_ | | | |
|  | 663 _ward.rnd_ | | | | 663 _ward.rnd_ | | | |
|  | 112 _ward_ | | | | 112 _ward_ | | | |
|  | 54 _district.rnd_ | | | | 54 _district.rnd_ | | | |
|  | 9 _district_ | | | | 9 _district_ | | | |
| Observations | 25677 | | | | 25677 | | | |
| Marginal R^2^ / Conditional R^2^ | 0.010 / 0.347 | | | | 0.038 / 0.350 | | | |
