# Supplementary material for: Effectiveness of community-based delivery of mass dog vaccination to prevent rabies: A cluster randomized controlled trial
Source: PLoS Negl Trop Dis. 2026 Sep 25;20(9):e0014704. doi: 10.1371/journal.pntd.0014704 (PMC13641700; doi:10.1371/journal.pntd.0014704)
Supplement: Supplementary file 11 [file pntd.0014704.s015.docx]

**Response to Reviewers:**

The co-authors would like to thank the two reviewers for their time reviewing our manuscript and for their constructive reviews. The comments were extremely helpful and, in addressing them all (below), have allowed us to improve the manuscript considerably.

Two major concerns were identified:

1. ***Summary of concern:*** *The allocation of resources to each arm were not identified and were likely different and that this variable allocation meant that the community-based arm likely had several advantages over the teams-based approach and that these were not accounted for in the analysis.*

**Response:** This is an interesting point and gets to the nub of what this RCT was attempting to achieve. The paper describes a pragmatic RCT in which different delivery strategies were pursued, allowing for resource allocation decisions and tactical delivery decisions made by providers to differ within overall strategic approaches, but subject to comparable overall resource constraints. In such trials, one of the most fundamental objectives is to evaluate an intervention as it would be delivered in the real world*.* This is the primary objective of this paper.

With this framing of both the trial and research question, resource allocation differences between arms (subject to comparable overall resource constraints) are not a confounder but are part of the intervention package. For example, if effective delivery via one vaccination strategy inherently calls for different staff activities or vehicle resources to work effectively for the specific delivery strategy, then restricting resource allocation within the delivery design would limit both its potential effectiveness and effectiveness as delivered. This logic is common in complex health interventions, where the Medical Research Council (MRC) framework (Skivington 2021, BMC Research Methods and Reporting; <https://www.bmj.com/content/374/bmj.n2061>) explicitly recognises that interventions are complex activities that must be responsive to the environment in which they are being implemented, and especially within the context of the fundamental delivery strategy and resource constraints imposed. By design, the delivery strategies differed across arms, but within each delivery strategy context, healthcare providers were making resource allocation decisions subject to these basic strategic and comparable overall resource constraints.

This allowance for different tactical responses to different strategies matters a lot in trials like this. For example, those comparing vaccination delivery. If one arm uses fixed-point delivery and the other uses roving teams, those arms will inherently differ in resource allocations, staff time, and community engagement. If the finer tactical differences of delivery were constrained, it would reduce the effectiveness of one or the other (or both) strategies beyond the intent of this comparison, most likely in favor of one or the other strategy. Most published trials in this area do vary operationally between arms, and this is generally acknowledged. Readers are expected to interpret results with this caveat in mind.

Having said this, trials should at minimum clearly specify whether the intent is efficacy (controlled conditions) or effectiveness (real-world conditions), justify any resource differences, and conduct economic analyses in parallel to the trial so that cost differences are quantified rather than buried.

With this in mind, we have edited the text to make it more explicit that the reported RCT was testing the real-world effectiveness (and not the efficacy) of two different strategies, and that as a result the strategies did experience variation in operational resources. We have explained the resource allocation to each arm, with new reference to a pilot study that informed these resource allocations, and, where appropriate, ‘effectiveness’ has replaced ‘efficacy’ throughout, and text explaining this approach is in (and has been added to) the Introduction (Lines 146-70) and Methods (Lines 227-62). Additionally, we have clarified that the objective of this paper was to report the different levels of coverage achieved by the two strategies. We have also clarified that measures of cost were collected and these will be reported, together with cost-effectiveness analyses, in separate papers.

2. ***Summary of concern:*** *The pulsed team-based approach did not achieve coverage levels comparable to other team-based interventions reported in the literature.*

**Response:** This is an important point as it is important to distinguish between team-based approaches delivered by, for example, national governments and those delivered by non-governmental organizations that typically have more resources (being donor funded). Because we wanted to interrogate a delivery strategy that might be employed at scale in national elimination campaigns, the team-based approach employed in the study is representative of the type of approach used when mass dog vaccination is managed by national governments. This approach tends to involve, for example, a single day of vaccination per village / community. Following this approach, vaccination coverage tends not to be as high as that achieved by mass dog vaccination events hosted by non-governmental organizations.

Text clarifying this has been added (Line 234-40, 265-304 and 751-65).

A response to all reviewer comments has been given below:

*Reviewer #1:*

***Comment:*** *Objectives are clearly articulated - comparing effectiveness of community based continuous delivery of mass dog vaccination with standard team-based delivery. The hypothesis that coverage differs between delivery strategies is clear and testable. The study is appropriate to address the stated objectives - the 'fried egg' design to reduce contamination is thoughtful. Greater clarity would be good on how representative the team based approach of 'standard practice' is given the modest post-vaccination campaign estimates are. They seem very modest for standard campaigns of this nature which are typically higher in sub saharan Africa.*

***Response:*** The team-based approach employed in the study is representative of the typical approach used when mass dog vaccination is managed by national governments and ministries responsible for animal health, where campaigns are constrained by limited funding, transport and personnel. As a result this approach tends to involve a single day of vaccination per village / community. Following this approach, vaccination coverage tends not to be as high as that achieved by mass dog vaccination events hosted by non-governmental organizations which typically have more resources. This distinction has been made on Line 234-40, 265-304 and 751-65.

***Comment:*** *The population is clearly described and appropriate for the hypothesis being tested in endemic rural African settings. Generalisation beyond this context should be framed cautiously i.e. scaling for larger urban areas or cross border transmission networks would require further evaluation.*

**Response:** This is a pertinent point, and we have clarified this on Lines 730-740 and

***Comment:*** *The sample size (56 ward arms) appears sufficient for detecting differences in coverage and the power calculations are clearly described. The trial is large enough and statistically robust in coverage - but it is not powered to assess endpoints such as dog rabies incidence or human exposure trends. This doesn't weaken the stated objective of the study - but it possibly limits conclusions about elimination impact. The statistical analysis looks rigours and appropriate. Additional clarity around assumptions used in estimating 'minimum initial coverage' and probably of falling below 40% would be of added benefit.*

**Response:** The critical vaccination threshold of 40% is widely used in rabies elimination literature and was taken from Hampson 2009 (*doi:10.1371/journal.pbio.1000053)*. Additional clarity has been added on Lines 575-78.

***Comment:*** *Approval of human subjects is clearly documents - it does say some data is available and some is available on request. I think there is a PLOS open data policy so it would need reconciling.*

**Response:** Thanks for this important prompt. Our ethics and human subject data approvals have been clarified in the ethics section (Lines 489-93). We have now provided full set of anonymized data and code to reproduce analyses in the accompanying public Github repository now referred to in the text (Lines 482-6) and a final Zenodo repository with a doi will be included once accepted for publication.

*Reviewer #2:*

***Comment:*** *The methods appear to have some critical flaws. While the paper presents two study arms that are implied to have been given somewhat equal resources to vaccinate, and a goal of seeing which method has the better sustained coverage, it becomes clear throughout this paper that the community arm had several advantages over the teams-based approach that were not accounted for in the analysis (see below detailed comments).*

**Response:** The aim of the study was to compare two mass dog vaccination approaches through a randomised controlled trial with each arm applied in one of two study arms. These arms received similar, locally appropriate budget constraints. It was not the intention of the trial to compare two mass dog vaccination approaches using identical resources. This has been clarified on Lines 146-70 and 227-31.

***Comment:*** *Additionally, the statistical approach undertaken far exceeds the means necessary for such simplistic data. This is a rather simple 2-arm case-control study that relied on equal effort of post-vaccination survey data to compare. This can easily be presented in univariate analysis. The complex bootstrapping approaches provide no added benefit other than to befuddle the reader or potentially hide concerns with small evaluation sample sizes.*

**Response:** The reviewer makes two specific points, to which we will respond in turn: (1) that the primary analysis methods are unnecessarily complex, and (2) that the analyses using bootstrapping add no value to the analysis and are confusing.

*(1)	This is a rather simple 2-arm case-control study* [we think the reviewer meant to write cRCT, as case-control is a quite different design] *that relied on equal effort of post-vaccination survey data to compare. This can easily be presented in univariate analysis.*

The reviewer claims that the primary analysis methods are unnecessarily complex for a 2-arm cluster randomized controlled trial (cRCT). We disagree. The main analyses (the primary analysis and three of the six secondary analysis) are performed by fitting a binomial generalized linear mixed-effects model (GLMM), also known as a random effects logistic regression model, by maximum likelihood, and making inferences from the fitted model via p-values and odds ratio estimates with 95% confidence intervals. GLMMs have become increasingly popular over the last 25 years in analysis of cRCTs and are now a routine analysis method for this design (e.g. https://doi.org/10.1186/s12874-023-02107-z, https://doi.org/10.1371/journal.pone.0112653).

RCTs, and cRCTs in particular, commonly have design features that make GLMMs highly appropriate due to their ability to model multi-level data structures: data collected at repeated visits, and clustering of observations within higher level sampling units. This second feature is standard in cRCTs, where the observations are clustered within higher level units to which the intervention is applied (dogs within wards, in our study). We argue that the primary analysis GLMM is as simple as it could be while still representing the major sources of variation in coverage. As is standard when analysing repeated measures RCT data, the model contains fixed effects for time, treatment, and their interaction, because the model must allow for both the effect of the intervention and that this effect could vary over time. The model also contains random effects representing the multiple levels of sampling (district, ward, sub-village, household, and their interactions with time). Failure to use random effects to account for the multilevel structure of such data typically results in underestimation of p-values, leading to an inflated type I (false positive) error rate.

The reviewer suggests as a simpler alternative a “univariate” analysis, by which we think they might mean a univariable analysis, i.e. an analysis with a single independent variable, such as a two-sample t-test of difference in mean coverage across the six survey visits between the two arms. Such an approach would fail to exploit available information on variation within wards (e.g. variation in the number of dogs surveyed), would not allow accurate estimation of key parameters such as the intervention effect odds ratio, and would not represent variation between wards (e.g. clustering at the district level), risking false positive results as mentioned above.

Another advantage of the GLMM approach that would not be available with a univariable approach is the ability to perform the secondary analyses using the same model, by focussing on different contrasts. This approach is efficient in the sense that it minimises the number of models that need to be fitted (and explained), and it ensures that all analyses derive from a model that captures the essential sources of variation in coverage.

*(2)	The complex bootstrapping approaches provide no added benefit other than to befuddle the reader or potentially hide concerns with small evaluation sample sizes.*

Parametric bootstrapping is a standard and relatively straightforward statistical method for quantifying uncertainty in parameter estimates. We used it for two purposes. First, to estimate mean coverage and 95% CI for each arm. Second, to simulate the distribution of monthly coverage for each ward throughout the year (shown in Figure 7), to answer a crucial question for the trial: what is this probability of coverage falling below 40% in a randomly chosen ward, and how does this probability change throughout the year. We assume that it is the second, more complex, use of bootstrapping that was unclear to the reviewer. We have expanded the explanation of this method somewhat to clarify it (Lines 399-406). However, limitations of space make it difficult to provide much detail in the main text. We note however that the code implementing this method, with explanatory code comments, was available to the reviewers and will be available to readers.

Finally, we strongly refute the reviewer’s implication that this analysis was intended to “befuddle the reader or potentially hide concerns with small evaluation sample sizes.” This implication is misplaced. First, the evaluation sample size was not small; it was precisely what was required based on the a priori sample size calculation. Indeed, Reviewer #1 acknowledged that “the trial is large enough and statistically robust in coverage” and that “the statistical analysis looks rigorous and appropriate.” Second, our team has voluntarily adhered to transparency standards that exceed what is customary in veterinary research and align closely with those recommended for cluster randomized controlled trials, the gold standard in public health research (Hopewell S, et al. CONSORT 2025 statement; doi:10.1001/jama.2025.4347). These measures include preregistration of the trial, publication of the sample size calculation, full protocol and statistical analysis plan, blinding of the trial statistician, and public release of all analysis code. Such practices go well beyond standard expectations for veterinary trials and were undertaken precisely to ensure clarity, reproducibility, and methodological rigour. In this context, the reviewer’s implication is not supported and does not reflect the high level of methodological transparency demonstrated throughout this work.

***Comment:*** *Most importantly, the study should have included in the analysis a component of resource allocation to study wing and cost-effectiveness of each vaccination approach. The utilization of resources for each study wing are not presented, and therefore it is not possible for the reader to decide if either approach is truly feasible to replicate.*

**Response:** In parallel to the primary and secondary outcome measures described in this manuscript, separate analyses are being carried out including a detailed cost-effectiveness analysis and an in-depth analysis of the causal drivers of and dynamics of coverage. The aim of this present manuscript is to report the primary and secondary outcome measures which pertain to coverage achieved by the two arms of the trial. We agree with the reviewer that a cost-effectiveness analysis is important for understanding the impacts of resource allocation and these findings will be presented in subsequent manuscripts. An attempt to present all the analysis we are preparing from this trial in one paper would be inconsistent with virtually all peer-reviewed journal formats. We have clarified this in Lines 158-170.

***Comment:****Line 129 – 131 - I am not sure that the references cited back this statement. The statement seems to be implying that heterogenous coverage across an at-risk population results in sustained or rapidly re-introduced cases. I think the following references better support this claim
•* [*https://www.nature.com/articles/s41598-026-35359-y*](https://urldefense.com/v3/__https:/www.nature.com/articles/s41598-026-35359-y__;!!JmPEgBY0HMszNaDT!unGVjMqh2xsihloWmRcRI5MAc6aRP2RhAcFS_Mf712LNSrZvdMscsAHDOFmplWMMIxNa5Y8GbQk3WnSvWV-6_w$) *•* [*https://www.science.org/doi/10.1126/scitranslmed.aaf6984*](https://urldefense.com/v3/__https:/www.science.org/doi/10.1126/scitranslmed.aaf6984__;!!JmPEgBY0HMszNaDT!unGVjMqh2xsihloWmRcRI5MAc6aRP2RhAcFS_Mf712LNSrZvdMscsAHDOFmplWMMIxNa5Y8GbQk3WnTF8D2jpQ$) *•* [*https://sciety.org/articles/activity/10.21203/rs.3.rs-6648701/v1*](https://urldefense.com/v3/__https:/sciety.org/articles/activity/10.21203/rs.3.rs-6648701/v1__;!!JmPEgBY0HMszNaDT!unGVjMqh2xsihloWmRcRI5MAc6aRP2RhAcFS_Mf712LNSrZvdMscsAHDOFmplWMMIxNa5Y8GbQk3WnSiD1XmeA$) *• Frontiers | Every Dog Has Its Data: Evaluation of a Technology-Aided Canine Rabies Vaccination Campaign to Implement a Microplanning Approach
o Line 133*

**Response:** There was a reference manager error which led to the presentation of some wrong citation numbers. We thank the reviewer for noticing this. We have corrected this and all citation numbers are now correctly placed.

***Comment:*** *Onchocerciasis pharmaceutical intervention is an oral tablet for humans. Rabies intervention is an injectable vaccine for dogs. These are not really comparable, particularly as-written. The barrier is not cold chain in this context, it is that you can easily deliver a community intervention when: 1) humans are the target 2) the medication is oral 3) a licensed or otherwise-registered veterinarian is not required to administer the intervention.*

**Response:** The sentence as written led to the confusion that the reference to cold chain was linked with onchocerciasis treatment. This was not intended. The sentence has been changed to address this issue (Lines 125-128).

***Comment:*** *This is an important sentence, possibly the “thesis sentence” for this study, but it is flawed, as the principal barriers to community-directed implementation are the regulatory, clinical, and biosafety requirements associated with administering injectable biologics for a fatal zoonosis—not solely cold-chain constraints.*

**Response:** It is true that, in addition to cold-chain, there are other important barriers to community-based approaches to disease control. The paragraph has been revised to reflect this, Lines 125-128.

***Comment:*** *Line 139 - What evidence is there to support that “remote” communities have a sustained risk for rabies presence? Recent papers (see what was shared previously) show with models and empirical surveillance data that truly “remote” or “disconnected” communities are rarely (if ever) affected by rabies. If the premise of this modified dog rabies vaccination approach is necessary, please include some context about the presence of rabies cases in these targeted communities and a definition for what is considered “remote”.*

**Response:** We have edited the sentence in Line 135 and removed the word ‘remote’. We note that most human rabies deaths in Tanzania occur in rural communities where there is limited access to government services including dog vaccination (see, for example, 124 recent rabies deaths reported in: [www.medrxiv.org/content/10.1101/2025.11.20.25340719v1](http://www.medrxiv.org/content/10.1101/2025.11.20.25340719v1) these were almost exclusively from rural wards classified using the Tanzanian national government’s population and housing census). Unfortunately, surveillance systems in most sub-Saharan African countries have limited capacity hence most confirmed cases come from nearby the national vet laboratory, typically in the capital city. This may give a false impression as to where rabies cases are concentrated.

***Comment:*** *Lines 146 – 148 - This concept is oversimplified and needs additional context and thoughtfulness. The majority of dog-rabies free countries achieved elimination through mass dog campaigns in the 1920s – 1970s, long before there was reliable cold storage, digital temperature loggers, or even reliable ice boxes. Furthermore, early campaigns utilized even LESS stable nerve tissue vaccines, when compared to the modern-day cell culture vaccines available now.*

**Response:** The sentence being referred to is: *The results have the potential to shape rabies control strategies across Tanzania and similar regions in Africa where limited access to power and cold-chain storage hinder rabies elimination efforts.* The statement suggests that in areas with limited access to power, which is a large proportion of rural areas in countries such as Tanzania, the availability of community-based vaccination approaches that exploit vaccines that don’t require cold-chain storage has the potential to impact rabies control strategies. This statement seems fair and, appropriately for this paper, is primarily focused on the barrier of cold-chain storage. Nonetheless, we understand the point that the reviewer is making and have nuanced the sentence to downgrade the implied effect (Line 172).

***Comment:*** *While I prefer to avoid a competition for who can find more citations to back one’s claims – my experience is that the vast majority of campaigns and literature cite dog demography, campaign consistency, funding, and political commitment as the key barriers to dog vaccination campaigns. Rarely is temperature stable vaccines listed as a legitimate barrier, when political stability and funding are secure.*

# **Response:** We agree that dog demography, campaign consistency, funding, and political commitment are all important factors for successful mass dog vaccination campaigns. We also consider local availability of vaccines to be an important factor. In the rural areas of Tanzania where the trial was implemented, the lack of locally available vaccines is caused by several factors and the requirement to keep vaccines under cold chain conditions appears to be one. If funding was unlimited then vaccines could be brought in regularly for pulsed campaigns and there would be no need to store vaccines locally, as occurs with child immunization programs. However, in countries like Tanzania, funding for rabies control is limited and if pulsed campaigns are implemented at all they tend to be sporadic and achieve relatively low coverage. In this situation the storage of vaccines locally is important as it allows local management and coordination of mass dog vaccination. Thermotolerant vaccines are an important enabler of this. We would also like to point out that the successful eradication of other diseases has been attributed to thermostability of vaccines and social innovation (Mariner, 2012: Rinderpest Eradication: Appropriate Technology and Social Innovations ([DOI: 10.1126/science.1223805](https://doi.org/10.1126/science.1223805)))

***Comment:*** *My main point is that the “thesis statements” for this study are over simplified, and put too much emphasis on cold-chain as “THE” barrier to dog rabies vaccination campaigns, with this is simply not true. Thermostable vaccines would be a benefit, but it is not the silver bullet.*

**Response:** We concur and have revised the paragraph accordingly in Lines 125-129.

***Comment:*** *Table 2 Suggest to present the coverage as a percent, not a decimal*

**Response:** We concur and have changed the figures to reflect %s not decimals.

***Comment:*** *Line 158 - How were they classified as rural or urban? As the authors have established in the introduction that this approach is most applicable to remote communities, understanding how these are defined, particularly among the study areas, is of great importance.*

**Response:** We have removed the term ‘remote’ as per the earlier comment. The definition of whether each target community was rural or urban was made using the Tanzanian national government’s population and housing census which defines each ward as either urban or rural. Text has been added to provide information on this classification and how it was made (Lines 184-187). As was stated in line 167, the selection of wards in this trial was not stratified according to this rural / urban classification.

***Comment:*** *Figure 1- While the study utilizes stratified block randomization, the map in Figure 1 displays some geographic clustering of trial arms. Could the authors clarify if they tested for spatial autocorrelation to ensure that the proximity of similar-arm wards did not influence the observed vaccination coverage rates*

**Response:** We agree with the reviewer that, despite stratification of the randomization within districts, there appears to be spatial clustering of trial arms. Although this could be an example of the clustering illusion, where random arrangements in space look clustered (for example, multiple coin tosses produce longer runs of heads or tails than we intuitively expect), it is also possible that the randomisation has produced an unexpectedly autocorrelated pattern. We therefore followed the reviewer’s suggestion to test for spatial autocorrelation. We found no evidence (all P > 0.1) for spatial autocorrelation of the mean ward-level residuals from the primary analysis model using Moran’s *I* across a wide range of spatial scales. We have reported this analysis in Lines 442-6 and a new table, S4.

***Comment:*** *Line 201 - Is the most central village somehow demographically different than the other villages? Does these tend to be larger? Easier to reach by road? Better connected? Understanding if these are truly representative of all eligible wards/communities is important.*

**Response:** This is an interesting question. The idea of centrality is subjective (ie. its not an official central village) and we presume that the central village does not differ from the other villages in the ward, although we have not tested this. However, as the same subjective method for central village selection was used in both arms of the trial, if there were any systematic difference between the central village and the other villages of each ward, then this difference would likely be similar for both arms of the trial. We have provided some text in Lines 212-3 to explain this.

***Comment:****Line 285 – 295 - Consider a figure or schematic that visualizes the calendar/timeline and staff involved between the two vaccine distribution methods. I found it a bit difficult to understand the key differences. Also, the terminology “Team” vs “Community” is not very intuitive as to how these two methods differ. I would suggest to use terminology that clearly distinguishes the two methods.*

**Response:** We thank the reviewer for this helpful comment. We have added a month box to each arm of the flowchart. We hope this makes the calendar / timeline clearer. It is hard to add information regarding the staff involved to the figure without making it too busy. We have provided detailed text in the Methods describing the respective arm differences and have drawn the reader’s attention to this with new text augmenting the legend.

Regarding the terms ‘Team’ and ‘Community’-based, we appreciate the reviewer’s issue with these terms. The alternative options are ‘Pulse’ v ‘Continuous’ or ‘Centralized’ v ‘Decentralized’, neither of which are perfect either. After consideration, we feel that, of the three pairs, ‘Team’ and ‘Community’ most closely reflects the differences between the two arms and have opted to retain these names.

***Results*** *Reviewer #1:*

***Comment:*** *The analysis presented matches the analysis plan although derivation of the 'minimum initial coverage' required to maintain >40% by year end should be more clearly linked backed to the model assumptions described in the methods - at the moment, the reader must work to connect that.*

**Response:** We have added brief descriptive text that describes how the minimum initial coverage was calculated to the Minimum initial coverage section in Lines 574-7.

***Comment:*** *Adjustment for rural/urban imbalance between the arms should be reported to confirm robustness of findings.*

**Response:** We acknowledge that the randomization has resulted in an imbalance in land use category proportions between arms, with 82% of community-led wards being rural relative to 66% of team-led wards. We agree with the reviewer that adjusting for this imbalance would assess the robustness of our findings. We therefore re-fitted the primary analysis model, adjusting for land use category. This model did not improve fit (P = 0.17) and had negligible impact on the intervention effect estimates. We have added these results as Supplementary Table S8, and discuss them in Lines 732-37.

Additionally, in the Discussion we describe the study area as a predominantly rural region dominated by small holder agro-pastoral communities with relatively small interspersed urban areas. As such we concede that the effectiveness of the Community-based vaccination approach may vary, in larger urban areas or pastoral settings, where differences in dog ownership patterns and human population densities could influence community vaccinators' ability to reach dogs (Lines 729-32). We therefore consider that the issue of rural / urban imbalance has been addressed.

***Comment:*** *The results are clearly presented and logically structured. They are strong but sharpening to prevent over interpretation would be through providing descriptive data on the proportion of dogs reported vaccination but lacking certificates and additional operation context as to why the V1 coverage in the team based approach appears so modest relative to typical immediate post vaccination targets in typical rabies vaccination campaigns.*

**Response:** The mean coverage from the two arms under the more stringent definition of coverage (vaccination certificates were lacking) has now been added to the main text (Lines 534 – 535 and 766-9). The full output from this sensitivity analysis remains in Tables S6 and S7. On refitting the analyses using this alternative coverage definition, we decided that the household random effect showed signs of non-identifiability (slow convergence and a very large variance estimate) that did not affect models using the primary coverage definition. We therefore removed the household random effect from models using the alternative coverage definition. We have explained this in the methods (Lines 437-39) and updated Tables S6 and S7 and Figures S3 and S4 to reflect this change. This change did not affect the conclusions drawn from this sensitivity analysis.

Regarding an explanation as to why the V1 coverage in the Team-based approach was modest, this is an important point and we have addressed this with a new paragraph in the Discussion (lines 749-65) which describes how effective the Team-based approach can be when implemented by experienced and well-resourced teams. We contrast this with the findings of this study, that aimed to test the approach when implemented by government teams that typically have many communities to reach in a limited time with scant resources. In these situations, follow up transects and vaccination required to drive up coverage are often not affordable and as a result coverage achieved is often, as we have shown, relatively modest.

***Comment:*** *The figures and tables look of good quality and support interoperation. They show the stability advantage of community based approach - minor improvements would be explicit labelling in supplementary figures to aid rapid understand and a concise table summarising the variance components for easier reader interpretation.*

**Response:** We have added additional explanatory text to the legends of the supplementary figures. We wanted to draw the Reviewer’s attention to the variance component estimates which are given in Tables S4 and S6.

*Reviewer #2:*

***Comment:*** *Table 3: The authors utilize human-to-dog ratios (HDR) derived from census data to estimate the total dog population per ward. While this is a common approach, recent studies in similar ecological settings (e.g., Bangladesh) suggest that unowned or free-roaming dogs, which are often missed in household-based census estimates, can comprise a considerable number of total populations (30-40%). If the actual population denominator is higher than estimated, the reported coverage rates would naturally decrease. This has significant implications for the probability of failure analysis in Table 3 where the Team-based arm already shows a 60% probability of falling below the 40% threshold. Could the authors please comment on how the presence of an unowned dog population might influence these estimates?*

**Response:** The issue of ownerless dogs is an important one. In Bangladesh and other Asian countries, the free roaming population of dogs can be predominantly ownerless and as high as 60%. However, this is very different to the situation in Tanzania and other similar East African countries, where high levels of dog ownership means that the percentage of truly ownerless dogs is relatively low (<5%) with free-roaming dogs usually belonging to a household. As such we would not consider these to be similar ecological settings. Additionally, coverage calculations were made using the no. of vaccinated dogs in each household surveyed divided by the total no. of dogs in each household. As such, the no. of dogs / ward estimates were not used for coverage calculations and so the denominator effect is not an issue.

***Comment:*** *Figure 4: Why was the initial coverage in the Team-based arm so low at the start of the cycle? Does this reflect a failure of the "static-point" mobilization strategy compared to the local "One Health Champion" approach?*

***Response:*** Regarding an explanation as to why the V1 coverage in the Team-based approach was modest, this is an important point and we addressed this with the response to the similar comment above. That is, we have provided a new paragraph in the Discussion (Lines 750-65) which describes how effective the Team-based approach can be when implemented by experienced and well-resourced teams and we contrast this with the findings of this study, that aimed to test the approach when implemented by government teams that typically have many communities to reach in a limited time period with scant resources. In these situations, follow up transects and vaccination required to drive up coverage are often not affordable and as a result coverage achieved is often, as we have shown, relatively modest.

***Comment:*** *Line 532: If the study lacked the power to detect spatial differences, how can we be sure the Community-based approach works equally well in the more challenging urban settings, given that Table 1 showed the Community arm was 82% Rural?*

**Response:** We have dealt with the potential impact of this random imbalance between arms above, where we noted that adding land use category to the primary analysis model showed no significant effect of land use category and no impact on the intervention effect estimates. In other words, there were no significant differences in coverage between urban, semi-urban and rural wards, and even if there were differences that were not significant, they did not impact our findings. We have not, however, shown that there are no differences in the intervention effect between urban, semi-urban and rural wards, which would require an interaction test that the trial is not powered for, given that only 22 of the 112 wards are urban. We therefore draw no conclusions about differing effectiveness between these categories.

***Conclusions*** *Reviewer #1:*

***Comment:*** *The conclusions are supported by the data - the trial demonstrated that the community-based delivery achieved higher and more stable vaccination than the team based approach that was adopted annually across three years. The reduction in probability of coverage falling below the 40% threshold is clearly supported by the modelling. However, the study demonstrates improved vaccination performance not elimination of rabies transmission and better understanding of the team based approach methodology is necessary to fully compare how a community based delivery could be advantageous.*

***Response:*** As described above, we have provided a new paragraph in the Discussion (Lines 750-65) which describes the Team-based approach methodology.

***Comment:*** *The findings are highly relevant to elimination strategies and it is a fantastic study - but it is fair to say they do not directly demonstrate reduction in rabies incidence. Tightening this distinction is important so as to prevent over interpretation.*

**Response:** This is an important point and a parallel analysis investigating the public health impacts of mass dog vaccination was implemented and will be reported in a separate paper. We have added text to explain this (Lines 169-70 and 645-7).

***Comment:*** *Some limitations are acknowledged - additional limitations should be addressed such as the imbalance in rural/urban classification, reliance on vaccination certificates for defining coverage, the absence of epidemiological endpoints (eg confirmed rabies incidence in dogs or humans), sustainability and cost implications of scalability. These points don't invalidate the findings but they are important for contextual interpretation.*

***Response:***

Rural / urban classification – The issue of the rural / urban balance has been addressed in prior responses.

Reliance on vaccination certificates – a full description of the limitation of this method has been added to the Discussion (Lines 767-779).

The absence of epidemiological end points – The impact of mass dog vaccination implemented across the Mara region by the reported trial on public health impacts of rabies (dog bites and human rabies cases) was measured. These surveillance outputs are being reported separately. Text explaining this has been added (Lines 169-70 and 645-7).

Sustainability and cost implications – Similarly, data for cost-effectiveness analyses were collected and outputs are being reported in a separate paper. Text explaining this has been added (Lines 169-70 and 678-97).

***Comment:*** *The manuscript makes a meaningful contribution to implementation science in relation to rabies elimination strategies. It addresses one of the central operational challenges in how vaccination coverage can be maintained above the critical threshold over time in subsaharan Africa. It demonstrates that decentralised vaccination storage and continuous access can stabilise coverage which is particularly relevant for remote and resource constrained settings. It advances understanding of delivery tragedy optimisation which is powerful.
The public health relevance is clearly addressed, clear and timely. Its oddly be strengthened by discussing potential for scalability at national and cross border levels and clarifying how it might integrate into routine government systems without sustained external funding.*

**Response:** The sustainability of Community-based approach is discussed in the Discussion however additional text, responsive to the Reviewer’s comment describing how the costs of such an initiative might be shared between national budgets and the target communities themselves, has been added (Lines 687-93).

*Reviewer #2:*

***Comment:*** *Line 557 - Without clearly presenting the resources allocated to each study wing, it is not possible to interpret if one method was more efficient than the other. It could just be that more resources were allocated to the community approach, which of course would result in higher coverage*

**Response:** This is an interesting point and gets to the nub of what this RCT is attempting to achieve: The paper describes a pragmatic RCT in which several components (including resource allocation) are different between the arms. In such trials, the goal is to evaluate an intervention as it would be delivered in the real world*,* including whatever resources it realistically requires. In this framing, resource differences between arms are not a confounder but are part of the intervention package. For example, if one vaccination strategy inherently requires more staff or vehicles to work, then testing it without those resources would be artificial and misleading about its real-world effectiveness. This logic is common in complex health interventions, where the Medical Research Council (MRC) framework explicitly recognises that interventions cannot always be reduced to a single active ingredient.

This matters a lot in trials like mass dog vaccination versus door-to-door campaigns, for example. If one arm uses centralised fixed-point delivery and the other uses roving teams, those arms will inherently differ in resource requirements, staff time, and community engagement. Most published trials in this space do vary operationally between arms, and this is generally acknowledged. Readers are expected to interpret results with this caveat in mind.

Having said this, trials should at minimum clearly specify whether the intent is efficacy (controlled conditions) or effectiveness (real-world conditions), justify any resource differences, and conduct economic analyses in parallel to the trial so that cost differences are quantified rather than buried.

With this in mind, we have edited the text to make it more explicit that the reported RCT was testing the real-world effectiveness (and not the efficacy) of two different strategies, and that as a result the strategies did experience variation in operational resources. Where appropriate, the word ‘effectiveness’ has replaced ‘efficacy’ throughout and text explaining this RCT approach has been added to the Intro (Lines 146-70 and 227-31).

***Comment:*** *Figure 4 and line 559 - This is a recurring theme of my review, but without knowing resources allocated, these conclusions seem more like “common sense” than scientific findings. Figure 4 seems to show an equivalent amount of resources spent at the start of each year in both study wings (both red and blue have large spikes) and then red (community) vaccines are distributed at smaller amounts throughout the year. So it appears that equal resources were not allocated to both approaches to determine if one is more efficient and effective than the other. The real study question should have been, “if resource allocations are equally allocated, is a pulse vaccination (could even be 2-times a year) less effective at maintaining coverage than consistently available vaccination”. If, all resource allocations being equal, the continuous had higher coverages, this would have been a very convincing study on a new approach that directly conflicts with current guidance from the World Health Organization.*

**Response:** We draw the Reviewer’s attention to the response to the last comment. Additionally, Figure 4 is showing that throughout the year there was a demand for rabies vaccination and, when resources are provided that allow vaccination to take place, this demand can be met. If the Team-based pulsed approach was more effective as a strategy (that is if dogs weren’t born during the year that needed vaccination, if new dogs weren’t brought into the village, and if everybody with a dog brought their dogs for vaccination on vaccination day) then there would not be a demand for continuous provision of vaccines throughout the year. Whilst it may seem common sense, the logic of this approach needed to be demonstrated through a complex intervention RCT such as this.

***Comment:*** *Line 566- Many high-performing “team based” / “pulse” vaccination campaigns DO reach >61% of dogs during their pulse. What was it about your program’s approach that led to such abysmal “pulse” coverage results, particularly three years in a row for the team approach? These low coverages may reflect campaigns at their infancy, but many rabies programs with some extant of experience will have no problem reaching 61% with an annual campaign approach. What is the struggle is ensuring funding is maintained year on year.*

# **Response:** This is an important point as it gets to the difference between a) non-governmental-led / donor-funded campaigns and b) campaigns led by national governments. To eliminate human rabies, national governments are likely to be needed to play a major role in financing, managing and coordinating mass dog vaccination at scale across countries. Donor-led initiatives may not be best positioned to achieve the scale required, and indeed there are few examples where such initiatives are achieving disease control at any meaningful scale. As such, this study aimed to compare two pragmatic strategies that could be implemented by national authorities. In such a situation, team-based approaches are budget and time constrained and frequently achieve only moderate coverage at best, as has been shown in numerous studies (e.g., Chazya 2024, Rabies vaccinations at the rural–urban divide: successes and barriers to dog rabies vaccination programs from a rural and urban campaign in Zambia, [doi.org/10.3389/fvets.2024.1492418](https://doi.org/10.3389/fvets.2024.1492418); Ferguson 2020, Volunteer based approach to dog vaccination campaigns to eliminate human rabies: Lessons from Laikipia County, Kenya, [10.1371/journal.pntd.0008260](https://doi.org/10.1371/journal.pntd.0008260); Akankwatsa 2025 Comparing vaccination coverage and dog population demographics among four pilot dog rabies vaccination strategies in Uganda, [doi.org/10.3389/fvets.2025.1656563](https://doi.org/10.3389/fvets.2025.1656563) etc.).

***Comment:*** *Also somewhat concerning, it does not look like the number of dogs vaccinated by the team approach increased year-on-year, which is what is expected when vaccination campaigns are adequately supported and implemented in a community. The stagnant vaccination effort for 3-continuous years at sub-par coverages in the “team” approach is highly concerning for the approaches applied by the authors. Many programs would not tolerate consistently low coverage year-on-year, and would therefore likely not find these study results representative of their approaches to pulse vaccination.*

**Response:** The Team-based method tested is the one that is applied by the Government of Tanzania when the Directorate of Veterinary Services (DVS) implements mass dog vaccination. The resources provided were equivalent to those provided by the DVS and the advertising methods were similar. In our experience initial vaccination coverages can be lower if it is the first time that an approach is used, especially in new communities (Sambo, 2022, [*https://journals.plos.org/plosntds/article?id=10.1371/journal.pntd.0010124*](https://journals.plos.org/plosntds/article?id=10.1371/journal.pntd.0010124)*),* but once communities are sensitised the coverage achieved tends to plateau and not increase year on year. We are not familiar with evidence of growing coverage with each year anywhere else. Rather, if rabies cases have been controlled, fatigue can set in with dog owners not considering it necessary to repeatedly have their dogs vaccinated and consequently coverage can decline.

***Comment:*** *Line 576 - This is really antiquated thinking on dog vaccination in the African setting. Numerous publications have been released in the last decade showing that, with proper resource allocation, dog vaccination coverages can be reached. The challenge has been maintaining resources for the 10 – 15 years it is estimated it takes to eliminate rabies from a large area.*

**Response:** We agree with the reviewer that ‘with proper resource allocation’ dog vaccination coverage approaching 70% can be reached. However, as described in the previous responses, this trial was comparing two strategies that could be implemented by national authorities. In such a situation, team-based approaches are budget and time constrained and frequently achieve only moderate coverage at best. The publications referred to by the reviewer likely describe non-governmental managed / donor funded initiatives which undoubtedly do achieve higher coverage because they have the resources to do so. However, we would argue, this is not a sustainable model of vaccine delivery at the scale required to control rabies.

***Comment:*** *And along these lines, resource cost and availability (personnel and funding) are a critical component of any health intervention in low- and middle-income countries. It is difficult to interpret results such as those presented here without also presenting the personnel, logistical, and financial resources necessary to operate these two vaccination approaches.*

**Response:** We draw the reviewer’s attention to previous responses that have dealt with this important point.

***Comment:****-*  [*https://journals.plos.org/plosntds/article?id=10.1371/journal.pntd.0008004*](https://urldefense.com/v3/__https:/journals.plos.org/plosntds/article?id=10.1371*journal.pntd.0008004__;Lw!!JmPEgBY0HMszNaDT!unGVjMqh2xsihloWmRcRI5MAc6aRP2RhAcFS_Mf712LNSrZvdMscsAHDOFmplWMMIxNa5Y8GbQk3WnT9cqTqFA$) *and the experiences in Chad would conflict with the author's statements here.*

**Response:** This is a good example of the type of vaccination campaign that can be implemented by donor-funded non-governmental organizations such as Mission Rabies. We quote from the Methodology:

The campaign was advertised via posters, newspapers and educative visits to schools. The SP stage ran from April 22^nd^ to December 13^th^. Working timespans for each region are described in [Table 1](https://journals.plos.org/plosntds/article?id=10.1371/journal.pntd.0008004#pntd-0008004-t001). In the urban regions, static vaccination points were set up in fixed locations, while for rural regions they were set up in predefined points or *ad hoc* depending on the location and the type of community, with smaller communities being roamed by vehicle, from where the vaccinations were announced. Animals brought to these clinics received parenteral vaccination (Nobivac Rabies, MSD Animal Health), were marked to prevent re-vaccination, and their owners were given a vaccination certificate. The D2D stage, developed to complement the SP vaccinations, ran from April 24^th^ to December 13^th^. An increased effort was put into the D2D vaccination of rural regions in order to bring high coverages across the entire working area. During this stage, the vaccination teams covered different areas of the regions each day, knocking on household doors along their path, and offering vaccination to both indoor dogs and to dogs on the street. Free-roaming dogs with no identifiable owner were also vaccinated, using catching nets for those difficult to approach. All staff involved were trained to perform the vaccination humanely and causing minimum distress to the dog. After each cycle of SP and D2D vaccination, a survey was conducted in order to assess the vaccination coverage achieved.

In each location the campaign involved multiple day static point, door to door, catching of dogs with nets and post-vaccination monitoring. This is a textbook method that will enable high coverage to be achieved. Unfortunately, given national governmental budget constraints, its not likely to be scaleable or sustainable. As described, the approaches that were tested in this study were designed to be approaches that could be realistically implemented by national governments and, indeed, the method of the standard Team-based approach mirrored the method used by the Govt. of Tanzania.

***Comment:*** *Line 579 - Is there any data to show that rabies cases declined, as the theoretical critical vaccination threshold would suggest they should?*

**Response:** The impact of mass dog vaccination on public health impacts of rabies (dog bites and human rabies cases) was measured and rabies transmission reduced, with multiple public health benefits, compared to other regions without this region-wide MDV. These surveillance outputs are being reported in a separate paper. Text explaining this has been added (Lines 169-70 and 645-7).

***Comment:*** *Lines 583 – 595 - This is all theoretical, with no supporting data in the results section. Show the reader the bite data from these communities, definitively prove bite events went down. Show the reader the rabies surveillance data from these communities to prove that vaccination thresholds were met (or not). The authors spend 3 years supporting rabies control in these communities, it is unreasonable that their evaluation should not include direct measures of effect, rather than relying on modeled estimates and (per this paragraph) theoretical musings.*

**Response:** As per the previous comment, we did measure the impact on public health of the mass dog vaccination trial implemented across the Mara region. These surveillance outputs are being reported in a separate paper. Text explaining this has been added in Lines 169-70 and 645-7.

Further, regarding the Discussion text referred to, the text describes that as coverage increases so the time taken to achieve rabies control will likely decrease. We agree this is theoretical, although logical too. The text that the Reviewer is referring to is in the Discussion section and as such we consider that this is an appropriate place for a discussion regarding the relative merits of increasing vaccination coverage. We have added some text to provide additional nuance to indicate the theoretical nature of the discussion (Lines 637-641).

***Comment:*** *Line 604 - If community involvement was the key difference between vaccination approaches, that was not clearly explained. As stated earlier, better documentation of the resources allocated to each approach, better explanation of the personnel needed and the timelines of activities, all would make statements such as the one at line 604 easier for the reader to agree with.*

**Response:** In line 604 we wrote **‘***In the Community-based intervention tested in this RCT, communities were meaningfully involved at various levels*…’. We then go on to list the ways in which communities were involved in the intervention. We do not state that community involvement was the factor that led to the impacts seen. However, the reviewer makes a good point and that is that it was the intervention as a whole that was being tested in this paper and not any one component of it. As such, we do not state that it was the community involvement or the continuous availability of vaccines or the decentralized nature of the intervention that made the difference. We are developing separate manuscripts that home in on some of these specifics. We are mindful of this and have reviewed the manuscript to ensure that this does not happen.

***Comment:*** *606 – 610- What resources (funding) was necessary to utilize the continuous services of these local people? And, while it may be that these people are already paid by other programs and no additional funding was required for the purposes of this study, SOMEONE paid for their time. Surely, they were not volunteers (and it would be ethically questionable if this study asked them to volunteer their time over such a long period). Even if they were paid for by another source, the sustainability of such an approach would require a formalized agreement of resource sharing; did this happen? If so, please make that a prominent feature and success story of this effort; as I would doubt this is reproducible or sustainable without this.*

**Response:** The reviewer is correct to raise the issue of the local community participation and sustainability - as the development and testing of sustainable interventions are key objectives of this work. That is why established government and community-based systems were used to implement both the Team- and Community-based delivery strategies. In doing so, we attempted to use an infrastructure that, unlike donor funded initiatives, would be locally available, sustainable and scalable. As described in previous responses, economic data was collected, including estimates of dog owner time and time cost, and cost-effectiveness analyses were carried out in parallel to the outcome measures reported in this paper. As we have described, detailed economic analyses will be published in separate papers.

***Comment:*** *618 – 620 - Again, this is an oversimplified and misleading statement. Pulse campaigns also can heavily rely on local leaders and rabies champions to “meaningfully control in their own area”. The difference the authors claim to try to be showing per the stated goals is not the engagement of locals, but the continued presence of vaccine AND the continuously available RESOURCES to make that vaccine available to community members. Implying that pulse-based campaigns are devoid of local leadership is not correct and very few rabies programs would even attempt such a thing.*

***Response:*** In lines 618-20 we are listing components of the Community-based approach where community actors had a meaningful role to play. We are not stating that the Team-based approach does not rely on local leaders and we are not implying that pulse-campaigns are devoid of local leadership. The description of each strategy is given in the Methods where we describe the personnel involved in each approach. It is our experience, however, that team-based approaches, whether implemented by governments or donor-funded non-governmental organizations, are typically coordinated and implemented by people from outside of the immediate target community / village with community members being given relatively minor responsibilities, such as individual “village coordinators” who provide local introductions to leaders and advertising the event. We have provided substantial new text to explain the resources issue and to augment the original descriptions of the two approaches in Lines 146-56 and 265-345.

The reviewer mentioned the stated goals. This is an important point as the stated aims / goals need to be clear so that confusion can be avoided. As such, the stated aim of the study ( Lines 158-170 ) has been augmented to reflect this.

***Comment:*** *Lines 623 – 624 - Exactly – the community approach seems biased from the start and it is unclear if this was due to study design or local implementation factors. Clearly additional resources were made available to the community approach – biasing any direct comparison between methods. A better design would have been to forgo a pulse campaign in the community approach, and rely entirely on continuous availability. However, as it stands, 2 critical limitations seem to be affecting this design:
• First, there appears to be some community-based bias towards the pulse-component of the community approach.
• Second, resource allocation was not equal*

**Response:** We have already addressed the point about the objective of the RCT being to compare the effectiveness of two complex interventions and to evaluate them as they would be delivered in the real world*,* including whatever resources they realistically require (Lines 138-170). Another focused comparative analysis of the impact of pulse delivery (as in the team-led arm) versus continual delivery, versus a hybrid delivery (as in the community-led arm) is in development.

***Comment:*** *Line 625 - Why could this one health champion not also be involved in these planning activities for a pulse campaign? Again, it seems approaches were not equivalent between the two study arms, particularly when it comes to planning the “pulse” component that both study arms were allowed to conduct. As such, comparison between the two approaches is likely not valid.*

**Response:** We draw the reviewer’s attention to our last response.

Regarding the One Health Champion, indeed this cadre could be established within the Team-based approach, however this is not what typically happens in government-led team-based pulsed mass dog vaccination campaigns in East Africa at least. Typically, these campaigns are implemented using the team structure utilised in the RCT, which does include a village-based assistant who is familiar with the community, knows where dogs might live, encourages owners to bring dogs, and helps with the practical aspects of the day. This position is, however, different from the One Health Champion who was a village leader and had a more permanent role beyond the campaign day, with responsibilities including identifying where new litters of dogs might be, or where dogs that missed vaccination lived and then informing the Rabies Coordinator so that follow up vaccination could occur using vaccines that were stored in the community. New text augmenting the team descriptions has been provided in Lines 226-345.

***Comment:*** *Line 627 - The interplay of what appears to be a study design flaw should not be investigated for future publications. It should be investigated, explained, and accounted for within the study in which the design flaw occurred!*

**Response:** The study design is appropriate for complex interventions, was intended as such, and was funded as such. We draw the reviewer’s attention to our last two responses. The scope of what the reviewer is asking for in this paper would change the intent of this paper and would lead to an exceedingly broad scope. As is common in these circumstances, we are preparing a set of complementary manuscripts to provide focused treatments that together provide a holistic view of the trial outcomes.

***Comment:*** *Line 643 - The authors need to more clearly describe these settings and the definition of “remote”. A goal of the authors was that these results could be applied to similar settings. In my experience, rabies endemic communities with complete lack of "access" to electricity are uncommon. The authors should provide some characterization of these communities in a manner that supports extrapolation to other countries that they deem similar and they need to provide data that establishes that these remote communities are even affected by enzootic dog-mediated rabies in the first place. There was no data presented to suggest that rabies is even present in these settings.*

**Response:** We have addressed the issue of using the word ‘remote’ in an earlier response. Regarding electricity, whilst provision of electricity is slowly increasing in rural areas of countries like Tanzania access is often very limited and variable in its delivery and consistency. Additionally, as most villages do not have a health clinic, vaccines would often need to be stored somewhere else, such as the offices of village leadership. These offices are typically very basic and without power.

Regarding whether these communities are even affected by rabies, perhaps the reviewer is not familiar with rural Tanzania where cases of dog mediated human rabies occur very regularly and, given the readership of the journal, it doesn’t seem appropriate to have to provide data to underscore the point that rabies is present in these endemic areas.

***Comment:*** *Line 655 - Agree with this statement, but this does not require zeepots. Many places could/should offer continuously available vaccine AND they DO have electricity. The authors are limiting the relevance of their publication by continuing to imply that continuous vaccination is purely a limitation of thermotolerance. It is not. The limitation is resources, staff, funding, and better knowledge of the Cost-effectiveness of a continuous approach. Unfortunately, none of this is provided in the analysis of this study.*

**Response:** We are pleased that the Reviewer agrees with the statement ‘*Having locally stored vaccine continuously available will provide Rabies Coordinators and One Health Champions with improved agency to vaccinate at times that suits their needs.’* However, the realities of the rural communities in which the RCT took place, and that are typical of many across Tanzania, are that many of these villages don’t have reliable access to power and as such they don’t have continuously available vaccine. We agree with the reviewer that the lack of continuous vaccination is partly due to *‘resources, staff, funding, and better knowledge of the Cost-effectiveness of a continuous approach’,* but it is most definitely also due to a lack of reliable power and refrigeration such that vaccine storage of non-thermotolerant vaccines is not possible.

We have already addressed the point about the objective of the RCT being to compare the effectiveness of two complex interventions and to evaluate them as they would be delivered in the real world, including whatever resources they realistically require (Lines 146-70).

***Comment:*** *Line 682 - As much as I want to agree with the authors (and they are probably right) – the study design and lack of consideration of cost/resources makes me doubt if this study truly does show that there is potential in this community delivery method. Flying vaccine by a drone every day to a vaccinator likely would also show “potential”, however the costs to do so make this non-feasible. Without consideration to costs and resources necessary to operate the continuous model, there is little ability to make claims on the feasibility, reproducibility, or acceptability of this approach.*

***Response:*** We have already addressed the point about the objective of the RCT and this manuscript being to compare the effectiveness of two complex interventions and to evaluate them as they would be and were delivered in the real world subject to comparable overall resource constraints (Lines 146-70).

***Editorial and Data Presentation Modifications?*** *Reviewer #1:*

***Comment:*** *The manuscript is well written and clearly structured.
Suggested minor revisions would be:
Clarifying representativeness of the team-based arm, especially in light of relatively modest V1 coverage estimates compared with typical post-campaign results in similar settings.*

***Response:*** The personnel of the team- and community-based interventions is detailed in the Intervention Teams section of the Methodology. This section has been augmented with new text to clarify that the Team-based structure was designed to represent a typical government-led mass dog vaccination initiative and not a structure implemented by a donor-funded non-governmental organization (Line 225-345). Further, the text at the end of the Introduction describing the overarching aim of the study has been augmented with text describing that the Team-based approach was designed to reflect typical team-based vaccination units that are employed by governments (and not NGOs), such as the Government of the Republic of Tanzania, to implement mass dog vaccination (Line 138-70).

***Comment:*** *Clarifying data availability statement to ensure full alignment with PLOS open data policy (public repository vs “available upon request”).*

***Response:*** We have provided anonymized data and code to reproduce analyses in the accompanying public Github repository now referred to in the text (Lines 480-86) and a final Zenodo repository with a doi will be included once accepted for publication.

***Comment:*** *Report sensitivity adjustment for rural/urban imbalance between arms to confirm robustness of findings.*

***Response:*** This issue of urban / rural balance has been addressed with a previous response.

***Comment:*** *Provide a brief clarification of modelling assumptions underlying the “minimum initial coverage” and probability of falling below 40%.*

***Response:*** We have added brief descriptive text that describes how the minimum initial coverage was calculated to the Minimum initial coverage section in Lines 575-9.

***Comment:*** *Tighten the conclusion language to clearly distinguish improved vaccination coverage from demonstrated interruption of rabies transmission to prevent over interpretation.*

***Response:*** In the Conclusion we state the Community-based approach led to increased coverage and that, as such, we consider this could be an important approach for inclusion in national rabies elimination strategies. We do not mention interruption of rabies transmission in the Conclusion and so have not made any substantial changes to this section.

*Reviewer #2: (No Response)*

***Summary and General Comments***

*Reviewer #1:*

***Comment:*** *This is an important, large and well-designed, three-year cluster randomised controlled trial, comparing community-based continuous delivery of mass dog vaccination with standard annual team-based delivery. The objectives are clearly articulated, and the hypothesis - that vaccination coverage differs between delivery strategies - is clear and testable. I found the study engaging, and the operational insights highly interesting.
The study design is appropriate and rigorous. The “fried-egg” approach to minimise contamination is both thoughtful and well justified. The population is clearly described and appropriate for testing the hypothesis in endemic rural African settings. However, generalisation beyond this context should be framed with caution. Scaling to larger urban environments or addressing cross-border transmission networks would require further evaluation. The intervention model is clearly well suited to endemic, decentralised rural systems. Its applicability beyond similar sub-Saharan African contexts should be explicitly qualified in the discussion.*

***Response:*** We have added text to the Discussion that clarifies that we do not draw conclusions regarding the suitability of the Community-based strategy in countries with dissimilar agro-ecological settings (**Lines 729-32).**

***Comment:*** *The sample size (56 wards per arm) appears sufficient to detect meaningful differences in coverage, and power calculations are clearly described. The trial is statistically robust for its stated endpoint - vaccination coverage. It is not powered to assess epidemiological outcomes such as confirmed dog rabies incidence or human exposure trends. This doesn’t weaken the study’s objective, but limits conclusions regarding elimination impact, and this distinction should be made explicit.*

***Response:*** We agree with this statement and have discussed theoretical conclusions regarding the possible impact of improved coverage on rabies transmission in the augmented Benefits of Increasing Coverage section of the Discussion (**Lines 637-42**).

***Comment:*** *The statistical analysis is rigorous and appropriate. Additional clarity around assumptions used in estimating the “minimum initial coverage” required to maintain >40% by year end - and the modelling of probability of falling below 40% - would improve interpretability.*

***Response:*** This comment has been addressed previously***.***

***Comment:*** *Adjustment for rural/urban imbalance between trial arms should also be reported (or presented as sensitivity analysis) to confirm robustness of findings.*

***Response:*** This comment has been addressed previously***.***

***Comment:*** *The results are clearly presented and logically structured. The demonstration of greater stability of coverage in the community-based arm is compelling. To prevent over-interpretation, the manuscript would benefit from:
Providing descriptive data on the proportion of dogs reported vaccinated but lacking certificates.*

***Response:*** This comment has been addressed previously***.***

***Comment:*** *Providing additional operational context explaining why V1 coverage in the team-based arm appears modest relative to typical immediate post-campaign coverage targets achieved consistently in similar settings.*

***Response:*** This comment has been addressed previously***.***

***Comment:*** *Clarifying more fully how representative the team-based approach is of “standard practice.”*

***Response:*** This comment has been addressed previously***.***

*Figures and tables are of good quality and effectively demonstrate the stability advantage of the community-based approach.*

***Comment:*** *The conclusions are supported by the data presented. The trial clearly demonstrates that community-based delivery achieved higher and more stable vaccination coverage across three years, with a substantially lower probability of falling below the 40% threshold. However, the study demonstrates improved vaccination performance, not elimination of rabies transmission. While highly relevant to elimination strategies, it doesn’t directly demonstrate reductions in rabies incidence. Tightening this distinction would strengthen the manuscript.*

***Response:*** This comment has been addressed previously***.***

***Comment:*** *Some limitations are acknowledged. Additional limitations that should be explicitly addressed include: rural/urban imbalance between arms; reliance on vaccination certificates for defining coverage; absence of epidemiological endpoints; and sustainability and cost implications for national scalability. These do not invalidate the findings but are important for contextual interpretation.*

***Response:*** These comments have been addressed previously***.***

***Comment:*** *Overall, this manuscript makes a meaningful and important contribution to implementation science for rabies elimination. It addresses one of the central operational challenges: how vaccination coverage can be maintained above critical thresholds over time in resource-constrained settings. The demonstration that decentralised vaccine storage and continuous access can stabilise coverage is particularly relevant for endemic regions of sub-Saharan Africa.
Public health relevance is clear. Discussion would be further strengthened by consideration of national scalability, cross-border coordination, and integration into routine government systems without sustained external funding.*

***Response:*** This comment has been addressed previously***.***

***Reviewer #2:***

***Comment:*** *I do believe that continuously available rabies vaccine, as is the model in nearly all upper income countries, is a long-neglected and promising approach to dog vaccination in endemic countries. However, the study design and analytic approach in this manuscript does not convincingly demonstrate that one approach is better than the other and certainly does not show that one is more efficient or effective than the other. The authors spent 3 years in these communities, yet were not able to increase coverages year-on-year using the standard “team” or “pulse” approach – this is highly concerning.*

**Response:** We implemented an RCT to investigate the difference in coverage achieved by two mass dog vaccination strategies. The RCT lasted three years. We have presented the coverage results which show that a different level and consistency of coverage was achieved by the two strategies. It is important to note that our role as RCT researchers was not to try to increase coverage in either arm or to direct resource use differentially across arms, as this would have biased the results. Again, we draw the reviewer’s attention to our previous response that we implemented a pragmatic RCT in which the goal was to evaluate an intervention as it would be delivered in the real world*,* including whatever resources it realistically required. To preserve the validity of the RCT, we could not intervene to try to improve coverage.

***Comment:*** *These communities also had 3-year sustained vaccination support, but there is no discussion about rabies surveillance activities conducted over this same time frame.*

***Response:*** This comment has been addressed previously***.***

***Comment:*** *Why would the authors rely on modeled and extrapolated dog vaccination coverages (which in themselves have a theoretical cutoff for protection) when instead they could have demonstrated through surveillance whether or not effective herd immunity had been reached. This would have been the most convincing outcome of all.*

***Response:*** The primary and secondary outcome measures in the reported RCT were related to coverage. This is a well reported measure of the reach of vaccination programs. To demonstrate whether effective herd immunity had been reached would have required a completely different study design. As has been described above and in the manuscript, we did measure the impact on public health through Integrated Case Bite Management the findings of which will be reported, as is appropriate with large RCTs with multiple outputs, in a separate paper.

***Comment:*** *As-presented, the study provides a rather common-sense outcome “if you provide more vaccine, more dogs will get vaccinated”. The resources allocated to each study wing are a critical component of this study and its interpretations, however they are not presented. Showing the cost-effectiveness of these approaches is necessary for this to be publishable.*

***Response:*** Much of this comment has been addressed previously***.*** Regarding the statement, *“if you provide more vaccine, more dogs will get vaccinated”,* we don’t think that this necessarily holds true in all situations. For example, providing more vaccine to a static point pulse campaign does not mean more dogs will be brought in for vaccination. In our experience the number of doses does not impact the number of dogs that are vaccinated. In fact, our teams tend to return to base with excess doses. Rather, it’s the time frame that these doses are available (ie. continuously throughout the year) and how they are managed (i.e. by local people who are known to the community and who know where the unvaccinated dogs live) that impacts coverage. This is what the RCT was testing.


***Comment:*** *- Was COST EFFECTIVENESS assessed? Line 138 says there is hope for cost-effective rabies control programs, but it does not appear that costs, resources, or effectiveness (decline in cases) were included in this paper.*

**Response:** This comment has been addressed previously***.***

***Comment:*** *Was any kind of rabies burden measured (e.g., number of human rabies deaths or confirmed canine rabies cases or dog bites treated) before and after the intervention? Ideally, vaccine coverage should be correlated with a decrease in disease burden.*

**Response:** This comment has been addressed previously***.***

***Comment:*** *The results need to include measures of resources allocated to each study wing. Of most importance, how many person-hours were spent under each method to vaccinate these dogs? Without this information, it simply looks like the conclusion is that doing more campaigns throughout the year results in a more stable vaccination coverage; that is not novel or complicated and certainly does not require this level of statistical effort to prove this cause-effect relationship. If the team-approach had been conducted at 2 timepoints through the year, how would that have compared in resource-needs and estimated coverage compared to the community approach?*

***Response:*** This comment has largely been addressed previously***.*** Regarding the comment, ‘*If the team-approach had been conducted at 2 timepoints through the year, how would that have compared in resource-needs and estimated coverage compared to the community approach*?’, this is a good question and is the type of question that we were asking. However, rather than compare a one pulse to a two pulse system, we compared a one pulse to a ‘continuous’ system. Both questions are valid and require the rigor of an RCT to investigate their effectiveness.

***Comment:*** *I also find it concerning that the team-approach spent only one day per community. It is well-established that offering vaccination for just one day in remote or hard-to-reach communities is not an effective approach, as owners or dogs may not be available at that specific day or time. Many successful campaigns will spend several (three) days per community to maximize coverage.*

**Response:** This comment has been addressed previously***.*** The reviewer makes a good point about the limitation of teams targeting communities for just one day for the reasons given. However, due to resource constraints, that is the approach that is generally used by national governments and is the approach we tested the novel strategy against.

***Comment:*** *The resources allocated to each method are not clearly presented, which makes it very difficult as the reader to not only fairly compare the outcomes presented here, but also to understand if the community approach is “better” / “efficient” or if it just represents more vaccination effort.*

**Response:** This comment has been addressed previously***.***

***Comment:*** *Where is the surveillance data for this region? The author spent 3 years supporting rabies control in these communities, surely there is some effort towards surveillance as well? The goal of vaccination is not to achieve a model-derived threshold of coverage – it is to see cases decline. The over-reliance on model and extrapolation-derived vaccination coverages, rather than surveillance data is disappointing. I have a similar concern about the discussion statement about dog vaccination coverages leading to reduced bite events – this was not presented in the results (although probably should be).*

**Response:** Both components of this comment have been addressed previously***.***

***Comment:*** *Where are the rest of the references, they only go through 26*

**Response:** We thank the reviewer for pointing this out however we aren’t sure why only 26 references were viewable as the full (32) list was included in the submitted pdf. We will endeavour to make sure that this is addressed at the next submission.

***Comment:*** *In nearly all countries there are legal requirements on who can administer rabies vaccines. It was not clear who was vaccinating dogs in the community approach, if these were legally-approved vaccinators. If there were local approvals that were unique to this study that allowed non-traditional vaccinators, this is a critical aspect of the approach that needs to be explained in the manuscript. If this was the approach taken, not all countries will be able to get these approvals, but a clearly written example of how this was done in Tanzania would be of great benefit.*

***Response:*** This is an important point, and we thank the reviewer for pointing it out. In the Intervention Teams section of the Methodology, we described the personnel of each strategy and the role that Livestock Field Officers (LFO) played in both. However, we had not explained that in Tanzania LFOs are certified government employed veterinary paraprofessionals who are legally permitted to treat animals and administer vaccinations. We have added text to the Methods section to further explain this (Line 225-31).

***Comment:*** *The over-reliance on the statistical approach is not justified given the simplicity of the data collected. Honestly, a simple table of approach and measured coverage by V/Y would have sufficed for the entirety of the analysis.*

***Response:*** We have addressed the reviewer’s concerns about the complexity of the analysis above.

***Comment:*** *The study authors imply a simplistic study design where the only altered variable was the continuous availability of vaccines. However, throughout the methods and discussion it becomes clear that there were several critical variables that differenced between the study wing, including the planning and community sensitization approach and the involvement of local collaborators. These are variables that are not inherent only to continuously available vaccine approaches. Local stakeholders and strong community sensitization pre-campaign can also be achieved during pulse campaign efforts. Since multiple variables were altered between the two study designs, it becomes impossible to distinguish which factors led to the improved vaccination coverages (although there is an element of common sense throughout this entire paper): the differing pre-campaign sensitization? The differing level of community involvement? Differing levels of resources allocated to each wing? The continuous availability of vaccine?*

***Response:*** We have addressed this point in detail previously*.* As mentioned, this is an important point and allows us to explain what the objectives of an RCT investigating complex interventions are. As such, we have provided more text in the manuscript to underscore this point. Please review earlier comments on the same issue.

***Comment:*** *It is concerning that the “pulse” component of each study arm showed significantly higher coverage in the community approach, when theoretically they should have had similar resource-availability and outcomes for this specific vaccine component. This brings into question if there were incentives given to staff (ie money) and if those incentives differed by study wing. Example, if the team-based staff were paid on salary from the central government but local collaborators in the community-based approach received a payment above and beyond their routine day-job income, then there is an increased incentivization for the community staff when compared to the salaried government workers. Perhaps this was not a concern, however with the lack of transparency of resources allocated to each wing (e.g. funding, personnel, incentives, logistical support), it is impossible for the reader to understand potential bias.*

***Response:*** This comment has been addressed previously***.***
