# Supplementary material for: Effectiveness of community-based delivery of mass dog vaccination to prevent rabies: A cluster randomized controlled trial
Source: PLoS Negl Trop Dis. 2026 Sep 25;20(9):e0014704. doi: 10.1371/journal.pntd.0014704 (PMC13641700; doi:10.1371/journal.pntd.0014704)
Supplement: Supplementary file 12 [file pntd.0014704.s016.docx]

**Response to Reviewers:**

We thank the two reviewers for their time reading and reviewing the manuscript. We are pleased that Reviewer One found that the manuscript was substantially improved with many of the previous concerns thoughtfully addressed. We note, however, that the reviewers consider that several issues remain. We have summarised and addressed each of these issues below. (The full text of the reviewers’ comments has been copied in full at the end of the response text.)

Point 1: Applicability beyond the specific epidemiological and operational context studied should remain carefully qualified. The intervention appears particularly suited to decentralised rural endemic settings typical of parts of sub-Saharan Africa. Generalisation to dense urban settings, highly mobile dog populations, or different sociocultural contexts requires further evidence.

Response: This is an important point and is one that we addressed in the last round of revisions. Please see the Impact of Geography sub-section of the Discussion (lines 819-61) where we discuss in detail that the trial results emanate from a predominantly rural region dominated by small holder agro-pastoral communities with relatively small interspersed urban areas. We state that the effectiveness of this approach may vary in larger urban areas or pastoral settings, where differences in dog ownership patterns and human population densities could influence community vaccinators' ability to reach dogs. We continue that to achieve widespread rabies control across endemic countries such as Tanzania, composed of mosaics of agro-ecological areas and landscapes, comprising varied human and domestic animal densities, control efforts will likely require locally tailored mass vaccination approaches that exploit different delivery strategies. Further, we caution against drawing conclusions regarding the suitability of the Community-based intervention in rabies endemic countries that do not share these agro-ecological similarities, for example in countries in Asia where landscapes and domestic dog demographics can be very different. Consequently, we feel that we have sufficiently addressed this point. However, we have added a clause to the abstract and the author summary to make sure that this important point is understood in these summarised sections (lines 73 and 100-2).

Point 2: Interpretation of the comparator arm and the wording describing “standard” team-based delivery should be moderated. The Discussion should more clearly distinguish between the specific comparator tested in this trial and broader team-based approaches used elsewhere in Africa and Asia.

Response: Differentiating between the team-based approach implemented by NGOs and the team-based approach implemented by the ministries responsible for animal health is important. Indeed, this was a point that was raised and addressed in the last review. For example, in lines 163-6) of the Introduction we are clear that the tested Team-based approach was designed to reflect typical team-based vaccination strategies employed by governments, such as the Government of the Republic of Tanzania, to implement mass dog vaccination. Further, in the Discussion (lines 863-78), we dedicated a sub-section (Team-based arm) to discuss the fact that the coverage achieved in the Team-based arm of the trial was relatively modest and that this contrasts with the coverage achieved by team-based mass dog vaccination campaigns implemented by NGOs in Africa and Asia where coverage in excess of a target of 70% can be achieved. However, such high levels of coverage is not typical of government-led programs and, as such, this text is followed by a detailed explanation as to why team-based mass dog vaccination implemented by non-governmental organisations might be more effective at reaching higher levels of coverage.

Despite this, we have further emphasised this with an additional sentence in lines 163-6 that states that the Team-based approach was not designed to reflect the approach commonly used by non-governmental organisations that often have the resources needed to implement post-vaccination coverage assessments and targeted follow-up vaccination efforts. Additionally, where appropriate, we have removed the word ‘standard’ as a descriptor of the Team-based approach.

Point 3: Clarification should be included regarding how vaccinated dogs were identified during post-vaccination assessment. The limitations of certificate-based verification are discussed, but simple field-based temporary marking approaches (e.g. livestock paint markings), which are inexpensive and operationally practical in mass dog vaccination campaigns, should at least be acknowledged. Further, the manuscript should more fully acknowledge the limitations of certificate-based vaccination verification.

Response: As noted, the limitations of certificates have been discussed in a dedicated sub-section of the Discussion. This section has been augmented with further discussion regarding the limitations of this approach, plus discussion of the mark-resight method mentioned by the reviewer (lines 847-84).

Point 4: Presentation of preliminary comparative operational cost data: the current manuscript makes broader operational and scalability claims that are difficult to fully evaluate without at least preliminary comparative cost information. It is therefore challenging for the reader to assess whether the higher coverage achieved through the community-based approach was obtained more efficiently, more economically, or simply through greater operational input over time.

Response: We appreciate this point of view and have reviewed the sections where statements about scalability have been made and we have nuanced the text to reflect this caution (lines 104 (Author Summary), 676-704 (Discussion) and 887-94 (Conclusion). In addition, the cost-effectiveness data has now been published and is therefore available for consideration(1).

Point 5: Broader claims regarding scalability and suitability for national elimination strategies should be more cautiously framed until supported by accompanying economic analyses, surveillance outcomes, and evaluation across a wider range of epidemiological and operational settings.

Response: Like Point 4, we consider this a fair critique and have reviewed the sections where statements about scalability have been made and we have nuanced the text to reflect this caution (lines 104 (Author Summary), 676-704 (Discussion) and 887-94 (Conclusion).

Point 6: While vaccination coverage is an entirely appropriate primary endpoint, the manuscript should remain cautious in linking these findings to elimination impact without accompanying surveillance outcomes. The distinction between improved vaccination performance and demonstrated reduction in rabies transmission should remain explicit and carefully maintained throughout.

Response: While the link between increasing mass dog vaccination coverage and reduced incidence of dog bites from suspect rabid dogs and human rabies cases has been widely reported in the literature, we accept this point and have cautiously described the possible link between achieving consistent vaccination coverage and impacts that this ‘could’ have on national elimination strategies and on controlling transmission (lines 75 (Abstract), 104 (Author Summary), 676-704 (Discussion)).

Point 7: Minor editorial tightening of the Discussion would improve readability by reducing repetition around thermotolerance and community engagement concepts.

Response: We have reviewed the two sections in the Discussion (Community based delivery and Thermotolerance) and have edited to remove repetition regarding thermotolerance and community engagement concepts (lines 707-782).

Point 8: The manuscript would benefit from inclusion of a brief operational comparison table summarising the practical differences between the two delivery strategies, including personnel structure, vaccination frequency, storage approach, and follow-up activities.

Response: This is a good suggestion, and we have added an operations table to the manuscript (see Table 1).

Point 9: Minor improvements to supplementary figure labelling and simplification of some statistical presentation would aid rapid interpretation

Response: We have reviewed the statistical presentation and the supplementary figure labelling and have made minor edits where necessary to address some typographical errors.

Point 10: Both reviewers comment on the lack of cost comparison data and the impact that this might have on interpretation of comparative programme value. Further, Reviewer Two comments that we state that both delivery approaches could be implemented by local authorities, yet the reader cannot validate this claim if the resources (cost) necessary to operate these two vaccination approaches are not clearly stated within this manuscript.

Response: We would like to state that the focus of this paper is to describe the primary and secondary measurable outputs of the RCT, which both focus on coverage. As mentioned, the cost-effectiveness paper has now been published (1) and the paper reporting the epidemiological data is forthcoming. Whilst providing the coverage, public health and cost data in a single publication would have had merit, it would have made this paper extremely long, would compromise our ability to describe the separate analyses in detail, and would take the paper beyond the size for most journals. Further, publishing the primary outcomes of an RCT without the economic data has precedence (2–6). The referenced publications are a selection taken from respected journals in which RCTs have been reported in which the primary outcome measure was vaccination coverage and where cost effectiveness analyses were not reported. In some of these papers, cost-analyses will have been reported separately as we are in the process of doing. Indeed, in a systematic review article that aimed to collate data on economic evaluations of interventions to increase vaccine uptake across a range of settings and vaccine types, of 365 papers reviewed 333 were found to report coverage findings only, without the inclusion of economic data (7).

Additionally, we have strived to make it clear from the detailed descriptions given that the two approaches differ in a relatively straightforward way: that is, Team-based approach only allows vaccination within each village once per year. Whilst the Community-based approach allowed vaccination to be implemented throughout the year. Moreover, we described in the Introduction (lines 168-78) that the pilot study allowed the resources required to implement the strategies to be estimated so that programmatic costs, such as for personnel time, travel and communication, could be quantified with the aim that that sufficient and comparable resources would be provided for each strategy, and that, following the establishment of these comparable resource input levels, the research team would not specify resource constraints differentially in favor of one arm or the other. Therefore, we think that the reader will be able to appreciate the methodological difference between the two strategies and will find the coverage differences achieved informative and, even with the cost data published in another paper, potentially actionable.

In summary, this paper is not an evaluation of the economic effectiveness or the public health impacts of the delivery strategies.  Instead, it is an assessment of the primary delivery outcomes (coverage) of the RCT.  The economic assessment and public health impacts required dedicated papers that allow the analyses of these components to be described in appropriate detail. As mentioned, the cost-effectiveness paper has been published and the public health paper will follow.

Regarding the comment about local authorities requiring cost data to be fully informed regarding the economic feasibility of the Community-based approach, this is a reasonable point and we had existing, and have now added new, text that explains that the trial results are focused on impacts on coverage and that extrapolation to feasibility and conclusions about public health benefits, cost effectiveness and feasibility of implementation can only be drawn when both the health and economic analyses are published, which is happening separately (lines 102, 685-704, 748-762, 887-894).

Whilst the cost-data has now been published in a separate paper, we would like to draw attention to the comment from Reviewer One who states that the objectives of the study are clearly articulated, and the hypothesis (regarding coverage) is clear and testable and that the study addresses an important operational question relevant to rabies elimination strategies in endemic settings. We agree with this comment and believe that all the remaining issues raised by the reviewers have been addressed.

Point 11: Reviewer 2 stated that ‘I cannot support publication of a paper where the data necessary to evaluate the impact of the study design is being withheld.’

Response: We are not withholding data but were unable to include the full scope of the work within a single paper. In the discussion we now point to the published cost data and the epidemiological data are forthcoming.

1.	Changalucha JJ, Anderson D, Yoder J, Stone B, Lugelo A, Kimera SI, Lupindu AM, Hampson K, Lankester F. Evaluating delivery models for mass dog vaccination: A cost-effectiveness analysis of community-led and team-led vaccination strategies for rabies control in resource-limited settings. Front Trop Dis Trop Dis. 2026;6(1725443).

2.	Asante KP, Mathanga DP, Milligan P, Akech S, Oduro A, Mwapasa V, et al. Feasibility , safety , and impact of the RTS , S / AS01 E malaria vaccine when implemented through national immunisation programmes : evaluation of cluster-randomised introduction of the vaccine in Ghana, Kenya, and Malawi. Lancet. 2024;403.

3.	Oyo-ita A, Bosch-capblanch X, Ross A, Oku A, Id EE, Ameh S, Oduwole O, Id DA, Meremikwu M. Effects of engaging communities in decision- making and action through traditional and religious leaders on vaccination coverage in Cross River State, Nigeria: A cluster- randomised control trial. PLoS One [Internet]. 2021;16(4):1–14. Available from: http://dx.doi.org/10.1371/journal.pone.0248236

4.	Gibson DG, Ochieng B, Kagucia EW, Were J, Hayford K, Moulton LH, Levine OS, Odhiambo F, Brien KLO, Feikin DR. Articles Mobile phone-delivered reminders and incentives to improve childhood immunisation coverage and timeliness in Kenya (M-SIMU): a cluster randomised controlled trial. Lancet Glob Heal [Internet]. 2017;5(4):e428–38. Available from: http://dx.doi.org/10.1016/S2214-109X(17)30072-4

5.	Omale UI, Ewah RL, Amuzie CI, Ikegwuonu CO, Nkwo GE, Iwegbulam CC, Ekwuazi LC. Study protocol for a cluster-randomised controlled trial of hybrid parents and health workers adaptive intervention for optimal routine childhood immunisation coverage in the communities of Ebonyi state, Nigeria: the AGINTOPIC trial protocol. BMJJ Open. 2023;13(e068953):1–16.

6.	Omale UI, Ewah RL, Amuzie CI, Ikegwuonu CO, Nkwo GE, Iwegbulam CC, Ekwuazi LC, Health G. A pragmatic covariate-constrained randomised controlled trial of hybrid parents and health workers adaptive intervention for optimal (timely , cumulative age-appropriate) wide routine childhood immunisation coverage: the AGINTOPIC trial. BMJ Glob Heal. 2025;10(e017403):1–21.

7.	Hamashima Y, Breheny K, Davies SR, Dawson S, Thornton ZA, Aiton E, Caldwell DM, Christensen H, Higgins JPT, Yates J, Letley L, French CE. A synthesis of economic data from randomized trials of recipient-focused interventions to increase vaccine uptake. Vaccine [Internet]. 2025;68(127906). Available from: https://doi.org/10.1016/j.vaccine.2025.127906

**Full text of reviewers’ comments:**

Reviewer #1: The objectives of the study are clearly articulated, and the hypothesis - that vaccination coverage differs between community-based continuous delivery and annual team-based delivery - is clear and testable. The study addresses an important operational question relevant to rabies elimination strategies in endemic settings.

The cluster-randomised design is generally appropriate, and the 'fried-egg' approach to minimise contamination between intervention arms is clever and well justified. The study population is clearly described and appropriate for assessing vaccination delivery strategies in predominantly rural endemic regions of sub-Saharan Africa.

However, applicability beyond similar agro-ecological and operational settings should continue to be framed cautiously, particularly in larger urban environments, pastoral systems, or regions with more complex cross-border dog movement dynamics.

The sample size appears appropriate and adequately powered for the primary endpoint of vaccination coverage. The statistical analysis looks rigorous and appropriately conducted, with useful sensitivity analyses included. Additional clarification regarding assumptions underlying the modelling of minimum initial coverage and the probability of falling below the 40% threshold has improved the manuscript.
However, there remain important methodological concerns regarding the interpretation of the comparator arm. The manuscript increasingly frames the community-based approach as operationally superior, yet the team-based intervention described does not appear representative of many highly effective team-based rabies vaccination campaigns currently operating in Africa and Asia.

Numerous programmes routinely achieve substantially higher coverage using static-point and team-based approaches without extensive infrastructure, refrigeration access, or high-cost operational models. The description implying that standard team-based approaches depend primarily on electricity, refrigeration hubs, and centrally based vaccinators doesn't fully reflect operational realities in many successful field programmes.

Similarly, the team structure employed in the comparator arm appears operationally limited relative to best-practice mass dog vaccination delivery and may therefore underestimate the achievable effectiveness of well-organised team-based campaigns under comparable resource-constrained conditions. This requires more balanced discussion and clearer qualification throughout the manuscript.

An additional methodological limitation relates to vaccination verification. The manuscript relies heavily on vaccination certificates to determine coverage estimates. While the sensitivity analysis is useful, the Methods should better justify why alternative field-based identification approaches were not incorporated.

In many large-scale rabies vaccination campaigns, temporary paint marking is used as a rapid, practical, highly economical, and operationally scalable method for identifying vaccinated dogs during post-vaccination surveys and transects. Such approaches avoid reliance on owner-retained documentation, which may introduce variability associated with certificate loss or inconsistent presentation. The authors should explain whether temporary marking methods were considered and why they were not included in the study design.

Finally, although the manuscript discusses operational efficiency and potential scalability of the intervention, the absence of accompanying cost-effectiveness and surveillance outcome data substantially limits interpretation of comparative programme value. Given that broader implementation claims are now made within the Discussion, presentation of at least preliminary comparative operational cost data within the current manuscript would considerably strengthen interpretation of the findings.

Reviewer #2: (No Response)

**Results**


-Does the analysis presented match the analysis plan?

-Are the results clearly and completely presented?

-Are the figures (Tables, Images) of sufficient quality for clarity?

Reviewer #1: The analyses presented broadly match the stated analysis plan and are generally reported clearly. The results are logically structured, and the primary finding - that the community-based arm achieved higher and more stable vaccination coverage over time - is consistently demonstrated across the analyses presented.

The inclusion of sensitivity analyses using stricter vaccination definitions strengthens the robustness of the findings, and the additional clarification around modelling assumptions has improved interpretability. The presentation of temporal variation and the probability of coverage falling below the 40% threshold is particularly useful in illustrating differences in stability between intervention arms.

Figures and tables are generally of good quality and support interpretation effectively. The graphical presentation of coverage trends over time and threshold probabilities is clear and accessible. Minor improvements to supplementary figure labelling and simplification of some statistical presentation would probably further aid rapid interpretation by readers.

However, important concerns remain regarding interpretation of the comparator arm and the operational conclusions drawn from the data. The relatively modest coverage achieved in the team-based arm appears substantially lower than that achieved by many established mass dog vaccination programmes operating in Africa and Asia using team-based and static-point approaches. As a result, it remains difficult to determine to what extent the observed differences reflect genuine superiority of the community-based strategy versus limitations in the implementation of the comparator intervention itself.

Related to this, the manuscript still does not provide comparative operational cost data or epidemiological surveillance outcomes. While the authors indicate these analyses will be published separately, the current manuscript makes broader operational and scalability claims that are difficult to fully evaluate without at least preliminary comparative cost information. It is therefore challenging for the reader to assess whether the higher coverage achieved through the community-based approach was obtained more efficiently, more economically, or simply through greater operational input over time.

There also remains concern regarding vaccination verification methodology. Reliance on vaccination certificates alone may introduce variability associated with certificate retention and presentation. The manuscript would benefit from greater discussion of practical alternative field approaches, such as temporary paint marking, which are widely used in rabies vaccination campaigns because they are rapid, inexpensive, and operationally scalable for post-vaccination assessment.

Overall, the results are important and potentially highly relevant to rabies elimination strategies. However, stronger qualification of the comparator limitations and inclusion of additional operational context are necessary to avoid over-interpretation of comparative effectiveness claims.

Reviewer #2: (No Response)

**Conclusions**


-Are the conclusions supported by the data presented?

-Are the limitations of analysis clearly described?

-Do the authors discuss how these data can be helpful to advance our understanding of the topic under study?

-Is public health relevance addressed?

Reviewer #1: The conclusions are partially supported by the data presented. The study clearly demonstrates that, within the operational framework tested, the community-based approach achieved higher and more stable vaccination coverage over time than the comparator team-based approach. The data also support the finding that coverage in the community-based arm was less likely to fall below the theoretical critical threshold during the annual cycle.

However, the manuscript at times moves beyond the evidence directly generated by the study. The trial demonstrates differences in vaccination coverage performance, but it does not directly demonstrate rabies elimination, reduced rabies transmission, reductions in human exposure, or improved public health outcomes. These distinctions should remain clearly separated throughout the Discussion and Conclusions to avoid overinterpretation.

Although additional limitations have now been acknowledged, some important limitations still require stronger emphasis. In particular:

1. The comparator team-based intervention may not fully represent the performance achievable by well-organised team-based rabies vaccination programmes operating elsewhere in Africa and Asia.

2. The study was conducted within predominantly rural endemic settings in northern Tanzania, limiting generalisability beyond similar sub-Saharan African contexts.

3. vaccination verification relied heavily on certificates rather than alternative field-identification approaches such as temporary paint marking.

4. epidemiological surveillance outcomes and comparative cost-effectiveness data are not presented within the current manuscript.

The absence of comparative operational cost data is especially important because the manuscript increasingly discusses scalability, sustainability, and programmatic superiority of the intervention. Without accompanying cost analyses, it remains difficult to fully evaluate whether the observed gains in coverage were achieved more efficiently or simply through greater operational input over time.

This said, the manuscript nevertheless makes an important contribution to implementation science and operational rabies control. It addresses one of the major practical challenges in rabies elimination programmes: maintaining vaccination coverage above critical thresholds in remote and resource-constrained settings. The findings are therefore highly relevant to ongoing discussions around delivery optimisation for endemic regions.

Public health relevance is clearly addressed and the topic is timely and important given global 'Zero by 30' ambitions. However, broader claims regarding scalability and suitability for national elimination strategies should be more cautiously framed until supported by accompanying economic analyses, surveillance outcomes, and evaluation across a wider range of epidemiological and operational settings.

Reviewer #2: (No Response)

**Editorial and Data Presentation Modifications?**


Use this section for editorial suggestions as well as relatively minor modifications of existing data that would enhance clarity. If the only modifications needed are minor and/or editorial, you may wish to recommend “Minor Revision” or “Accept”.

Reviewer #1: The manuscript is generally well written and substantially improved from the original submission. The figures and tables are clear and support interpretation effectively. However, several editorial and presentation issues should still be addressed to improve balance, clarity, and interpretability:

1. The wording describing “standard” team-based delivery should be moderated. Some statements imply that cold-chain dependence, limited infrastructure, and low coverage are universally representative of team-based rabies vaccination programmes in Africa. This is not consistently true, including in large-scale field programmes operating successfully in remote settings without fixed refrigeration infrastructure.

2. The Discussion should more clearly distinguish between the specific comparator tested in this trial and broader team-based approaches used elsewhere in Africa and Asia.

3. Greater caution should be used when discussing scalability and superiority of the community-based model in the absence of comparative cost-effectiveness and surveillance outcome data within the current manuscript.

4. The manuscript would benefit from inclusion of a brief operational comparison table summarising the practical differences between the two delivery strategies, including personnel structure, vaccination frequency, storage approach, and follow-up activities.

5. Additional clarification should be included regarding how vaccinated dogs were identified during post-vaccination assessment. The limitations of certificate-based verification are discussed, but simple field-based temporary marking approaches (e.g. livestock paint markings), which are inexpensive and operationally practical in mass dog vaccination campaigns, should at least be acknowledged.

6. Some supplementary figures and tables would benefit from clearer labelling to improve rapid interpretation by readers unfamiliar with the study design.

7. Minor editorial tightening of the Discussion would improve readability by reducing repetition around thermotolerance and community engagement concepts.

Reviewer #2: (No Response)

**Summary and General Comments**


Use this section to provide overall comments, discuss strengths/weaknesses of the study, novelty, significance, general execution and scholarship. You may also include additional comments for the author, including concerns about dual publication, research ethics, or publication ethics. If requesting major revision, please articulate the new experiments that are needed.

Reviewer #1: This is an important, ambitious and operationally highly relevant three-year cluster randomised controlled trial addressing one of the central challenges in canine rabies elimination: maintaining vaccination coverage above critical herd immunity thresholds in endemic settings. The manuscript is clearly written, statistically sophisticated, and tackles a genuinely important implementation problem with substantial potential public health relevance.

The revised manuscript is substantially improved and many previous concerns have been addressed thoughtfully. However, following review of the revised text and author responses, I consider that several remaining issues materially affect interpretation of the findings and the strength of the conclusions being drawn. For this reason, I now believe Major Revision is more appropriate prior to acceptance.

The principal concern relates to the framing and representativeness of the comparator 'team-based' arm. The manuscript repeatedly presents the comparator as representative of standard team-based rabies vaccination delivery across Africa. However, many successful large-scale rabies control programmes operating in Africa - including highly resource-efficient field programmes working in remote areas without fixed refrigeration infrastructure - routinely achieve substantially higher coverage using team-based static-point approaches than those reported here. As currently framed, the manuscript risks implying that the relatively low coverage observed in the comparator arm is inherent to team-based delivery itself, rather than reflecting the specific implementation model adopted within this trial. This distinction is important and should be addressed more carefully and neutrally throughout the manuscript.

Related to this, several statements discussing logistical limitations of team-based delivery appear overly generalised, particularly around dependency on fixed electricity and refrigeration infrastructure. In practice, many programmes operate effectively using passive cooling systems, temporary cold-chain management, mobile vaccination teams and highly decentralised operational structures. The current wording risks overstating the novelty or operational advantage of the intervention by underrepresenting the flexibility and effectiveness of alternative field delivery approaches already used successfully at scale.

The study convincingly demonstrates improved and more stable vaccination coverage under the tested community-based delivery model. However, the manuscript occasionally moves beyond the data in suggesting implications for elimination impact, scalability and superiority of delivery strategy without simultaneously presenting the associated comparative operational costs and epidemiological outcomes. The absence of integrated cost-effectiveness analysis is a significant limitation because the practical value of any delivery model depends not only on achieved coverage but on the resources, personnel structure, supervision requirements and sustainability needed to achieve it. While I appreciate that economic analyses are planned separately, the current manuscript makes interpretive claims that are difficult to fully evaluate in the absence of at least comparative operational cost summaries within the same paper.

Similarly, while vaccination coverage is an entirely appropriate primary endpoint, the manuscript should remain cautious in linking these findings to elimination impact without accompanying surveillance outcomes. The distinction between improved vaccination performance and demonstrated reduction in rabies transmission should remain explicit and carefully maintained throughout.

I also believe the manuscript should more fully acknowledge the limitations of certificate-based vaccination verification. The discussion appropriately notes limitations of collars, microchips and facial recognition, but practical field marking methods such as temporary livestock paint markings - widely used in mass vaccination campaigns because they are rapid, inexpensive and operationally simple for post-vaccination assessment - are not discussed and should at least be acknowledged as highly effective alternative operational approaches.

Finally, applicability beyond the specific epidemiological and operational context studied should remain carefully qualified. The intervention appears particularly suited to decentralised rural endemic settings typical of parts of sub-Saharan Africa. Generalisation to dense urban settings, highly mobile dog populations, or different sociocultural contexts requires further evidence.

Overall, this remains a valuable and potentially influential contribution to rabies implementation science. However, I believe the framing, contextualisation, and interpretation of the findings require further revision before the conclusions can be fully supported at the level currently presented.

Reviewer #2: I cannot support publication of a paper where the data necessary to evaluate the impact of the study design is being withheld; to be published separately. It sounds like the authors have all of the cost and surveillance data necessary to demonstrate the feasibility (cost) and effectiveness (surveillance) of these two approaches, but have chosen not to include that critical information.

The authors repeat a statement that both approaches could be implemented by local authorities. The reader cannot validate that claim if the resources (cost) necessary to operate these two vaccination approaches are not clearly stated within this manuscript.

PLOS authors have the option to publish the peer review history of their article ([what does this mean?](https://track.editorialmanager.com/CL0/https:%2F%2Fjournals.plos.org%2Fplosntds%2Fs%2Feditorial-and-peer-review-process%23loc-peer-review-history/1/010f019e687f4ed5-264fee96-542d-4e4f-bb15-40b52bd92185-000000/YIwQnLaKBsRnuzm2GAorq4Kq8x8B0S49lftAa7sZ2A0=258)). If published, this will include your full peer review and any attached files.


If you choose “no”, your identity will remain anonymous but your review may still be made public.


**Do you want your identity to be public for this peer review?** For information about this choice, including consent withdrawal, please see our [Privacy Policy](https://track.editorialmanager.com/CL0/https:%2F%2Fwww.plos.org%2Fprivacy-policy/1/010f019e687f4ed5-264fee96-542d-4e4f-bb15-40b52bd92185-000000/N0KKTN8LCnwHV9Tj86jVXzFVymWfmjC-49ILpcEAQ80=258).

Reviewer #1: No

Reviewer #2: No

[NOTE: If reviewer comments were submitted as an attachment file, they will be attached to this email and accessible via the submission site. Please log into your account, locate the manuscript record, and check for the action link "View Attachments". If this link does not appear, there are no attachment files.]

**Figure resubmission:**

While revising your submission, we strongly recommend that you use PLOS’s NAAS tool (<https://ngplosjournals.pagemajik.ai/artanalysis>) to test your figure files. NAAS can convert your figure files to the TIFF file type and meet basic requirements (such as print size, resolution), or provide you with a report on issues that do not meet our requirements and that NAAS cannot fix.

After uploading your figures to PLOS’s NAAS tool - <https://ngplosjournals.pagemajik.ai/artanalysis>, NAAS will process the files provided and display the results in the "Uploaded Files" section of the page as the processing is complete. If the uploaded figures meet our requirements (or NAAS is able to fix the files to meet our requirements), the figure will be marked as "fixed" above. If NAAS is unable to fix the files, a red "failed" label will appear above. When NAAS has confirmed that the figure files meet our requirements, please download the file via the download option, and include these NAAS processed figure files when submitting your revised manuscript.

**Reproducibility:**

To enhance the reproducibility of your results, we recommend that authors of applicable studies deposit laboratory protocols in protocols.io, where a protocol can be assigned its own identifier (DOI) such that it can be cited independently in the future. Additionally, PLOS ONE offers an option to publish peer-reviewed clinical study protocols. Read more information on sharing protocols at <https://plos.org/protocols?utm_medium=editorial-email&utm_source=authorletters&utm_campaign=protocols>
