# Supplementary material for: Effectiveness of community-based delivery of mass dog vaccination to prevent rabies: A cluster randomized controlled trial
Source: PLoS Negl Trop Dis. 2026 Sep 25;20(9):e0014704. doi: 10.1371/journal.pntd.0014704 (PMC13641700; doi:10.1371/journal.pntd.0014704)
Supplement: Supplementary file 13 [file pntd.0014704.s017.docx]

Response document

We thank the reviewer for taking time to review our manuscript once again. We are pleased that the majority of concerns were addressed and have made every effort to address these final concerns below.

Reviewer #1: The revised manuscript has addressed the majority of my previous methodological concerns. One minor point remains regarding the comparator. The team-based arm represents a specific government delivery model rather than the full spectrum of contemporary team-based mass dog vaccination programmes. I encourage the authors to make this distinction explicit, as well-managed field team approaches have achieved substantially higher post-campaign coverage in other endemic settings.

Response: In the previous rounds of revisions, we made several changes, detailed in the previous response to reviewer documents, regarding Team-based delivery representing the delivery implemented by governments, and that this was different to NGO based delivery. The following text has been taken from the previous response to reviewer document:

*For example, in lines 163-6) of the Introduction we are clear that the tested Team-based approach was designed to reflect typical team-based vaccination strategies employed by governments, such as the Government of the Republic of Tanzania, to implement mass dog vaccination. Further, in the Discussion (lines 863-78), we dedicated a sub-section (Team-based arm) to discuss the fact that the coverage achieved in the Team-based arm of the trial was relatively modest and that this contrasts with the coverage achieved by team-based mass dog vaccination campaigns implemented by NGOs in Africa and Asia where coverage in excess of a target of 70% can be achieved. However, such high levels of coverage is not typical of government-led programs and, as such, this text is followed by a detailed explanation as to why team-based mass dog vaccination implemented by non-governmental organisations might be more effective at reaching higher levels of coverage.*

*Despite this, we have further emphasised this with an additional sentence in lines 163-6 that states that the Team-based approach was not designed to reflect the approach commonly used by non-governmental organisations that often have the resources needed to implement post-vaccination coverage assessments and targeted follow-up vaccination efforts. Additionally, where appropriate, we have removed the word ‘standard’ as a descriptor of the Team-based approach.*

As such, we feel we have addressed this issue sufficiently, and that the readers will fully understand this distinction and that further caveats are unnecessary and will detract from the paper. Nonetheless, we have made additional clarifying edits in the Abstract (line 62) and in the Author Summary (line 101).

Reviewer #1- Results: The analyses presented are consistent with the analysis plan. The results are clearly presented and the figures and tables are of good quality. I have no major concerns with the presentation of the results.

Response: We are grateful for the reviewer’s approval of the results.

Reviewer #1- Conclusion: The conclusions are supported by the data presented and are generally appropriately balanced. The manuscript now more clearly acknowledges the principal limitations of the study, and the discussion highlights the contribution of the findings to improving mass dog vaccination delivery in endemic settings. Public health relevance is clear and well articulated. I would encourage one final clarification that the findings demonstrate improved vaccination coverage within the study setting rather than direct evidence of rabies elimination, and that applicability beyond similar decentralised rural systems should be interpreted with appropriate caution.

Response: We are pleased that the reviewer found the conclusions to be supported by the data and that the manuscript appropriately describes the limitations of the trial, the contribution of the findings to improving mass dog vaccination, and that the public health relevance is clear and well-articulated. Regarding the suggestion that further clarification is needed regarding applicability beyond similar rural systems, we would like to point out that we dealt with this suggestion comprehensively in the last two rounds of review. I draw attention to the text provided in the previous response to reviewer document which details the point made by the reviewer and our response:

*Point 1: Applicability beyond the specific epidemiological and operational context studied should remain carefully qualified. The intervention appears particularly suited to decentralised rural endemic settings typical of parts of sub-Saharan Africa. Generalisation to dense urban settings, highly mobile dog populations, or different sociocultural contexts requires further evidence.*

*Response: This is an important point and is one that we addressed in the last round of revisions. Please see the Impact of Geography sub-section of the Discussion (lines 819-61) where we discuss in detail that the trial results emanate from a predominantly rural region dominated by small holder agro-pastoral communities with relatively small interspersed urban areas. We state that the effectiveness of this approach may vary in larger urban areas or pastoral settings, where differences in dog ownership patterns and human population densities could influence community vaccinators' ability to reach dogs. We continue that to achieve widespread rabies control across endemic countries such as Tanzania, composed of mosaics of agro-ecological areas and landscapes, comprising varied human and domestic animal densities, control efforts will likely require locally tailored mass vaccination approaches that exploit different delivery strategies. Further, we caution against drawing conclusions regarding the suitability of the Community-based intervention in rabies endemic countries that do not share these agro-ecological similarities, for example in countries in Asia where landscapes and domestic dog demographics can be very different. Consequently, we feel that we have sufficiently addressed this point. However, we have added a clause to the abstract and the author summary to make sure that this important point is understood in these summarised sections (lines 73 and 100-2).*

Regarding the suggestion that we clarify that the findings demonstrate improved vaccination coverage within the study setting rather than direct evidence of rabies elimination, again we did deal with this in the previous review. We have copied the relevant text from the response to reviewer document below:

*Point 6: While vaccination coverage is an entirely appropriate primary endpoint, the manuscript should remain cautious in linking these findings to elimination impact without accompanying surveillance outcomes. The distinction between improved vaccination performance and demonstrated reduction in rabies transmission should remain explicit and carefully maintained throughout.*

*Response: While the link between increasing mass dog vaccination coverage and reduced incidence of dog bites from suspect rabid dogs and human rabies cases has been widely reported in the literature, we accept this point and have cautiously described the possible link between achieving consistent vaccination coverage and impacts that this ‘could’ have on national elimination strategies and on controlling transmission (lines 75 (Abstract), 104 (Author Summary), 676-704 (Discussion)).*

The links between mass dog vaccination and rabies outcomes are widely reported in the literature. Further, we believe that, despite not reporting surveillance outcomes in this paper, the link between mass dog vaccination coverage and rabies outcomes is sufficiently clear that readers will not expect further caveats regarding this point. Nonetheless, we added text to emphasise this point in the last review and have re-reviewed these edits and have strengthened the text in lines 78 and lines 106-8.

Reviewer #1 - Editorial suggestions: The manuscript remains well written and clearly presented. The figures and tables are of good quality and support interpretation of the findings. I encourage the authors to make it explicit that the comparator represents a specific government team-based delivery model rather than the full spectrum of contemporary team-based mass dog vaccination programmes, to avoid over-generalisation of the findings. Additionally, the discussion should continue to emphasise that the results are most directly applicable to decentralised rural endemic settings typical of sub-Saharan Africa, and that extrapolation to other epidemiological or operational contexts should be made cautiously. Finally, a brief clarification of the assumptions underpinning the estimation of the minimum initial vaccination coverage required to maintain coverage above the 40% threshold would further improve clarity.

Response: We thank the reviewer for the complements regarding the writing and presentation of the manuscript. Regarding the comments of the government team-based approach and the rural setting, these have been addressed above. Regarding the comment about the assumption underpinning the estimation of the minimum vaccination coverage, we have reviewed this text and further explanatory text has been added in lines 51, and a reference citation and a brief description of the analysis underpinning this estimate was added to line 137-9. Further, a detailed discussion of the critical vaccination threshold is already in place in lines 663-674.

Reviewer #1- Summary: This remains an important and well-executed cluster randomised controlled trial addressing one of the key operational challenges in canine rabies elimination: how to achieve and maintain effective vaccination coverage in endemic settings. The manuscript is well written, the study design is robust, and I found the operational insights both interesting and highly relevant.
The authors have responded constructively to the previous review comments, and the manuscript has been strengthened as a result. In particular, the discussion now provides a more balanced interpretation of the findings and better acknowledges the study's limitations.
My remaining comments are minor and relate primarily to framing rather than methodology. I encourage the authors to continue making it explicit that the comparator represents a specific government team-based delivery model rather than the full spectrum of contemporary team-based mass dog vaccination approaches. Likewise, while the findings provide strong evidence that the community-based approach improves and maintains vaccination coverage, they should continue to distinguish this from direct evidence of rabies elimination and emphasise that the findings are most directly applicable to decentralised rural endemic settings typical of sub-Saharan Africa.
Overall, I believe this manuscript makes a valuable contribution to the evidence base for rabies control and implementation science. Subject to these minor clarifications, I support publication.

Response: We thank the reviewer for these constructive comments. We have dealt with all these comments in the responses above. Once again, we would like to thank the reviewer for the considerable amount of time that they have given reviewing the manuscript on multiple occasions. Their inputs have allowed us to improve the paper.
