# Supplementary material for: Effectiveness of community-based delivery of mass dog vaccination to prevent rabies: A cluster randomized controlled trial
Source: PLoS Negl Trop Dis. 2026 Sep 25;20(9):e0014704. doi: 10.1371/journal.pntd.0014704 (PMC13641700; doi:10.1371/journal.pntd.0014704)
Supplement: Peer review history

# Author comment: Author response to Decision Letter 0

**DOI:** 10.1371/journal.pntd.0014704.r001

**Transfer Alert**

This paper was transferred from another journal. As a result, its full editorial history (including decision letters, peer reviews and author responses) may not be present.

2 Feb 2026

# Peer review: Decision Letter 0

**DOI:** 10.1371/journal.pntd.0014704.r002

**Authors:** Annapaola Rizzoli, Philip P. Mshelbwala

12 Mar 2026

PNTD-D-26-00213

Effectiveness of Community-based delivery of mass dog vaccination to prevent rabies: A cluster randomized controlled trial

PLOS Neglected Tropical Diseases

Dear Dr. Lankester,

Thank you for submitting your manuscript to PLOS Neglected Tropical Diseases. After careful consideration, we feel that it has merit but does not fully meet PLOS Neglected Tropical Diseases's publication criteria as it currently stands. Therefore, we invite you to submit a revised version of the manuscript that addresses the points raised during the review process.

Please submit your revised manuscript within by 11th May 2026. If you will need more time than this to complete your revisions, please reply to this message or contact the journal office at plosntds@plos.org. When you're ready to submit your revision, log on to https://www.editorialmanager.com/pntd/ and select the 'Submissions Needing Revision' folder to locate your manuscript file.

Please include the following items when submitting your revised manuscript:

* A letter that responds to each point raised by the editor and reviewer(s). You should upload this letter as a separate file labeled 'Response to Reviewers'. This file does not need to include responses to any formatting updates and technical items listed in the 'Journal Requirements' section below.

* A marked-up copy of your manuscript that highlights changes made to the original version. You should upload this as a separate file labeled 'Revised Manuscript with Track Changes'.

* An unmarked version of your revised paper without tracked changes. You should upload this as a separate file labeled 'Manuscript'.

If you would like to make changes to your financial disclosure, competing interests statement, or data availability statement, please make these updates within the submission form at the time of resubmission. Guidelines for resubmitting your figure files are available below the reviewer comments at the end of this letter.

We look forward to receiving your revised manuscript.

Kind regards,

Philip P. Mshelbwala

Academic Editor

PLOS Neglected Tropical Diseases

Annapaola Rizzoli

Section Editor

PLOS Neglected Tropical Diseases

Shaden Kamhawi

co-Editor-in-Chief

PLOS Neglected Tropical Diseases

orcid.org/0000-0003-4304-636XX

Paul Brindley

co-Editor-in-Chief

PLOS Neglected Tropical Diseases

orcid.org/0000-0003-1765-0002

**Journal Requirements:**

If the reviewer comments include a recommendation to cite specific previously published works, please review and evaluate these publications to determine whether they are relevant and should be cited. There is no requirement to cite these works unless the editor has indicated otherwise.

1) Please ensure that the CRediT author contributions listed for every co-author are completed accurately and in full.

At this stage, the following Authors/Authors require contributions: Ahmed Lugelo, Joel Changalucha, Danni Anderson, Christian Tetteh Duamor, Anna Czupryna, Kennedy Lushasi, Elaine Ferguson, Emmanuel Swai, Maganga Sambo, Jonathan Yoder, Sarah Cleaveland, Sally Wyke, Benezeth Lutege Malinda, Paul C. D. Johnson, and Katie Hampson. Please ensure that the full contributions of each author are acknowledged in the "Add/Edit/Remove Authors" section of our submission form.

The list of CRediT author contributions may be found here: https://journals.plos.org/plosntds/s/authorship#loc-author-contributions

2) Please provide an Author Summary. This should appear in your manuscript between the Abstract (if applicable) and the Introduction, and should be 150-200 words long. The aim should be to make your findings accessible to a wide audience that includes both scientists and non-scientists. Sample summaries can be found on our website under Submission Guidelines:

https://journals.plos.org/plosntds/s/submission-guidelines#loc-parts-of-a-submission

3) We noticed that you used the phrase 'unpublished data' in the manuscript. We do not allow these references, as the PLOS data access policy requires that all data be either published with the manuscript or made available in a publicly accessible database. Please amend the supplementary material to include the referenced data or remove the references.

4) We do not publish any copyright or trademark symbols that usually accompany proprietary names, eg ©,  ®, or TM  (e.g. next to drug or reagent names). Therefore please remove all instances of trademark/copyright symbols throughout the text, including:

- TM on pages: 9, 10, 33, 34, 35, and 36.

5) Thank you for including an Ethics Statement for your study. Please include:

i) A statement that formal consent was obtained (must state whether verbal/written) OR the reason consent was not obtained (e.g. anonymity). NOTE: If child participants, the statement must declare that formal consent was obtained from the parent/guardian.].

6) Please upload all main figures as separate Figure files in .tif or .eps format. For more information about how to convert and format your figure files please see our guidelines:

https://journals.plos.org/plosntds/s/figures

7) We notice that your supplementary Figures, and Tables are included in the manuscript file. Please remove them and upload them with the file type 'Supporting Information'. Please ensure that each Supporting Information file has a legend listed in the manuscript after the references list.

8) Some material included in your submission may be copyrighted. According to PLOSu2019s copyright policy, authors who use figures or other material (e.g., graphics, clipart, maps) from another author or copyright holder must demonstrate or obtain permission to publish this material under the Creative Commons Attribution 4.0 International (CC BY 4.0) License used by PLOS journals. Please closely review the details of PLOSu2019s copyright requirements here: PLOS Licenses and Copyright. If you need to request permissions from a copyright holder, you may use PLOS's Copyright Content Permission form.

Please respond directly to this email and provide any known details concerning your material's license terms and permissions required for reuse, even if you have not yet obtained copyright permissions or are unsure of your material's copyright compatibility. Once you have responded and addressed all other outstanding technical requirements, you may resubmit your manuscript within Editorial Manager.

Potential Copyright Issues:

i) Figure 1. Please (a) provide a direct link to the base layer of the map (i.e., the country or region border shape) and ensure this is also included in the figure legend; and (b) provide a link to the terms of use / license information for the base layer image or shapefile. We cannot publish proprietary or copyrighted maps (e.g. Google Maps, Mapquest) and the terms of use for your map base layer must be compatible with our CC BY 4.0 license.

Note: if you created the map in a software program like R or ArcGIS, please locate and indicate the source of the basemap shapefile onto which data has been plotted.

If your map was obtained from a copyrighted source please amend the figure so that the base map used is from an openly available source. Alternatively, please provide explicit written permission from the copyright holder granting you the right to publish the material under our CC BY 4.0 license.

If you are unsure whether you can use a map or not, please do reach out and we will be able to help you. The following websites are good examples of where you can source open access or public domain maps:

* U.S. Geological Survey (USGS) - All maps are in the public domain. (http://www.usgs.gov)

* PlaniGlobe - All maps are published under a Creative Commons license so please cite u201cPlaniGlobe, http://www.planiglobe.com, CC BY 2.0u201d in the image credit after the caption. (http://www.planiglobe.com/?lang=enl)

* Natural Earth - All maps are public domain. (http://www.naturalearthdata.com/about/terms-of-use/).

9) In the online submission form, you indicated that your data will be submitted to a repository upon acceptance. We strongly recommend all authors deposit their data before acceptance, as the process can be lengthy and hold up publication timelines. Please note that, though access restrictions are acceptable now, your entire minimal dataset will need to be made freely accessible if your manuscript is accepted for publication. This policy applies to all data except where public deposition would breach compliance with the protocol approved by your research ethics board. If you are unable to adhere to our open data policy, please kindly revise your statement to explain your reasoning and we will seek the editor's input on an exemption.

10) Please amend your detailed Financial Disclosure statement. This is published with the article. It must therefore be completed in full sentences and contain the exact wording you wish to be published.

1) State the initials, alongside each funding source, of each author to receive each grant. For example: "This work was supported by the National Institutes of Health (####### to AM; ###### to CJ) and the National Science Foundation (###### to AM)."

2) State what role the funders took in the study. If the funders had no role in your study, please state: "The funders had no role in study design, data collection and analysis, decision to publish, or preparation of the manuscript."

3) If any authors received a salary from any of your funders, please state which authors and which funders..

If you did not receive any funding for this study, please simply state: u201cThe authors received no specific funding for this work.u201d

**Reviewers' Comments:**

Reviewer's Responses to Questions

**Key Review Criteria Required for Acceptance?**

As you describe the new analyses required for acceptance, please consider the following:

**Methods**

-Are the objectives of the study clearly articulated with a clear testable hypothesis stated?

-Is the study design appropriate to address the stated objectives?

-Is the population clearly described and appropriate for the hypothesis being tested?

-Is the sample size sufficient to ensure adequate power to address the hypothesis being tested?

-Were correct statistical analysis used to support conclusions?

-Are there concerns about ethical or regulatory requirements being met?

Reviewer #1: Objectives are clearly articulated - comparing effectiveness of community based continuous delivery of mass dog vaccination with standard team-based delivery. The hypothesis that coverage differs between delivery strategies is clear and testable.

The study is appropriate to address the stated objectives - the 'fried egg' design to reduce contamination is thoughtful. Greater clarity would be good on how representative the team based approach of 'standard practice' is given the modest post-vaccination campaign estimates are. They seem very modest for standard campaigns of this nature which are typically higher in sub saharan Africa.

The population is clearly described and appropriate for the hypothesis being tested in endemic rural African settings. Generalisation beyond this context should be framed cautiously i.e. scaling for larger urban areas or cross border transmission networks would require further evaluation.

The sample size (56 ward arms) appears sufficient for detecting differences in coverage and the power calculations are clearly described. The trial is large enough and statistically robust in coverage - but it is not powered to assess endpoints such as dog rabies incidence or human exposure trends. This doesn't weaken the stated objective of the study - but it possibly limits conclusions about elimination impact.

The statistical analysis looks rigours and appropriate. Additional clarity around assumptions used in estimating 'minimum initial coverage' and probably of falling below 40% would be of added benefit.

Approval of human subjects is clearly documents - it does say some data is available and some is available on request. I think there is a PLOS open data policy so it would need reconciling.

Reviewer #2: The methods appear to have some critical flaws. While the paper presents two study arms that are implied to have been given somewhat equal resources to vaccinate, and a goal of seeing which method has the better sustained coverage, it becomes clear throughout this paper that the community arm had several advantages over the teams-based approach that were not accounted for in the analysis (see below detailed comments). Additionally, the statistical approach undertaken far exceeds the means necessary for such simplistic data. This is a rather simple 2-arm case-control study that relied on equal effort of post-vaccination survey data to compare. This can easily be presented in univariate analysis. The complex bootstrapping approaches provide no added benefit other than to befuddle the reader or potentially hide concerns with small evaluation sample sizes. Most importantly, the study should have included in the analysis a component of resource allocation to study wing and cost-effectiveness of each vaccination approach. The utilization of resources for each study wing are not presented, and therefore it is not possible for the reader to decide if either approach is truly feasible to replicate.

o Line 129 – 131

� I am not sure that the references cited back this statement. The statement seems to be implying that heterogenous coverage across an at-risk population results in sustained or rapidly re-introduced cases. I think the following references better support this claim

• https://www.nature.com/articles/s41598-026-35359-y

• https://www.science.org/doi/10.1126/scitranslmed.aaf6984

• https://sciety.org/articles/activity/10.21203/rs.3.rs-6648701/v1

• Frontiers | Every Dog Has Its Data: Evaluation of a Technology-Aided Canine Rabies Vaccination Campaign to Implement a Microplanning Approach

o Line 133

� Onchocerciasis pharmaceutical intervention is an oral tablet for humans. Rabies intervention is an injectable vaccine for dogs. These are not really comparable, particularly as-written. The barrier is not cold chain in this context, it is that you can easily deliver a community intervention when: 1) humans are the target 2) the medication is oral 3) a licensed or otherwise-registered veterinarian is not required to administer the intervention.

� This is an important sentence, possibly the “thesis sentence” for this study, but it is flawed, as the principal barriers to community-directed implementation are the regulatory, clinical, and biosafety requirements associated with administering injectable biologics for a fatal zoonosis—not solely cold-chain constraints.

o Line 139

� What evidence is there to support that “remote” communities have a sustained risk for rabies presence? Recent papers (see what was shared previously) show with models and empirical surveillance data that truly “remote” or “disconnected” communities are rarely (if ever) affected by rabies. If the premise of this modified dog rabies vaccination approach is necessary, please include some context about the presence of rabies cases in these targeted communities and a definition for what is considered “remote”.

o Lines 146 – 148

� This concept is oversimplified and needs additional context and thoughtfulness. The majority of dog-rabies free countries achieved elimination through mass dog campaigns in the 1920s – 1970s, long before there was reliable cold storage, digital temperature loggers, or even reliable ice boxes. Furthermore, early campaigns utilized even LESS stable nerve tissue vaccines, when compared to the modern-day cell culture vaccines available now.

� While I prefer to avoid a competition for who can find more citations to back one’s claims – my experience is that the vast majority of campaigns and literature cite dog demography, campaign consistency, funding, and political commitment as the key barriers to dog vaccination campaigns. Rarely is temperature stable vaccines listed as a legitimate barrier, when political stability and funding are secure.

� My main point is that the “thesis statements” for this study are over simplified, and put too much emphasis on cold-chain as “THE” barrier to dog rabies vaccination campaigns, with this is simply not true. Thermostable vaccines would be a benefit, but it is not the silver bullet.

o Table 2

� Suggest to present the coverage as a percent, not a decimal

o

o Line 158

� How were they classified as rural or urban? As the authors have established in the introduction that this approach is most applicable to remote communities, understanding how these are defined, particularly among the study areas, is of great importance.

o Figure 1

� While the study utilizes stratified block randomization, the map in Figure 1 displays some geographic clustering of trial arms. Could the authors clarify if they tested for spatial autocorrelation to ensure that the proximity of similar-arm wards did not influence the observed vaccination coverage rates

o Line 201

� Is the most central village somehow demographically different than the other villages? Does these tend to be larger? Easier to reach by road? Better connected? Understanding if these are truly representative of all eligible wards/communities is important.

o Line 285 – 295

� Consider a figure or schematic that visualizes the calendar/timeline and staff involved between the two vaccine distribution methods. I found it a bit difficult to understand the key differences. Also, the terminology “Team” vs “Community” is not very intuitive as to how these two methods differ. I would suggest to use terminology that clearly distinguishes the two methods.

**********

**Results**

-Does the analysis presented match the analysis plan?

-Are the results clearly and completely presented?

-Are the figures (Tables, Images) of sufficient quality for clarity?

Reviewer #1: The analysis presented matches the analysis plan although derivation of the 'minimum initial coverage' required to maintain >40% by year end should be more clearly linked backed to the model assumptions described in the methods - at the moment, the reader must work to connect that. Adjustment for rural/urban imbalance between the arms should be reported to confirm robustness of findings.

The results are clearly presented and logically structured. They are strong but sharpening to prevent over interpretation would be through providing descriptive data on the proportion of dogs reported vaccination but lacking certificates and additional operation context as to why the V1 coverage in the team based approach appears so modest relative to typical immediate post vaccination targets in typical rabies vaccination campaigns.

The figures and tables look of good quality and support interoperation. They show the stability advantage of community based approach - minor improvements would be explicit labelling in supplementary figures to aid rapid understand and a concise table summarising the variance components for easier reader interpretation.

Reviewer #2: o Table 3: The authors utilize human-to-dog ratios (HDR) derived from census data to estimate the total dog population per ward. While this is a common approach, recent studies in similar ecological settings (e.g., Bangladesh) suggest that unowned or free-roaming dogs, which are often missed in household-based census estimates, can comprise a considerable number of total populations (30-40%). If the actual population denominator is higher than estimated, the reported coverage rates would naturally decrease. This has significant implications for the probability of failure analysis in Table 3 where the Team-based arm already shows a 60% probability of falling below the 40% threshold. Could the authors please comment on how the presence of an unowned dog population might influence these estimates?

o Figure 4: Why was the initial coverage in the Team-based arm so low at the start of the cycle? Does this reflect a failure of the "static-point" mobilization strategy compared to the local "One Health Champion" approach?

o Line 532: If the study lacked the power to detect spatial differences, how can we be sure the Community-based approach works equally well in the more challenging urban settings, given that Table 1 showed the Community arm was 82% Rural?

**********

**Conclusions**

-Are the conclusions supported by the data presented?

-Are the limitations of analysis clearly described?

-Do the authors discuss how these data can be helpful to advance our understanding of the topic under study?

-Is public health relevance addressed?

Reviewer #1: The conclusions are supported by the data - the trial demonstrated that the community-based delivery achieved higher and more stable vaccination than the team based approach that was adopted annually across three years. The reduction in probability of coverage falling below the 40% threshold is clearly supported by the modelling. However, the study demonstrates improved vaccination performance not elimination of rabies transmission and better understanding of the team based approach methodology is necessary to fully compare how a community based delivery could be advantageous. The findings are highly relevant to elimination strategies and it is a fantastic study - but it is fair to say they do not directly demonstrate reduction in rabies incidence. Tightening this distinction is important so as to prevent over interpretation.

Some limitations are acknowledged - additional limitations should be addressed such as the imbalance in rural/urban classification, reliance on vaccination certificates for defining coverage, the absence of epidemiological endpoints (eg confirmed rabies incidence in dogs or humans), sustainability and cost implications of scalability. These points don't invalidate the findings but they are important for contextual interpretation.

The manuscript makes a meaningful contribution to implementation science in relation to rabies elimination strategies. It addresses one of the central operational challenges in how vaccination coverage can be maintained above the critical threshold over time in subsaharan Africa. It demonstrates that decentralised vaccination storage and continuous access can stabilise coverage which is particularly relevant for remote and resource constrained settings. It advances understanding of delivery tragedy optimisation which is powerful.

The public health relevance is clearly addressed, clear and timely. Its oddly be strengthened by discussing potential for scalability at national and cross border levels and clarifying how it might integrate into routine government systems without sustained external funding.

Reviewer #2: o Line 557

� Without clearly presenting the resources allocated to each study wing, it is not possible to interpret if one method was more efficient than the other. It could just be that more resources were allocated to the community approach, which of course would result in higher coverage

o Figure 4 and line 559

� This is a recurring theme of my review, but without knowing resources allocated, these conclusions seem more like “common sense” than scientific findings. Figure 4 seems to show an equivalent amount of resources spent at the start of each year in both study wings (both red and blue have large spikes) and then red (community) vaccines are distributed at smaller amounts throughout the year. So it appears that equal resources were not allocated to both approaches to determine if one is more efficient and effective than the other. The real study question should have been, “if resource allocations are equally allocated, is a pulse vaccination (could even be 2-times a year) less effective at maintaining coverage than consistently available vaccination”. If, all resource allocations being equal, the continuous had higher coverages, this would have been a very convincing study on a new approach that directly conflicts with current guidance from the World Health Organization.

o Line 566

� Many high-performing “team based” / “pulse” vaccination campaigns DO reach >61% of dogs during their pulse. What was it about your program’s approach that led to such abysmal “pulse” coverage results, particularly three years in a row for the team approach? These low coverages may reflect campaigns at their infancy, but many rabies programs with some extant of experience will have no problem reaching 61% with an annual campaign approach. What is the struggle is ensuring funding is maintained year on year.

� Also somewhat concerning, it does not look like the number of dogs vaccinated by the team approach increased year-on-year, which is what is expected when vaccination campaigns are adequately supported and implemented in a community. The stagnant vaccination effort for 3-continuous years at sub-par coverages in the “team” approach is highly concerning for the approaches applied by the authors. Many programs would not tolerate consistently low coverage year-on-year, and would therefore likely not find these study results representative of their approaches to pulse vaccination.

o Line 576

� This is really antiquated thinking on dog vaccination in the African setting. Numerous publications have been released in the last decade showing that, with proper resource allocation, dog vaccination coverages can be reached. The challenge has been maintaining resources for the 10 – 15 years it is estimated it takes to eliminate rabies from a large area.

� And along these lines, resource cost and availability (personnel and funding) are a critical component of any health intervention in low- and middle-income countries. It is difficult to interpret results such as those presented here without also presenting the personnel, logistical, and financial resources necessary to operate these two vaccination approaches.

� https://journals.plos.org/plosntds/article?id=10.1371/journal.pntd.0008004 and the experiences in Chad would conflict with the author's statements here.

o Line 579

� Is there any data to show that rabies cases declined, as the theoretical critical vaccination threshold would suggest they should?

o Lines 583 – 595

� This is all theoretical, with no supporting data in the results section. Show the reader the bite data from these communities, definitively prove bite events went down. Show the reader the rabies surveillance data from these communities to prove that vaccination thresholds were met (or not). The authors spend 3 years supporting rabies control in these communities, it is unreasonable that their evaluation should not include direct measures of effect, rather than relying on modeled estimates and (per this paragraph) theoretical musings.

o Line 604

� If community involvement was the key difference between vaccination approaches, that was not clearly explained. As stated earlier, better documentation of the resources allocated to each approach, better explanation of the personnel needed and the timelines of activities, all would make statements such as the one at line 604 easier for the reader to agree with.

o 606 – 610

� What resources (funding) was necessary to utilize the continuous services of these local people? And, while it may be that these people are already paid by other programs and no additional funding was required for the purposes of this study, SOMEONE paid for their time. Surely, they were not volunteers (and it would be ethically questionable if this study asked them to volunteer their time over such a long period). Even if they were paid for by another source, the sustainability of such an approach would require a formalized agreement of resource sharing; did this happen? If so, please make that a prominent feature and success story of this effort; as I would doubt this is reproducible or sustainable without this.

o 618 – 620

� Again, this is an oversimplified and misleading statement. Pulse campaigns also can heavily rely on local leaders and rabies champions to “meaningfully control in their own area”. The difference the authors claim to try to be showing per the stated goals is not the engagement of locals, but the continued presence of vaccine AND the continuously available RESOURCES to make that vaccine available to community members. Implying that pulse-based campaigns are devoid of local leadership is not correct and very few rabies programs would even attempt such a thing.

o Lines 623 – 624

� Exactly – the community approach seems biased from the start and it is unclear if this was due to study design or local implementation factors. Clearly additional resources were made available to the community approach – biasing any direct comparison between methods. A better design would have been to forgo a pulse campaign in the community approach, and rely entirely on continuous availability. However, as it stands, 2 critical limitations seem to be affecting this design:

• First, there appears to be some community-based bias towards the pulse-component of the community approach.

• Second, resource allocation was not equal

o Line 625

� Why could this one health champion not also be involved in these planning activities for a pulse campaign? Again, it seems approaches were not equivalent between the two study arms, particularly when it comes to planning the “pulse” component that both study arms were allowed to conduct. As such, comparison between the two approaches is likely not valid.

o Line 627

� The interplay of what appears to be a study design flaw should not be investigated for future publications. It should be investigated, explained, and accounted for within the study in which the design flaw occurred!

o Line 643

� The authors need to more clearly describe these settings and the definition of “remote”. A goal of the authors was that these results could be applied to similar settings. In my experience, rabies endemic communities with complete lack of "access" to electricity are uncommon. The authors should provide some characterization of these communities in a manner that supports extrapolation to other countries that they deem similar and they need to provide data that establishes that these remote communities are even affected by enzootic dog-mediated rabies in the first place. There was no data presented to suggest that rabies is even present in these settings.

o Line 655

� Agree with this statement, but this does not require zeepots. Many places could/should offer continuously available vaccine AND they DO have electricity. The authors are limiting the relevance of their publication by continuing to imply that continuous vaccination is purely a limitation of thermotolerance. It is not. The limitation is resources, staff, funding, and better knowledge of the Cost-effectiveness of a continuous approach. Unfortunately, none of this is provided in the analysis of this study.

o Line 682

� As much as I want to agree with the authors (and they are probably right) – the study design and lack of consideration of cost/resources makes me doubt if this study truly does show that there is potential in this community delivery method. Flying vaccine by a drone every day to a vaccinator likely would also show “potential”, however the costs to do so make this non-feasible. Without consideration to costs and resources necessary to operate the continuous model, there is little ability to make claims on the feasibility, reproducibility, or acceptability of this approach.

**********

**Editorial and Data Presentation Modifications?**

Use this section for editorial suggestions as well as relatively minor modifications of existing data that would enhance clarity. If the only modifications needed are minor and/or editorial, you may wish to recommend “Minor Revision” or “Accept”.

Reviewer #1: The manuscript is well written and clearly structured.

Suggested minor revisions would be:

Clarifying representativeness of the team-based arm, especially in light of relatively modest V1 coverage estimates compared with typical post-campaign results in similar settings.

Clarifying data availability statement to ensure full alignment with PLOS open data policy (public repository vs “available upon request”).

Report sensitivity adjustment for rural/urban imbalance between arms to confirm robustness of findings.

Provide a brief clarification of modelling assumptions underlying the “minimum initial coverage” and probability of falling below 40%.

Tighten the conclusion language to clearly distinguish improved vaccination coverage from demonstrated interruption of rabies transmission to prevent over interpretation.

Reviewer #2: (No Response)

**********

**Summary and General Comments**

Use this section to provide overall comments, discuss strengths/weaknesses of the study, novelty, significance, general execution and scholarship. You may also include additional comments for the author, including concerns about dual publication, research ethics, or publication ethics. If requesting major revision, please articulate the new experiments that are needed.

Reviewer #1: This is an important, large and well-designed, three-year cluster randomised controlled trial, comparing community-based continuous delivery of mass dog vaccination with standard annual team-based delivery. The objectives are clearly articulated, and the hypothesis - that vaccination coverage differs between delivery strategies - is clear and testable. I found the study engaging, and the operational insights highly interesting.

The study design is appropriate and rigorous. The “fried-egg” approach to minimise contamination is both thoughtful and well justified. The population is clearly described and appropriate for testing the hypothesis in endemic rural African settings. However, generalisation beyond this context should be framed with caution. Scaling to larger urban environments or addressing cross-border transmission networks would require further evaluation. The intervention model is clearly well suited to endemic, decentralised rural systems. Its applicability beyond similar sub-Saharan African contexts should be explicitly qualified in the discussion.

The sample size (56 wards per arm) appears sufficient to detect meaningful differences in coverage, and power calculations are clearly described. The trial is statistically robust for its stated endpoint - vaccination coverage. It is not powered to assess epidemiological outcomes such as confirmed dog rabies incidence or human exposure trends. This doesn’t weaken the study’s objective, but limits conclusions regarding elimination impact, and this distinction should be made explicit.

The statistical analysis is rigorous and appropriate. Additional clarity around assumptions used in estimating the “minimum initial coverage” required to maintain >40% by year end - and the modelling of probability of falling below 40% - would improve interpretability. Adjustment for rural/urban imbalance between trial arms should also be reported (or presented as sensitivity analysis) to confirm robustness of findings.

The results are clearly presented and logically structured. The demonstration of greater stability of coverage in the community-based arm is compelling. To prevent over-interpretation, the manuscript would benefit from:

Providing descriptive data on the proportion of dogs reported vaccinated but lacking certificates.

Providing additional operational context explaining why V1 coverage in the team-based arm appears modest relative to typical immediate post-campaign coverage targets achieved consistently in similar settings.

Clarifying more fully how representative the team-based approach is of “standard practice.”

Figures and tables are of good quality and effectively demonstrate the stability advantage of the community-based approach.

The conclusions are supported by the data presented. The trial clearly demonstrates that community-based delivery achieved higher and more stable vaccination coverage across three years, with a substantially lower probability of falling below the 40% threshold. However, the study demonstrates improved vaccination performance, not elimination of rabies transmission. While highly relevant to elimination strategies, it doesn’t directly demonstrate reductions in rabies incidence. Tightening this distinction would strengthen the manuscript.

Some limitations are acknowledged. Additional limitations that should be explicitly addressed include: rural/urban imbalance between arms; reliance on vaccination certificates for defining coverage; absence of epidemiological endpoints; and sustainability and cost implications for national scalability. These do not invalidate the findings but are important for contextual interpretation.

Overall, this manuscript makes a meaningful and important contribution to implementation science for rabies elimination. It addresses one of the central operational challenges: how vaccination coverage can be maintained above critical thresholds over time in resource-constrained settings. The demonstration that decentralised vaccine storage and continuous access can stabilise coverage is particularly relevant for endemic regions of sub-Saharan Africa.

Public health relevance is clear. Discussion would be further strengthened by consideration of national scalability, cross-border coordination, and integration into routine government systems without sustained external funding.

Reviewer #2: I do believe that continuously available rabies vaccine, as is the model in nearly all upper income countries, is a long-neglected and promising approach to dog vaccination in endemic countries. However, the study design and analytic approach in this manuscript does not convincingly demonstrate that one approach is better than the other and certainly does not show that one is more efficient or effective than the other. The authors spent 3 years in these communities, yet were not able to increase coverages year-on-year using the standard “team” or “pulse” approach – this is highly concerning. These communities also had 3-year sustained vaccination support, but there is no discussion about rabies surveillance activities conducted over this same time frame. Why would the authors rely on modeled and extrapolated dog vaccination coverages (which in themselves have a theoretical cutoff for protection) when instead they could have demonstrated through surveillance whether or not effective herd immunity had been reached. This would have been the most convincing outcome of all. As-presented, the study provides a rather common-sense outcome “if you provide more vaccine, more dogs will get vaccinated”. The resources allocated to each study wing are a critical component of this study and its interpretations, however they are not presented. Showing the cost-effectiveness of these approaches is necessary for this to be publishable.

MAJOR COMMENTS

- Was COST EFFECTIVENESS assessed? Line 138 says there is hope for cost-effective rabies control programs, but it does not appear that costs, resources, or effectiveness (decline in cases) were included in this paper.

- Was any kind of rabies burden measured (e.g., number of human rabies deaths or confirmed canine rabies cases or dog bites treated) before and after the intervention? Ideally, vaccine coverage should be correlated with a decrease in disease burden.

- The results need to include measures of resources allocated to each study wing. Of most importance, how many person-hours were spent under each method to vaccinate these dogs? Without this information, it simply looks like the conclusion is that doing more campaigns throughout the year results in a more stable vaccination coverage; that is not novel or complicated and certainly does not require this level of statistical effort to prove this cause-effect relationship. If the team-approach had been conducted at 2 timepoints through the year, how would that have compared in resource-needs and estimated coverage compared to the community approach? I also find it concerning that the team-approach spent only one day per community. It is well-established that offering vaccination for just one day in remote or hard-to-reach communities is not an effective approach, as owners or dogs may not be available at that specific day or time. Many successful campaigns will spend several (three) days per community to maximize coverage. The resources allocated to each method are not clearly presented, which makes it very difficult as the reader to not only fairly compare the outcomes presented here, but also to understand if the community approach is “better” / “efficient” or if it just represents more vaccination effort.

- Where is the surveillance data for this region? The author spent 3 years supporting rabies control in these communities, surely there is some effort towards surveillance as well? The goal of vaccination is not to achieve a model-derived threshold of coverage – it is to see cases decline. The over-reliance on model and extrapolation-derived vaccination coverages, rather than surveillance data is disappointing. I have a similar concern about the discussion statement about dog vaccination coverages leading to reduced bite events – this was not presented in the results (although probably should be).

- Where are the rest of the references, they only go through 26

- In nearly all countries there are legal requirements on who can administer rabies vaccines. It was not clear who was vaccinating dogs in the community approach, if these were legally-approved vaccinators. If there were local approvals that were unique to this study that allowed non-traditional vaccinators, this is a critical aspect of the approach that needs to be explained in the manuscript. If this was the approach taken, not all countries will be able to get these approvals, but a clearly written example of how this was done in Tanzania would be of great benefit.

- The over-reliance on the statistical approach is not justified given the simplicity of the data collected. Honestly, a simple table of approach and measured coverage by V/Y would have sufficed for the entirety of the analysis.

- The study authors imply a simplistic study design where the only altered variable was the continuous availability of vaccines. However, throughout the methods and discussion it becomes clear that there were several critical variables that differenced between the study wing, including the planning and community sensitization approach and the involvement of local collaborators. These are variables that are not inherent only to continuously available vaccine approaches. Local stakeholders and strong community sensitization pre-campaign can also be achieved during pulse campaign efforts. Since multiple variables were altered between the two study designs, it becomes impossible to distinguish which factors led to the improved vaccination coverages (although there is an element of common sense throughout this entire paper): the differing pre-campaign sensitization? The differing level of community involvement? Differing levels of resources allocated to each wing? The continuous availability of vaccine?

- It is concerning that the “pulse” component of each study arm showed significantly higher coverage in the community approach, when theoretically they should have had similar resource-availability and outcomes for this specific vaccine component. This brings into question if there were incentives given to staff (ie money) and if those incentives differed by study wing. Example, if the team-based staff were paid on salary from the central government but local collaborators in the community-based approach received a payment above and beyond their routine day-job income, then there is an increased incentivization for the community staff when compared to the salaried government workers. Perhaps this was not a concern, however with the lack of transparency of resources allocated to each wing (e.g. funding, personnel, incentives, logistical support), it is impossible for the reader to understand potential bias.

**********

PLOS authors have the option to publish the peer review history of their article (what does this mean?). If published, this will include your full peer review and any attached files.

If you choose “no”, your identity will remain anonymous but your review may still be made public.

**Do you want your identity to be public for this peer review?**  For information about this choice, including consent withdrawal, please see our Privacy Policy.

Reviewer #1: **Yes:**  Luke Gamble

Reviewer #2: No

**Figure resubmission:**

While revising your submission, we strongly recommend that you use PLOS’s NAAS tool (https://ngplosjournals.pagemajik.ai/artanalysis) to test your figure files. NAAS can convert your figure files to the TIFF file type and meet basic requirements (such as print size, resolution), or provide you with a report on issues that do not meet our requirements and that NAAS cannot fix.

After uploading your figures to PLOS’s NAAS tool - https://ngplosjournals.pagemajik.ai/artanalysis, NAAS will process the files provided and display the results in the "Uploaded Files" section of the page as the processing is complete. If the uploaded figures meet our requirements (or NAAS is able to fix the files to meet our requirements), the figure will be marked as "fixed" above. If NAAS is unable to fix the files, a red "failed" label will appear above. When NAAS has confirmed that the figure files meet our requirements, please download the file via the download option, and include these NAAS processed figure files when submitting your revised manuscript.

**Reproducibility:**

To enhance the reproducibility of your results, we recommend that authors of applicable studies deposit laboratory protocols in protocols.io, where a protocol can be assigned its own identifier (DOI) such that it can be cited independently in the future. Additionally, PLOS ONE offers an option to publish peer-reviewed clinical study protocols. Read more information on sharing protocols at https://plos.org/protocols?utm_medium=editorial-email&utm_source=authorletters&utm_campaign=protocols

# Author comment: Author response to Decision Letter 1

**DOI:** 10.1371/journal.pntd.0014704.r003

20 Apr 2026

Attachment Submitted filename: Response to Reviewers_FINAL.docx

# Peer review: Decision Letter 1

**DOI:** 10.1371/journal.pntd.0014704.r004

**Authors:** Annapaola Rizzoli, Philip P. Mshelbwala

27 May 2026

PNTD-D-26-00213R1

Effectiveness of Community-based delivery of mass dog vaccination to prevent rabies: A cluster randomized controlled trial

PLOS Neglected Tropical Diseases

Dear Dr. Lankester,

Thank you for submitting your manuscript to PLOS Neglected Tropical Diseases. After careful consideration, we feel that it has merit but does not fully meet PLOS Neglected Tropical Diseases's publication criteria as it currently stands. Therefore, we invite you to submit a revised version of the manuscript that addresses the points raised during the review process.

Please submit your revised manuscript within by 26th July 2026. If you will need more time than this to complete your revisions, please reply to this message or contact the journal office at plosntds@plos.org. When you're ready to submit your revision, log on to https://www.editorialmanager.com/pntd/ and select the 'Submissions Needing Revision' folder to locate your manuscript file.

Please include the following items when submitting your revised manuscript:

* A letter that responds to each point raised by the editor and reviewer(s). You should upload this letter as a separate file labeled 'Response to Reviewers'. This file does not need to include responses to any formatting updates and technical items listed in the 'Journal Requirements' section below.

* A marked-up copy of your manuscript that highlights changes made to the original version. You should upload this as a separate file labeled 'Revised Manuscript with Track Changes'.

* An unmarked version of your revised paper without tracked changes. You should upload this as a separate file labeled 'Manuscript'.

If you would like to make changes to your financial disclosure, competing interests statement, or data availability statement, please make these updates within the submission form at the time of resubmission. Guidelines for resubmitting your figure files are available below the reviewer comments at the end of this letter.

As the corresponding author, your ORCID iD is verified in the submission system and will appear in the published article. PLOS supports the use of ORCID, and we encourage all coauthors to register for an ORCID iD and use it as well. Please encourage your coauthors to verify their ORCID iD within the submission system before final acceptance, as unverified ORCID iDs will not appear in the published article. Only the individual author can complete the verification step; PLOS staff cannot verify ORCID iDs on behalf of authors.

We look forward to receiving your revised manuscript.

Kind regards,

Philip P. Mshelbwala

Academic Editor

PLOS Neglected Tropical Diseases

Annapaola Rizzoli

Section Editor

PLOS Neglected Tropical Diseases

Shaden Kamhawi

co-Editor-in-Chief

PLOS Neglected Tropical Diseases

orcid.org/0000-0003-4304-636XX

Paul Brindley

co-Editor-in-Chief

PLOS Neglected Tropical Diseases

orcid.org/0000-0003-1765-0002

**Reviewers' Comments:**

Reviewer's Responses to Questions

**Key Review Criteria Required for Acceptance?**

As you describe the new analyses required for acceptance, please consider the following:

**Methods**

-Are the objectives of the study clearly articulated with a clear testable hypothesis stated?

-Is the study design appropriate to address the stated objectives?

-Is the population clearly described and appropriate for the hypothesis being tested?

-Is the sample size sufficient to ensure adequate power to address the hypothesis being tested?

-Were correct statistical analysis used to support conclusions?

-Are there concerns about ethical or regulatory requirements being met?

Reviewer #1: The objectives of the study are clearly articulated, and the hypothesis - that vaccination coverage differs between community-based continuous delivery and annual team-based delivery - is clear and testable. The study addresses an important operational question relevant to rabies elimination strategies in endemic settings.

The cluster-randomised design is generally appropriate, and the 'fried-egg' approach to minimise contamination between intervention arms is clever and well justified. The study population is clearly described and appropriate for assessing vaccination delivery strategies in predominantly rural endemic regions of sub-Saharan Africa. However, applicability beyond similar agro-ecological and operational settings should continue to be framed cautiously, particularly in larger urban environments, pastoral systems, or regions with more complex cross-border dog movement dynamics.

The sample size appears appropriate and adequately powered for the primary endpoint of vaccination coverage. The statistical analysis looks rigorous and appropriately conducted, with useful sensitivity analyses included. Additional clarification regarding assumptions underlying the modelling of minimum initial coverage and the probability of falling below the 40% threshold has improved the manuscript.

However, there remain important methodological concerns regarding the interpretation of the comparator arm. The manuscript increasingly frames the community-based approach as operationally superior, yet the team-based intervention described does not appear representative of many highly effective team-based rabies vaccination campaigns currently operating in Africa and Asia.

Numerous programmes routinely achieve substantially higher coverage using static-point and team-based approaches without extensive infrastructure, refrigeration access, or high-cost operational models. The description implying that standard team-based approaches depend primarily on electricity, refrigeration hubs, and centrally based vaccinators doesn't fully reflect operational realities in many successful field programmes.

Similarly, the team structure employed in the comparator arm appears operationally limited relative to best-practice mass dog vaccination delivery and may therefore underestimate the achievable effectiveness of well-organised team-based campaigns under comparable resource-constrained conditions. This requires more balanced discussion and clearer qualification throughout the manuscript.

An additional methodological limitation relates to vaccination verification. The manuscript relies heavily on vaccination certificates to determine coverage estimates. While the sensitivity analysis is useful, the Methods should better justify why alternative field-based identification approaches were not incorporated.

In many large-scale rabies vaccination campaigns, temporary paint marking is used as a rapid, practical, highly economical, and operationally scalable method for identifying vaccinated dogs during post-vaccination surveys and transects. Such approaches avoid reliance on owner-retained documentation, which may introduce variability associated with certificate loss or inconsistent presentation. The authors should explain whether temporary marking methods were considered and why they were not included in the study design.

Finally, although the manuscript discusses operational efficiency and potential scalability of the intervention, the absence of accompanying cost-effectiveness and surveillance outcome data substantially limits interpretation of comparative programme value. Given that broader implementation claims are now made within the Discussion, presentation of at least preliminary comparative operational cost data within the current manuscript would considerably strengthen interpretation of the findings.

Reviewer #2: (No Response)

**********

**Results**

-Does the analysis presented match the analysis plan?

-Are the results clearly and completely presented?

-Are the figures (Tables, Images) of sufficient quality for clarity?

Reviewer #1: The analyses presented broadly match the stated analysis plan and are generally reported clearly. The results are logically structured, and the primary finding - that the community-based arm achieved higher and more stable vaccination coverage over time - is consistently demonstrated across the analyses presented.

The inclusion of sensitivity analyses using stricter vaccination definitions strengthens the robustness of the findings, and the additional clarification around modelling assumptions has improved interpretability. The presentation of temporal variation and the probability of coverage falling below the 40% threshold is particularly useful in illustrating differences in stability between intervention arms.

Figures and tables are generally of good quality and support interpretation effectively. The graphical presentation of coverage trends over time and threshold probabilities is clear and accessible. Minor improvements to supplementary figure labelling and simplification of some statistical presentation would probably further aid rapid interpretation by readers.

However, important concerns remain regarding interpretation of the comparator arm and the operational conclusions drawn from the data. The relatively modest coverage achieved in the team-based arm appears substantially lower than that achieved by many established mass dog vaccination programmes operating in Africa and Asia using team-based and static-point approaches. As a result, it remains difficult to determine to what extent the observed differences reflect genuine superiority of the community-based strategy versus limitations in the implementation of the comparator intervention itself.

Related to this, the manuscript still does not provide comparative operational cost data or epidemiological surveillance outcomes. While the authors indicate these analyses will be published separately, the current manuscript makes broader operational and scalability claims that are difficult to fully evaluate without at least preliminary comparative cost information. It is therefore challenging for the reader to assess whether the higher coverage achieved through the community-based approach was obtained more efficiently, more economically, or simply through greater operational input over time.

There also remains concern regarding vaccination verification methodology. Reliance on vaccination certificates alone may introduce variability associated with certificate retention and presentation. The manuscript would benefit from greater discussion of practical alternative field approaches, such as temporary paint marking, which are widely used in rabies vaccination campaigns because they are rapid, inexpensive, and operationally scalable for post-vaccination assessment.

Overall, the results are important and potentially highly relevant to rabies elimination strategies. However, stronger qualification of the comparator limitations and inclusion of additional operational context are necessary to avoid over-interpretation of comparative effectiveness claims.

Reviewer #2: (No Response)

**********

**Conclusions**

-Are the conclusions supported by the data presented?

-Are the limitations of analysis clearly described?

-Do the authors discuss how these data can be helpful to advance our understanding of the topic under study?

-Is public health relevance addressed?

Reviewer #1: The conclusions are partially supported by the data presented. The study clearly demonstrates that, within the operational framework tested, the community-based approach achieved higher and more stable vaccination coverage over time than the comparator team-based approach. The data also support the finding that coverage in the community-based arm was less likely to fall below the theoretical critical threshold during the annual cycle.

However, the manuscript at times moves beyond the evidence directly generated by the study. The trial demonstrates differences in vaccination coverage performance, but it does not directly demonstrate rabies elimination, reduced rabies transmission, reductions in human exposure, or improved public health outcomes. These distinctions should remain clearly separated throughout the Discussion and Conclusions to avoid overinterpretation.

Although additional limitations have now been acknowledged, some important limitations still require stronger emphasis. In particular:

1. The comparator team-based intervention may not fully represent the performance achievable by well-organised team-based rabies vaccination programmes operating elsewhere in Africa and Asia.

2. The study was conducted within predominantly rural endemic settings in northern Tanzania, limiting generalisability beyond similar sub-Saharan African contexts.

3. vaccination verification relied heavily on certificates rather than alternative field-identification approaches such as temporary paint marking.

4. epidemiological surveillance outcomes and comparative cost-effectiveness data are not presented within the current manuscript.

The absence of comparative operational cost data is especially important because the manuscript increasingly discusses scalability, sustainability, and programmatic superiority of the intervention. Without accompanying cost analyses, it remains difficult to fully evaluate whether the observed gains in coverage were achieved more efficiently or simply through greater operational input over time.

This said, the manuscript nevertheless makes an important contribution to implementation science and operational rabies control. It addresses one of the major practical challenges in rabies elimination programmes: maintaining vaccination coverage above critical thresholds in remote and resource-constrained settings. The findings are therefore highly relevant to ongoing discussions around delivery optimisation for endemic regions.

Public health relevance is clearly addressed and the topic is timely and important given global 'Zero by 30' ambitions. However, broader claims regarding scalability and suitability for national elimination strategies should be more cautiously framed until supported by accompanying economic analyses, surveillance outcomes, and evaluation across a wider range of epidemiological and operational settings.

Reviewer #2: (No Response)

**********

**Editorial and Data Presentation Modifications?**

Use this section for editorial suggestions as well as relatively minor modifications of existing data that would enhance clarity. If the only modifications needed are minor and/or editorial, you may wish to recommend “Minor Revision” or “Accept”.

Reviewer #1: The manuscript is generally well written and substantially improved from the original submission. The figures and tables are clear and support interpretation effectively. However, several editorial and presentation issues should still be addressed to improve balance, clarity, and interpretability:

1. The wording describing “standard” team-based delivery should be moderated. Some statements imply that cold-chain dependence, limited infrastructure, and low coverage are universally representative of team-based rabies vaccination programmes in Africa. This is not consistently true, including in large-scale field programmes operating successfully in remote settings without fixed refrigeration infrastructure.

2. The Discussion should more clearly distinguish between the specific comparator tested in this trial and broader team-based approaches used elsewhere in Africa and Asia.

3. Greater caution should be used when discussing scalability and superiority of the community-based model in the absence of comparative cost-effectiveness and surveillance outcome data within the current manuscript.

4. The manuscript would benefit from inclusion of a brief operational comparison table summarising the practical differences between the two delivery strategies, including personnel structure, vaccination frequency, storage approach, and follow-up activities.

5. Additional clarification should be included regarding how vaccinated dogs were identified during post-vaccination assessment. The limitations of certificate-based verification are discussed, but simple field-based temporary marking approaches (e.g. livestock paint markings), which are inexpensive and operationally practical in mass dog vaccination campaigns, should at least be acknowledged.

6. Some supplementary figures and tables would benefit from clearer labelling to improve rapid interpretation by readers unfamiliar with the study design.

7. Minor editorial tightening of the Discussion would improve readability by reducing repetition around thermotolerance and community engagement concepts.

Reviewer #2: (No Response)

**********

**Summary and General Comments**

Use this section to provide overall comments, discuss strengths/weaknesses of the study, novelty, significance, general execution and scholarship. You may also include additional comments for the author, including concerns about dual publication, research ethics, or publication ethics. If requesting major revision, please articulate the new experiments that are needed.

Reviewer #1: This is an important, ambitious and operationally highly relevant three-year cluster randomised controlled trial addressing one of the central challenges in canine rabies elimination: maintaining vaccination coverage above critical herd immunity thresholds in endemic settings. The manuscript is clearly written, statistically sophisticated, and tackles a genuinely important implementation problem with substantial potential public health relevance.

The revised manuscript is substantially improved and many previous concerns have been addressed thoughtfully. However, following review of the revised text and author responses, I consider that several remaining issues materially affect interpretation of the findings and the strength of the conclusions being drawn. For this reason, I now believe Major Revision is more appropriate prior to acceptance.

The principal concern relates to the framing and representativeness of the comparator 'team-based' arm. The manuscript repeatedly presents the comparator as representative of standard team-based rabies vaccination delivery across Africa. However, many successful large-scale rabies control programmes operating in Africa - including highly resource-efficient field programmes working in remote areas without fixed refrigeration infrastructure - routinely achieve substantially higher coverage using team-based static-point approaches than those reported here. As currently framed, the manuscript risks implying that the relatively low coverage observed in the comparator arm is inherent to team-based delivery itself, rather than reflecting the specific implementation model adopted within this trial. This distinction is important and should be addressed more carefully and neutrally throughout the manuscript.

Related to this, several statements discussing logistical limitations of team-based delivery appear overly generalised, particularly around dependency on fixed electricity and refrigeration infrastructure. In practice, many programmes operate effectively using passive cooling systems, temporary cold-chain management, mobile vaccination teams and highly decentralised operational structures. The current wording risks overstating the novelty or operational advantage of the intervention by underrepresenting the flexibility and effectiveness of alternative field delivery approaches already used successfully at scale.

The study convincingly demonstrates improved and more stable vaccination coverage under the tested community-based delivery model. However, the manuscript occasionally moves beyond the data in suggesting implications for elimination impact, scalability and superiority of delivery strategy without simultaneously presenting the associated comparative operational costs and epidemiological outcomes. The absence of integrated cost-effectiveness analysis is a significant limitation because the practical value of any delivery model depends not only on achieved coverage but on the resources, personnel structure, supervision requirements and sustainability needed to achieve it. While I appreciate that economic analyses are planned separately, the current manuscript makes interpretive claims that are difficult to fully evaluate in the absence of at least comparative operational cost summaries within the same paper.

Similarly, while vaccination coverage is an entirely appropriate primary endpoint, the manuscript should remain cautious in linking these findings to elimination impact without accompanying surveillance outcomes. The distinction between improved vaccination performance and demonstrated reduction in rabies transmission should remain explicit and carefully maintained throughout.

I also believe the manuscript should more fully acknowledge the limitations of certificate-based vaccination verification. The discussion appropriately notes limitations of collars, microchips and facial recognition, but practical field marking methods such as temporary livestock paint markings - widely used in mass vaccination campaigns because they are rapid, inexpensive and operationally simple for post-vaccination assessment - are not discussed and should at least be acknowledged as highly effective alternative operational approaches.

Finally, applicability beyond the specific epidemiological and operational context studied should remain carefully qualified. The intervention appears particularly suited to decentralised rural endemic settings typical of parts of sub-Saharan Africa. Generalisation to dense urban settings, highly mobile dog populations, or different sociocultural contexts requires further evidence.

Overall, this remains a valuable and potentially influential contribution to rabies implementation science. However, I believe the framing, contextualisation, and interpretation of the findings require further revision before the conclusions can be fully supported at the level currently presented.

Reviewer #2: I cannot support publication of a paper where the data necessary to evaluate the impact of the study design is being withheld; to be published separately. It sounds like the authors have all of the cost and surveillance data necessary to demonstrate the feasibility (cost) and effectiveness (surveillance) of these two approaches, but have chosen not to include that critical information.

The authors repeat a statement that both approaches could be implemented by local authorities. The reader cannot validate that claim if the resources (cost) necessary to operate these two vaccination approaches are not clearly stated within this manuscript.

**********

PLOS authors have the option to publish the peer review history of their article (what does this mean?). If published, this will include your full peer review and any attached files.

If you choose “no”, your identity will remain anonymous but your review may still be made public.

**Do you want your identity to be public for this peer review?**  For information about this choice, including consent withdrawal, please see our Privacy Policy.

Reviewer #1: No

Reviewer #2: No

[NOTE: If reviewer comments were submitted as an attachment file, they will be attached to this email and accessible via the submission site. Please log into your account, locate the manuscript record, and check for the action link "View Attachments". If this link does not appear, there are no attachment files.]

**Figure resubmission:**

While revising your submission, we strongly recommend that you use PLOS’s NAAS tool (https://ngplosjournals.pagemajik.ai/artanalysis) to test your figure files. NAAS can convert your figure files to the TIFF file type and meet basic requirements (such as print size, resolution), or provide you with a report on issues that do not meet our requirements and that NAAS cannot fix. After uploading your figures to PLOS’s NAAS tool - https://ngplosjournals.pagemajik.ai/artanalysis, NAAS will process the files provided and display the results in the "Uploaded Files" section of the page as the processing is complete. If the uploaded figures meet our requirements (or NAAS is able to fix the files to meet our requirements), the figure will be marked as "fixed" above. If NAAS is unable to fix the files, a red "failed" label will appear above. When NAAS has confirmed that the figure files meet our requirements, please download the file via the download option, and include these NAAS processed figure files when submitting your revised manuscript.

**Reproducibility:**

To enhance the reproducibility of your results, we recommend that authors of applicable studies deposit laboratory protocols in protocols.io, where a protocol can be assigned its own identifier (DOI) such that it can be cited independently in the future. Additionally, PLOS ONE offers an option to publish peer-reviewed clinical study protocols. Read more information on sharing protocols at https://plos.org/protocols?utm_medium=editorial-email&utm_source=authorletters&utm_campaign=protocols

# Author comment: Author response to Decision Letter 2

**DOI:** 10.1371/journal.pntd.0014704.r005

24 Jun 2026

Attachment Submitted filename: Response_to_Reviewers_FINAL_auresp_2.docx

# Peer review: Decision Letter 2

**DOI:** 10.1371/journal.pntd.0014704.r006

**Authors:** Annapaola Rizzoli, Philip P. Mshelbwala

23 Jul 2026

PNTD-D-26-00213R2Effectiveness of Community-based delivery of mass dog vaccination to prevent rabies: A cluster randomized controlled trialPLOS Neglected Tropical Diseases Dear Dr. Lankester, Thank you for submitting your manuscript to PLOS Neglected Tropical Diseases. After careful consideration, we feel that it has merit but does not fully meet PLOS Neglected Tropical Diseases's publication criteria as it currently stands. Therefore, we invite you to submit a revised version of the manuscript that addresses the points raised during the review process. Please submit your revised manuscript by 23rd August 2026.  If you will need more time than this to complete your revisions, please reply to this message or contact the journal office at plosntds@plos.org. When you're ready to submit your revision, log on to https://www.editorialmanager.com/pntd/ and select the 'Submissions Needing Revision' folder to locate your manuscript file. Please include the following items when submitting your revised manuscript:* A letter that responds to each point raised by the editor and reviewer(s). You should upload this letter as a separate file labeled 'Response to Reviewers'. This file does not need to include responses to any formatting updates and technical items listed in the 'Journal Requirements' section below.* A marked-up copy of your manuscript that highlights changes made to the original version. You should upload this as a separate file labeled 'Revised Manuscript with Track Changes'.* An unmarked version of your revised paper without tracked changes. You should upload this as a separate file labeled 'Manuscript'. If you would like to make changes to your financial disclosure, competing interests statement, or data availability statement, please make these updates within the submission form at the time of resubmission. Guidelines for resubmitting your figure files are available below the reviewer comments at the end of this letter. As the corresponding author, your ORCID iD is verified in the submission system and will appear in the published article. PLOS supports the use of ORCID, and we encourage all coauthors to register for an ORCID iD and use it as well. Please encourage your coauthors to verify their ORCID iD within the submission system before final acceptance, as unverified ORCID iDs will not appear in the published article. Only the individual author can complete the verification step; PLOS staff cannot verify ORCID iDs on behalf of authors.We look forward to receiving your revised manuscript. Kind regards, Philip P. MshelbwalaAcademic EditorPLOS Neglected Tropical Diseases Annapaola RizzoliSection EditorPLOS Neglected Tropical Diseases

Shaden Kamhawi

co-Editor-in-Chief

PLOS Neglected Tropical Diseases

orcid.org/0000-0003-4304-636XX

Paul Brindley

co-Editor-in-Chief

PLOS Neglected Tropical Diseases

orcid.org/0000-0003-1765-0002

**Additional Editor Comments (if provided):**     **Journal Requirements:**

If the reviewer comments include a recommendation to cite specific previously published works, please review and evaluate these publications to determine whether they are relevant and should be cited. There is no requirement to cite these works unless the editor has indicated otherwise. [Note: HTML markup is below. Please do not edit.] **Reviewers' comments:**  Reviewer's Responses to Questions

**Key Review Criteria Required for Acceptance?**

As you describe the new analyses required for acceptance, please consider the following:

**Methods**

-Are the objectives of the study clearly articulated with a clear testable hypothesis stated?

-Is the study design appropriate to address the stated objectives?

-Is the population clearly described and appropriate for the hypothesis being tested?

-Is the sample size sufficient to ensure adequate power to address the hypothesis being tested?

-Were correct statistical analysis used to support conclusions?

-Are there concerns about ethical or regulatory requirements being met?

Reviewer #1: The revised manuscript has addressed the majority of my previous methodological concerns. One minor point remains regarding the comparator. The team-based arm represents a specific government delivery model rather than the full spectrum of contemporary team-based mass dog vaccination programmes. I encourage the authors to make this distinction explicit, as well-managed field team approaches have achieved substantially higher post-campaign coverage in other endemic settings.

**********

**Results**

-Does the analysis presented match the analysis plan?

-Are the results clearly and completely presented?

-Are the figures (Tables, Images) of sufficient quality for clarity?

Reviewer #1: The analyses presented are consistent with the analysis plan. The results are clearly presented and the figures and tables are of good quality. I have no major concerns with the presentation of the results.

**********

**Conclusions**

-Are the conclusions supported by the data presented?

-Are the limitations of analysis clearly described?

-Do the authors discuss how these data can be helpful to advance our understanding of the topic under study?

-Is public health relevance addressed?

Reviewer #1: The conclusions are supported by the data presented and are generally appropriately balanced. The manuscript now more clearly acknowledges the principal limitations of the study, and the discussion highlights the contribution of the findings to improving mass dog vaccination delivery in endemic settings. Public health relevance is clear and well articulated. I would encourage one final clarification that the findings demonstrate improved vaccination coverage within the study setting rather than direct evidence of rabies elimination, and that applicability beyond similar decentralised rural systems should be interpreted with appropriate caution.

**********

**Editorial and Data Presentation Modifications?**

Use this section for editorial suggestions as well as relatively minor modifications of existing data that would enhance clarity. If the only modifications needed are minor and/or editorial, you may wish to recommend “Minor Revision” or “Accept”.

Reviewer #1: The manuscript remains well written and clearly presented. The figures and tables are of good quality and support interpretation of the findings. I encourage the authors to make it explicit that the comparator represents a specific government team-based delivery model rather than the full spectrum of contemporary team-based mass dog vaccination programmes, to avoid over-generalisation of the findings. Additionally, the discussion should continue to emphasise that the results are most directly applicable to decentralised rural endemic settings typical of sub-Saharan Africa, and that extrapolation to other epidemiological or operational contexts should be made cautiously. Finally, a brief clarification of the assumptions underpinning the estimation of the minimum initial vaccination coverage required to maintain coverage above the 40% threshold would further improve clarity.

**********

**Summary and General Comments**

Use this section to provide overall comments, discuss strengths/weaknesses of the study, novelty, significance, general execution and scholarship. You may also include additional comments for the author, including concerns about dual publication, research ethics, or publication ethics. If requesting major revision, please articulate the new experiments that are needed.

Reviewer #1: This remains an important and well-executed cluster randomised controlled trial addressing one of the key operational challenges in canine rabies elimination: how to achieve and maintain effective vaccination coverage in endemic settings. The manuscript is well written, the study design is robust, and I found the operational insights both interesting and highly relevant.

The authors have responded constructively to the previous review comments, and the manuscript has been strengthened as a result. In particular, the discussion now provides a more balanced interpretation of the findings and better acknowledges the study's limitations.

My remaining comments are minor and relate primarily to framing rather than methodology. I encourage the authors to continue making it explicit that the comparator represents a specific government team-based delivery model rather than the full spectrum of contemporary team-based mass dog vaccination approaches. Likewise, while the findings provide strong evidence that the community-based approach improves and maintains vaccination coverage, they should continue to distinguish this from direct evidence of rabies elimination and emphasise that the findings are most directly applicable to decentralised rural endemic settings typical of sub-Saharan Africa.

Overall, I believe this manuscript makes a valuable contribution to the evidence base for rabies control and implementation science. Subject to these minor clarifications, I support publication.

**********

PLOS authors have the option to publish the peer review history of their article (what does this mean?). If published, this will include your full peer review and any attached files.

If you choose “no”, your identity will remain anonymous but your review may still be made public.

**Do you want your identity to be public for this peer review?**  For information about this choice, including consent withdrawal, please see our Privacy Policy.

Reviewer #1: No

[NOTE: If reviewer comments were submitted as an attachment file, they will be attached to this email and accessible via the submission site. Please log into your account, locate the manuscript record, and check for the action link "View Attachments". If this link does not appear, there are no attachment files.] **Figure resubmission:**   While revising your submission, we strongly recommend that you use PLOS’s NAAS tool (https://ngplosjournals.pagemajik.ai/artanalysis) to test your figure files. NAAS can convert your figure files to the TIFF file type and meet basic requirements (such as print size, resolution), or provide you with a report on issues that do not meet our requirements and that NAAS cannot fix.

After uploading your figures to PLOS’s NAAS tool - https://ngplosjournals.pagemajik.ai/artanalysis, NAAS will process the files provided and display the results in the "Uploaded Files" section of the page as the processing is complete. If the uploaded figures meet our requirements (or NAAS is able to fix the files to meet our requirements), the figure will be marked as "fixed" above. If NAAS is unable to fix the files, a red "failed" label will appear above. When NAAS has confirmed that the figure files meet our requirements, please download the file via the download option, and include these NAAS processed figure files when submitting your revised manuscript. **Reproducibility:**  To enhance the reproducibility of your results, we recommend that authors of applicable studies deposit laboratory protocols in protocols.io, where a protocol can be assigned its own identifier (DOI) such that it can be cited independently in the future. Additionally, PLOS ONE offers an option to publish peer-reviewed clinical study protocols. Read more information on sharing protocols at https://plos.org/protocols?utm_medium=editorial-email&utm_source=authorletters&utm_campaign=protocols

# Author comment: Author response to Decision Letter 3

**DOI:** 10.1371/journal.pntd.0014704.r007

29 Jul 2026

Attachment Submitted filename: Response document.docx

# Editor report: Decision Letter 3

**DOI:** 10.1371/journal.pntd.0014704.r008

**Authors:** Annapaola Rizzoli, Philip P. Mshelbwala

26 Aug 2026

Dear Lankester,

We are pleased to inform you that your manuscript 'Effectiveness of Community-based delivery of mass dog vaccination to prevent rabies: A cluster randomized controlled trial' has been provisionally accepted for publication in PLOS Neglected Tropical Diseases.

Before your manuscript can be formally accepted you will need to complete some formatting changes, which you will receive in a follow up email. A member of our team will be in touch with a set of requests.

Please note that your manuscript will not be scheduled for publication until you have made the required changes, so a swift response is appreciated.

IMPORTANT: The editorial review process is now complete. PLOS will only permit corrections to spelling, formatting or significant scientific errors from this point onwards. Requests for major changes, or any which affect the scientific understanding of your work, will cause delays to the publication date of your manuscript.

Should you, your institution's press office or the journal office choose to press release your paper, you will automatically be opted out of early publication. We ask that you notify us now if you or your institution is planning to press release the article. All press must be co-ordinated with PLOS.

Thank you again for supporting Open Access publishing; we are looking forward to publishing your work in PLOS Neglected Tropical Diseases.

Best regards,

Philip P. Mshelbwala

Academic Editor

PLOS Neglected Tropical Diseases

Ricardo Soares Magalhaes

Section Editor

PLOS Neglected Tropical Diseases

Shaden Kamhawi

co-Editor-in-Chief

PLOS Neglected Tropical Diseases

orcid.org/0000-0003-4304-636XX

Paul Brindley

co-Editor-in-Chief

PLOS Neglected Tropical Diseases

orcid.org/0000-0003-1765-0002

***********************************************************

# Editor report: Acceptance letter

**DOI:** 10.1371/journal.pntd.0014704.r009

**Authors:** Annapaola Rizzoli, Philip P. Mshelbwala

Dear Lankester,

We are delighted to inform you that your manuscript, "Effectiveness of Community-based delivery of mass dog vaccination to prevent rabies: A cluster randomized controlled trial," has been formally accepted for publication in PLOS Neglected Tropical Diseases.

We have now passed your article onto the PLOS Production Department who will complete the rest of the publication process. All authors will receive a confirmation email upon publication.

The corresponding author will soon be receiving a typeset proof for review, to ensure errors have not been introduced during production. Please review the PDF proof of your manuscript carefully, as this is the last chance to correct any scientific or type-setting errors. Please note that major changes, or those which affect the scientific understanding of the work, will likely cause delays to the publication date of your manuscript. Note: Proofs for Front Matter articles (Editorial, Viewpoint, Symposium, Review, etc...) are generated on a different schedule and may not be made available as quickly.

Soon after your final files are uploaded, the early version of your manuscript will be published online unless you opted out of this process. The date of the early version will be your article's publication date. The final article will be published to the same URL, and all versions of the paper will be accessible to readers.

For Research Articles, you will receive an invoice from PLOS for your publication fee after your manuscript has reached the completed accept phase. If you receive an email requesting payment before acceptance or for any other service, this may be a phishing scheme. Learn how to identify phishing emails and protect your accounts at https://explore.plos.org/phishing.

Thank you again for supporting open-access publishing; we are looking forward to publishing your work in PLOS Neglected Tropical Diseases.

Best regards,

Shaden Kamhawi

co-Editor-in-Chief

PLOS Neglected Tropical Diseases

Paul Brindley

co-Editor-in-Chief

PLOS Neglected Tropical Diseases
